# Novel Short PEG Chain-Substituted Porphyrins: Synthesis, Photochemistry, and In Vitro Photodynamic Activity against Cancer Cells

**DOI:** 10.3390/ijms231710029

**Published:** 2022-09-02

**Authors:** Dawid Lazewski, Malgorzata Kucinska, Edward Potapskiy, Joanna Kuzminska, Artur Tezyk, Lukasz Popenda, Stefan Jurga, Anna Teubert, Zofia Gdaniec, Jacek Kujawski, Katarzyna Grzyb, Tomasz Pedzinski, Marek Murias, Marcin Wierzchowski

**Affiliations:** 1 Department of Chemical Technology of Drugs, Poznan University of Medical Sciences, Grunwaldzka 6 Street, 60-780 Poznan, Poland; 2 Department of Toxicology, Poznan University of Medical Sciences, Dojazd 30 Street, 60-631 Poznan, Poland; 3 Department of Pharmaceutical Chemistry, Poznan University of Medical Sciences, Grunwaldzka 6 Street, 60-780 Poznan, Poland; 4 Department of Forensic Medicine, Poznan University of Medical Sciences, Swiecickiego 6 Street, 60-781 Poznan, Poland; 5 NanoBioMedical Centre, Adam Mickiewicz University in Poznan, Wszechnicy Piastowskiej 3 Street, 61-614 Poznan, Poland; 6 Institute of Bioorganic Chemistry, Polish Academy of Sciences, Noskowskiego 12/14 Street, 61-704 Poznan, Poland; 7 Department of Organic Chemistry, Poznan University of Medical Sciences, Grunwaldzka 6 Street, 60-780 Poznan, Poland; 8 Faculty of Chemistry, Adam Mickiewicz University in Poznan, Uniwersytetu Poznanskiego 8 Street, 61-614 Poznan, Poland; 9 Center for Advanced Technology, Adam Mickiewicz University in Poznan, Uniwersytetu Poznanskiego 10 Street, 61-614 Poznan, Poland

**Keywords:** porphyrins, photodynamic therapy, polyethylene glycol, singlet oxygen, cancer cells

## Abstract

This work presents the synthesis and characterization of metal-free, zinc (II), and cobalt (II) porphyrins substituted with short PEG chains. The synthesized compounds were characterized by UV-Vis, ^1^H and ^13^C NMR spectroscopy, and MALDI-TOF mass spectrometry. The origin of the absorption bands for tested compounds in the UV-Vis range was determined using a computational model based on the electron density functional theory (DFT) and its time-dependent variant (TD-DFT). The photosensitizing activity was evaluated by measuring the ability to generate singlet oxygen (ΦΔ), which reached values up to 0.54. The photodynamic activity was tested using bladder (5637), prostate (LNCaP), and melanoma (A375) cancer cell lines. In vitro experiments clearly showed the structure–activity relationship regarding types of substituents, their positions in the phenyl ring, and the variety of central metal ions on the porphyrin core. Notably, the metal-free derivative **3** and its zinc derivative **6** exerted strong cytotoxic activity toward 5637 cells, with IC_50_ values of 8 and 15 nM, respectively. None of the tested compounds induced a cytotoxic effect without irradiation. In conclusion, these results highlight the potential value of the tested compounds for PDT application.

## 1. Introduction

The first human study showing the potential of photodynamic therapy (PDT) as an anticancer strategy started in 1976, when Snell and Kelly used a hematoporphyrin derivative mixture (HpD) for treating patients with bladder cancer [1]. In 1978, Dougherty reported the clinical application of PDT using a HpD for treating different tumors in patients. This work was a milestone in the PDT field, increasing the industrial and scientific interest in developing PDT as an anticancer approach. To date, PDT has been shown as an effective modality in treating malignant and pre-malignant skin cancers [2], Barrett’s esophagus [3], unresectable cholangiocarcinoma [4], head and neck [5], lung [6,7], and bladder cancers [8]. PDT is also used for non-oncological purposes to treat dermatological diseases (e.g., acne, photoaging, and psoriasis), actinic keratosis [2,9], ophthalmological diseases (e.g., age-related macular degeneration, central serous chorioretinopathy, polypoidal choroidal vasculopathy, and choroidal hemangioma) [10], and dental diseases [11]. However, the high expectations of PDT were verified by reality, and this therapeutic approach has primarily been limited to clinical trials or palliative care and cannot achieve the status of standard cancer treatment [12]. Compared to standard chemotherapy, PDT needs to consider several factors, such as tissue optics and limited light penetration, thus requiring a well-optimized protocol [13]. Nevertheless, huge progress in the PDT field in the last years regarding the design of novel and better photosensitizers (PSs), development of more efficient light sources and delivery systems, and a better understanding of the dosimetry and photokilling mechanism have brought us closer to overcoming the most critical limitations to an efficient therapeutic approach [14]. All these efforts in the future can improve the outcome for cancer patients.

**Figure 1:**
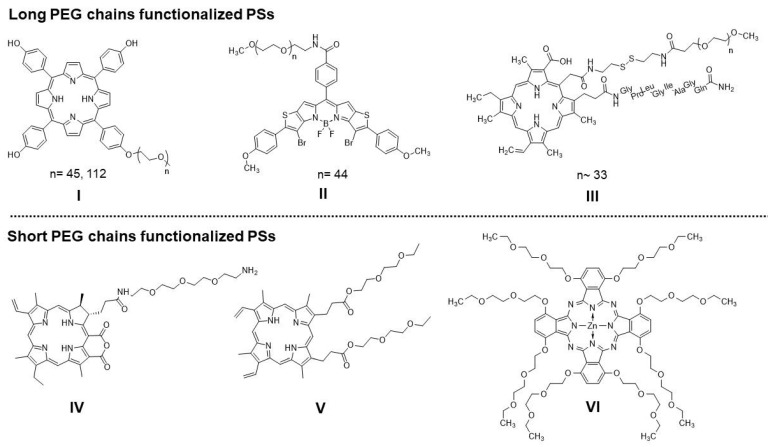
Recently synthesized PEG-functionalized photosensitizers with long PEG chains: I—5,10,15,20-tetrakis(4-hydroxyphenyl)porphyrin (p-THPP) [39], II—3, 7-dibromo-2, 8-di (4-methoxyphenyl)-11-(phenyl-4-carbamoylo-PEG_2000_)-dithieno [2, 3-b]-[3, 2-g]-5, 5- difluoro-5-bora-3a, 4a-diaza-s-indacene) [40], and III—PEGylated Chlorin e6 polypeptide [41]; and short PEG chains: IV—Purpurin-PEG3-Amine Zinc Complex [38], V—2,7,12,18-tetramethyl-13,17-bis(1,4,7-trioxanonylcarbonylethyl)-3,8-divinylporphyrin [35], and VI—[1,4,8,11,15,18,22,25-octakis(1,4,7-trioxanonyl)phthalocyanine]zinc(II) [36].

As described above, several problems limit the clinical application of PDT, which result from both physicochemical properties (e.g., a highly hydrophobic character, the high tendency for aggregation in aqueous solutions, photobleaching, and low absorption in the IR or NIR spectrum) [15] and the specific tumor microenvironment (e.g., hypoxia, high antioxidative system, and compensatory pathways) [16,17]. Most photosensitizers show a hydrophobic character, resulting in aggregation in an aqueous environment and poor water solubility [18]. These issues limit the therapeutic effect of PDT and impede systemic administration. On the other hand, the hydrophobic character ensures better penetration by the cell membrane [19] and localization in cellular compartments [20], which is crucial for effective damage to cancer cells. Thus, many efforts have been made to find a compromise between the physicochemical properties and biological activity to retain the expected clinical outcomes. Numerous studies have reported that the chemical modification of photosensitizers through incorporating hydrophilic or amphiphilic substituents to peripheral positions or the core of the macrocycles, as well as appropriate encapsulation, might increase the water solubility. The most widely used substituents are quaternary ammonium salts, sulfonyl, hydroxyl, carboxyl, and a primary amine group [21]. Polymers are extensively used components to improve the physiological and chemical stability, biocompatibility, and cytotoxic activity of PSs [22]. To date, numerous polymers, such as poly(ethylene glycol) (PEG), polydopamine (PDA), and poly(lactic-co-glycolic acid) (PLGA), have been used in PDT [22]. Functionalization of PSs with PEG is particularly interesting and offers several advantages. PEG is a highly water-soluble, flexible, uncharged, and biocompatible polymer widely used in the pharmaceutical industry and is already approved for different drugs [23]. In 1990, the first approved PEG–protein conjugate (PEG–adenosine deaminase, Adagen^®^) appeared in the market to treat severe combined immunodeficiency caused by an inherited deficiency of the adenosine deaminase enzyme [23]. Since then, several PEG–protein conjugates have been approved for hepatitis C, acute lymphatic leukemia, age-related macular degeneration, anemia associated with chronic kidney disease, and rheumatoid arthritis [23,24]. The success of the PEG–protein conjugate led to the development of polymer–small molecule drug conjugates for both cancer and non-cancer indications. PEGylated Irinotecan, Cisplatin, Camptothecin, or Paclitaxel are currently in clinical trials [23]. PEGylation is widely used in the PDT field, mainly in terms of the design of the photosensitizer nanocarriers [25,26,27,28]. However, PEGylation might also be used as a direct modification to improve the pharmacokinetic properties and cytotoxic effect [29,30]. Large PEG pendants have found their way into photosensitizer modifications in recent years thanks to their potential for making any molecule water-soluble. However, their large molecular mass and large difference between the lowest and highest mass significantly impact elimination from the body, and long-chain PEGs might induce an immune response [31]. As was described in the literature, the molecular weight of the PEG molecule used was conversely related to renal clearance [32,33]. The chain length also affects the circulation half-life, which prolongs with increases in molecular weight [34]. Conversely, short PEG chains can dramatically affect photosensitizer activity, as shown in our previous works [35,36]. As Hamblin and co-workers described it, PEGylation decreased the aggregation and reduced oxygen consumption during irradiation, which suggested a switch in photochemical mechanism from type II (singlet oxygen) to type I (radicals or electron transfer) [37]. Pavlíčková et al. synthesized novel Purpurin 18 derivatives with a polyethylene glycol linker with strong cytotoxic activity against cervical, prostate, breast, and pancreatic cancer cells [38]. The structure of the photosensitizers substituted with PEG already reported in the literature are present in Figure 1.

Due to the constant search for new and improved anticancer therapy, PDT offers several benefits over “classical” treatment. It is less invasive than surgery and causes fewer side effects than traditional chemotherapy due to localized treatment [11]. Since photosensitizers play a crucial role in PDT, particular attention is paid to designing new structures and improving the already-known active structures. This paper showed the synthesis of PEG-functionalized porphyrins containing zinc, cobalt, and their free-metal counterparts, which are dedicated to the anticancer PDT approach. In our synthetic strategy, we introduce PEG chains by modifying the Adler–Longo method and carefully characterize the structures using UV-Vis, ^1^H and ^13^C NMR spectroscopy, and MALDI-TOF mass spectrometry. We performed the computational modeling based on the electron density functional theory (DFT) and in its time-dependent variant (TD-DFT) to explain the origin of the absorption bands for our compounds in the UV-Vis range. The tested photosensitizers’ photophysical and singlet oxygen generation properties were investigated via absorption and phosphorescence spectroscopy, respectively. The dark- and light-dependent cytotoxicity of these photosensitizers were evaluated on human bladder carcinoma (5637), prostate carcinoma (LNCaP), and malignant melanoma (A375) cell lines. Overall, the main aim of the presented study was the synthesis and characterization of new photosensitizers and the assessment of their action against neoplastic cells.

## 2. Results and Discussion

### 2.1. Synthesis

**Figure 2:**
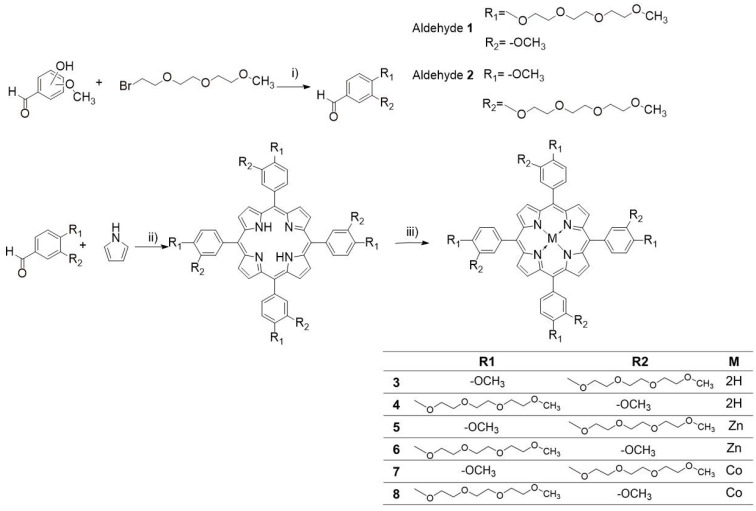
General synthetic scheme for the aldehyde substrates and porphyrins: (i) K_2_CO_3_, DMF, 80 °C, 24 h; (ii) propionic acid, 140 °C, 24 h; (iii) metal salt, DMF, 80 °C, 24 h.

Aldehydes were prepared using the Williamson ether synthesis method utilizing phenol and alkyl bromide following the standard nucleophilic substitution mechanism [42]. The reaction is simple and affords high yields of 79–83%. We have decided to utilize the modified Adler–Longo method [43] for synthesizing PEGylated porphyrins. While it employs harsher conditions than the newer Lindsey method [44], we found it is easier to work within our case. It is simpler in principle and afforded a significantly less tarry post-reaction mixture, which was easier to purify, with yields of 28–33%. It also requires smaller amounts of organic solvents. The synthesis scheme is presented in Figure 2. Alkylating aldehydes, as opposed to porphyrins, lets us avoid purifying a mixture of variously substituted porphyrins. Yields from metallation of the free porphyrins ranged from 56 to 93%, likely dependent on the salt utilized; zinc acetate is more soluble in the organic solvent than cobalt(II) chloride. Alternatively, the acetate anion is a stronger conjugate base than the chloride anion, which may better facilitate the metallation of porphyrins.

### 2.2. NMR Spectroscopy

All newly synthesized aldehydes and porphyrins were analyzed using 1D and 2D NMR techniques.

Chemical shifts (δ) are quoted in parts per million (ppm) and are referred to as a residual solvent peak. Coupling constants (J) are quoted in Hertz (Hz). The abbreviations s, d, dd, t, pt, and m refer to the singlet, doublet, doublet of doublets, triplet, pseudo-triplet, and multiplet, respectively. The resonance assignments were based on ^1^H, ^13^C, and 2D ^1^H–^1^H COSY, ^1^H–^13^C HSQC, and ^1^H–^13^C HMBC experiments.

**Figure 3:**
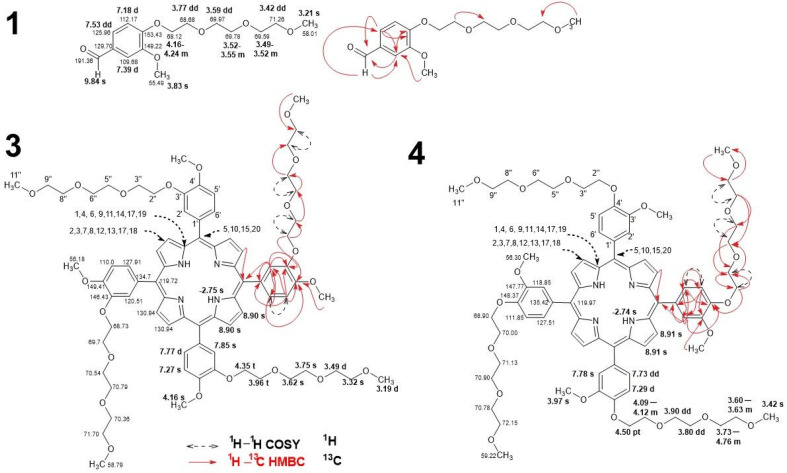
NMR data of aldehyde **1** and porphyrins **3** and **4**. Values in bold are for ^1^H NMR while the normal text assigned to ^13^C NMR. Chemical shifts are expressed as (ppm) in DMSO-d6 and CDCl_3_. Key HMBC and ^1^H–^1^H COSY correlations are marked with dashed arrows and bold lines, respectively.

The recognized signals of the exemplary aldehyde **1** and two constitutional isomers of the new porphyrins **3** and **4** are shown in Figure 3. The most characteristic groups of signals are described below. Signals represent the aldehydes carbonyl groups at 9.83 (**2**) and 9.84 (**1**) ppm in the proton spectra and 191.32 and 191.36 ppm in carbon, respectively. The signals of other structural fragments, such as the benzene ring, polyether chain, and methoxy group, reveal a significant similarity to the porphyrin ones and will not be described separately.

The macrocyclic core signals of both constitutional isomers **3** and **4** show considerable similarity. In both cases, the chemical shifts of the protons connected to the nitrogen atoms N1 and N3 of the inner core are similar and are observed at −2.74 ppm and −2.75 ppm, respectively. The *meso* carbons (positions C5, C10, C15, and C20) give signals at 119.72 ppm (compound **3**) and 119.97 ppm (compound **4**). In the case of isomer **3** and isomer **4** for the C2, C3, C7, C8, C12, C13, C17, and C18 positions, a singlet signal with the integration of 8 protons is observed. The signal is observed for porphyrin **3** at 8.90 ppm and porphyrin **4** at 8.91 ppm. In compound **3**, the presence of quaternary carbons in positions C1, C4, C6, C9, C11, C14, C17, and C19 were represented by very weak spots in ^13^C NMR experiments at 130.94 ppm. The same signal is not observed for compound **4**. Aromatic rings in the ^1^H NMR spectrum represent three multiplets. In the case of porphyrin **3** is a singlet of C2′ at 7.75 ppm, doublet of C6′ at 7.77 ppm, and a singlet at 7.27 for C5′. The corresponding multiplets for porphyrin **4** in the same atomic positions are a singlet at 7.78 ppm, doublet of doublets at 7.73, and a doublet at 7.29 ppm. The ^13^C NMR spectra of **3** reveal the following signals for carbons atom in position C1′- C6′: 134.70 ppm, 120.51 ppm, 146.43 ppm, 149.41 ppm, 110.00 ppm, 127.91 ppm. The corresponding C1′- C6′ atoms signals of compound **4** are at: 135.42 ppm, 118.85 ppm, 147.77 ppm, 148.37 ppm, 111.85 ppm, and 127.51 ppm. The four methoxy groups in porphyrin **3** and **4** are observed as singlets with the integration of 12 protons at 4.16 ppm and 3.97 ppm, respectively. The corresponding ^13^C signals are observed at 56.18 and 56.30 ppm.

The polyether substituent represents at the following positions: a methoxy group in position 11″ and trioxyethylene fragment at 2″, 3″, 5″, 6″, 8″ and 9″. The chain end methoxy group in 11″ of **3** and **4** are observed as two overlapped singlets at 3.20 ppm and singlets at 3.42 ppm, respectively. The observed multiplicity of signals of protons in positions 2″, 3″, 5″, 6″, 8″, and 9″ of compounds **3** and **4** vary despite similar values of the observed chemical shift. These signals are observed for compound **3** as a triplet at 4.35 ppm, triplet at 3.96 ppm, singlet at 3.62 ppm, singlet at 3.75 ppm, doublet at 3.49 ppm, and singlet at 3.32 ppm. The corresponding signals of porphyrin **4** are observed as a pseudo-triplet at 4.50 ppm, multiplet 4.09–4.12 ppm, doublet of doublets at 3.90 ppm, doublet of doublets at 3.80 ppm, multiplet 3.73–3.76 ppm, and multiplet 3.60–363 ppm. All NMR spectra are included in the Supplementary Materials in Figures S1–S30.

### 2.3. Mass Spectrometry with Fragmentation

To confirm the identity of the porphyrin compounds, the Matrix-Assisted Laser Desorption Ionization (MALDI) technique coupled with a Time-of-Flight analyzer (TOF) was used. Application of the MALDI TOF/TOF allowed for observation of further fragmentation of the molecular ions. This technique successfully identifies macromolecular compounds such as proteins, peptides, DNA, and polymers [45,46,47]. Exemplary mass spectra of compound **3** are shown in Figure S33, with masses noted for the most abundant ions. The mass spectra of aldehyde **1** and **2** are presented in Figures S1 and S2. The MALDI TOF results for all compounds are presented in the Supplementary Materials (Figures S31–S43).

**Figure 4:**
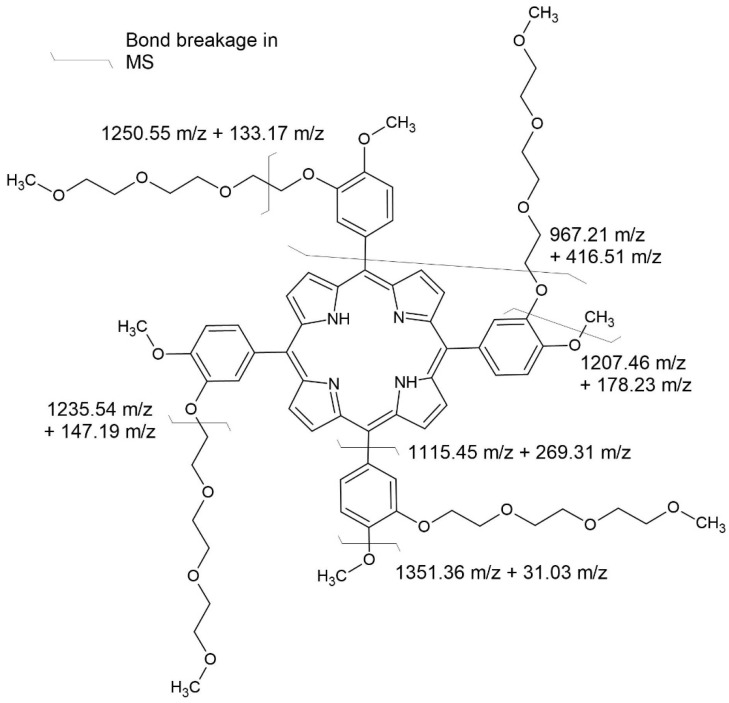
Fragmentation of compound **3**.

During these experiments, we noticed remarkable stability of the porphyrin core ring. As shown in Figure 4, primarily during fragmentation, the bonds in the PEG chains were broken. The most abundant ion registered is 1235.46 m/z, representing fragmentation between the oxygen and C2″ in the PEG chain. The second most-abundant ion, except for the parent ion, is 1250.56 m/z, which corresponds to fragmentation in the middle of the first –CH_2_–CH_2_– unit of the PEG chain. The other most-prominent ions are 1351.36 m/z, representing the elimination of the methoxyl group, most likely from the phenyl ring and not the end of the PEG chain. However, it is impossible to determine that at this moment. It is worth noting that, at most, only one phenyl ring breaks off from the porphyrin ring, represented by a 1115.45 m/z ion. After the phenyl ring is eliminated, the molecule fragments further by breaking at the second PEG chain after the first oxygen, just like in the most-abundant ion.

### 2.4. UV-Vis Spectroscopy and Simulation

**Figure 5:**
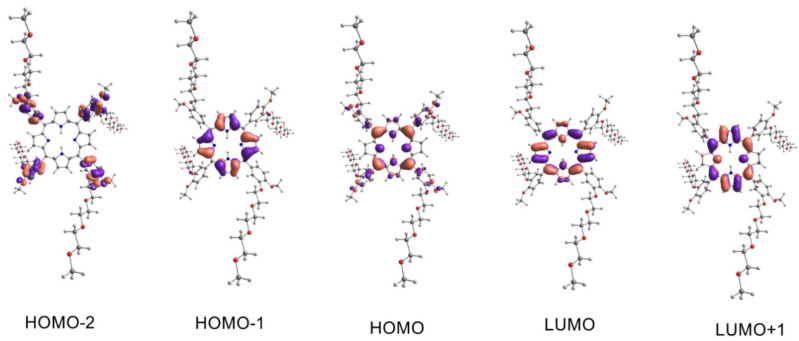
The molecular orbitals involved as the main component in the electronic transitions of **3**. Spatial functions representing orbitals were computed by application of PCM/TD-DFT/PBE1PBE/6-31+G(d,p)//PBE1PBE/6-31G(d,p) with a contour value 0.03 a.u. The isosurfaces marked by the violet and salmon colors represent parts of the orbitals in different phases.

**Figure 6:**
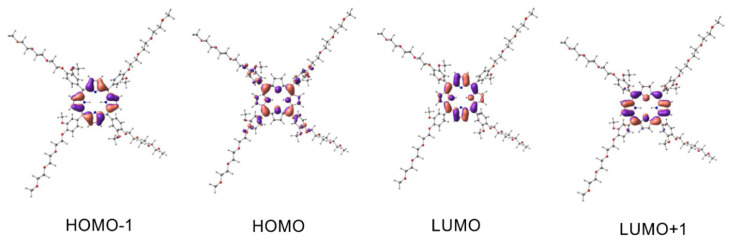
The molecular orbitals involved as the main component in the electronic transitions of **4**. Spatial functions representing orbitals were computed by application of PCM/TD-DFT/PBE1PBE/6-31+G(d,p)//PBE1PBE/6-31G(d,p) with a contour value 0.03 a.u. The isosurfaces marked by violet and salmon colors represent parts of orbitals in different phases.

**Figure 7:**
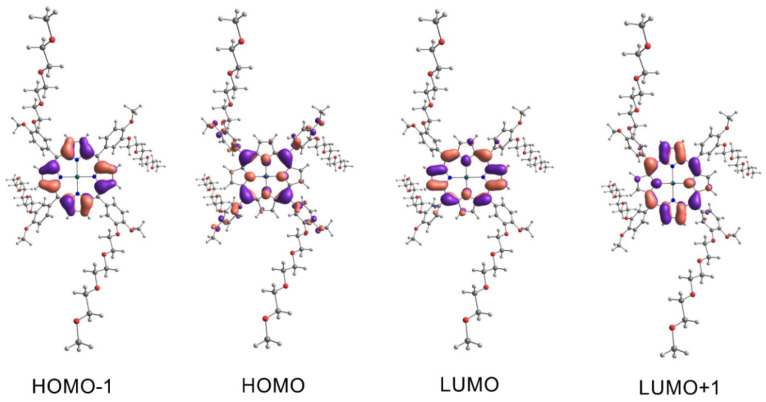
The molecular orbitals involved as the main component in the electronic transitions of **5**. Spatial functions representing orbitals were computed by application of PCM/TD-DFT/PBE1PBE/6-31+G(d,p)//PBE1PBE/6-31G(d,p) with a contour value 0.03 a.u. The isosurfaces marked by violet and salmon colors represent parts of orbitals in different colors represent parts of orbitals in different phases.

The UV-Vis spectra of porphyrins have two bands in the characteristic regions, namely, the Q band and B band (Soret band). The appearance of these bands in the spectrum, according to Guterman’s theory, is connected with electron transfer due to excitation between the two Highest Occupied Molecular Orbitals (HOMO-1, HOMO) and the two Lowest Unoccupied Molecular Orbitals (LUMO, LUMO+1) [48]. To explain the origin of the absorption bands for our compounds in the UV-Vis range, we proposed a computational model based on the electron density functional theory (DFT) and in its time-dependent variant (TD-DFT). The global minima of porphyrin **3–5** were found with the DFT model—correlation-exchange functional PBE1PBE and basis functions 6-31G(d,p). The 6-31+G(d,p) basis function was chosen in the DT-DFT simulations because of its relatively low computational cost. This function was successfully applied in the metal–ligand (metal–dye) TD-DFT simulations [49] and phthalocyanine–metal complexes [50]. A hybrid functional exchange-correlation PBE1PBE was chosen, which gave accurate results in the TD-DFT simulation of the metal–ligand complexes [51,52], including phthalocyanines [53]. For computational cost reasons, we have limited our simulation to closed-shell systems such as porphyrins **3–5**. The effect of a solvent (0DMF) on the energy of the absorption bands was simulated using the Polarizable Continuum Model in an integral equation formalism variant—IEFPCM. The calculation results are presented in Table 1, Table 2 and Table 3 and Figure 5, Figure 6 and Figure 7. The essential vertical excitations for presence in the Q band and B band are presented below, and the fully computed results for the twenty excited states analysis are present in the Supplementary Materials.

The computational model gave surprisingly consistent results with empirically observed data. The largest shift was observed for the B band of complex compound **5** and was 11 nm (experiment λ_max_ 431 nm—simulation 420 nm). The computational model confirmed the largest contribution of the HOMO-1, HOMO, LUMO, and LUMO+1 orbitals to the formation of the Q band and B band postulated by Gouterman. First, simulated transitions in the Q band of free porphyrin **3** at 592 nm revealed 73% HOMO → LUMO and 24% HOMO-1 → LUMO+1 character. Similarly, for porphyrin **4** at 597 nm, we observed a contribution of 76% HOMO → LUMO and 22% HOMO-1 → LUMO+1. In the next transition of compound **3** at 557 nm, we observed a contribution of 73% HOMO → LUMO+1 and 25% HOMO-1 → LUMO. The maximum at 564 nm in the Q band of **4** computational model attributed to 77% of HOMO → LUMO+1 and 22% of HOMO-1 → LUMO character. Because of the similar energy of the LUMO and LUMO+1 atomic orbitals of complex **5**, we observe only one wavelength at 563 nm. Two electron transitions with compositions are responsible for the band mentioned above—transition one 71% HOMO → LUMO and 24% HOMO-1 → LUMO+1; transition two 71% HOMO → LUMO+1 and 24% HOMO-1 → LUMO. The electron transitions not predicted by the computational model for derivative **3** at 519 nm and 651 nm, compound **4** at 518 nm and 651 nm, and complex **5** at 602 nm are bands with vibronic character. Their presence is caused by molecular vibrations of porphyrinoids [54,55]. The superposition of two-electron transitions forms the simulated Soret band at 428 nm for porphyrin **3**, transition 3 with contributions of 43% HOMO-1 → LUMO+1, 21% HOMO-2 → LUMO, and transition 4 with 37% HOMO-1 → LUMO, 26% HOMO-2 → LUMO. Similarly, transitions 3 and 4 are responsible for the simulated band at 426 nm for porphyrin **4**. Their contribution respectively: transition 3–43% HOMO-1 → LUMO+1 and transition 4–48% HOMO-1 → LUMO. In the case of complex **5**, the appearance of the band at 420 nm is connected to transition 3: contributions of 38% HOMO-1 → LUMO+1, 26% HOMO-1 → LUMO, and transition 4: contributions of 38% HOMO-1 → LUMO, 26% HOMO-1 → LUMO+1.

**Figure 8:**
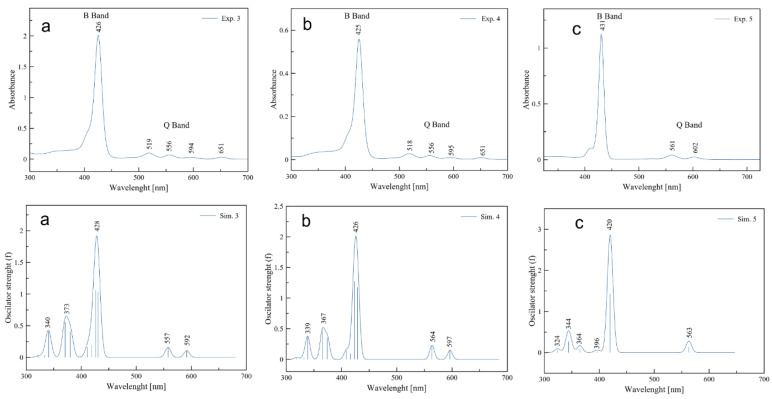
Simulated and experimental UV-Vis spectra in N, N-dimethylformamide of compounds **3** (**a**); **4** (**b**) and **5** (**c**). Upper spectra are experimental while lower are simulated. The applied FWHM parameter of the simulation curves was equal to 12.

All porphyrins exhibit strong absorption in the Soret region of the spectrum at around 425–430 nm. The simulated and experimental UV-Vis spectra are shown in Figure 8. The UV-Vis spectra of all compounds are presented in the Supplementary Materials (Figures S50–S55). Additionally, there are a few weak maximums in the Q band region depending on the compound. Free-base porphyrins have four at 518, 555, 591, and 650 nm. Zinc complexes have two at 552 and 594 nm, while cobalt complexes at 538 and 588 nm.

Undoubtedly, the maximum absorption at 425 nm can be considered as potential limitation for clinic tumor treatment. As was excellent discussed by Kessel, photosensitizers excited by light below 630 nm may have minimal application in photodynamic therapy [14]. This study presented the compounds with maximum absorbance at 425 nm. However, this limitation can become an advantage. This type of photosensitizer can be useful in developing self-illuminating PDT systems. The idea of internal light sources offers novel possibilities for depth-independent PDT [56]. To date, different internal light sources, such as chemiluminescence, bioluminescence, and Cerenkov radiation, have been proven capable of exciting certain photosensitizers to produce reactive oxygen species and exert cytotoxic effects [57,58]. As shown by Laptev et al., photosensitizers with a maximum peak at 412 nm could be “activated” by chemiluminescence emitted by luminol which has an emission band at 350–550 nm [59]. Thus, our porphyrin-based PSs, functionalized with PEG chains, could be the starting point for designing novel structures and light system delivery. Moreover, the selectivity of by an internal PDT system might be increased by appropriate formulation that will be dedicated to markers presented only or overexpressed in cancer cells. Also, a fiber optic device might solve the problem with tissue penetration by light at a wavelength of 425 nm.

On the other hand, blue light excitation is widely used to treat skin diseases, such as basal cell carcinoma [60] or melanoma [61]. Moreover, several compounds excited at 425–500 nm are still considered photosensitizers for PDT, including natural compounds, e.g., curcumin [62], berberine [63], parietin [64], or synthetic PSs, e.g., iridium(III)-based complexes [65]. In 2022, Desgranges at el. presented a novel class of amphiphilic PpIX derivatives possessing two PEG550 headgroups to increase hydrophilicity and two hydrogenated or hemifluorinated tails to ensure the hydrophobic character [66]. This chemical modification increases solubility in water. These novel compounds were also irradiated with light in the region of 410–500 nm with a maximum of around 440 nm. The authors showed that PpIX derivatives could be considered promising amphiphilic photosensitizers. Interestingly, blue light-activated photosensitizer can also be used to purify bone marrow (BM) before transplantation, as Čunderlíková et al. described it [67]. The PDT can be used to purge the bone marrow of remaining malignant cells for autologous hematopoietic stem cell transplantation (auto-HSCT) [68]. HSCT is a therapeutic approach for hematologic malignancies such as leukemia, multiple myeloma and lymphoma, and solid tumors e.g., breast and ovarian cancer [68]. The authors showed that hexaminolevulinate (HAL)-based PDT might effectively eliminate cancer cells from the BM while sparing BM progenitor cells needed for hematopoietic reconstruction [67]. It should also be emphasized that exposure to blue light has been used for the blue-light cystoscopy (BLC), also known as fluorescence cystoscopy or photodynamic diagnosis, which enables imaging of the urinary bladder [69]. HAL is the only agent approved in the USA and Europe for BLC photosensitization. The installation of HAL in bladder cancer leads to cellular porphyrin formation, which fluoresces red when illuminated with blue light with a wavelength of 360–450 nm [69]. Thus, the visible lesions can be resected more precisely. Therefore, even the lights at wavelengths between 400–600 nm have limited tissue penetration, the modern PDT can find applications for blue light, and the development of nanomedicine and drug delivery systems might resolve the problem with light delivery to the target tissue.

### 2.5. Singlet Oxygen Generation

**Figure 9:**
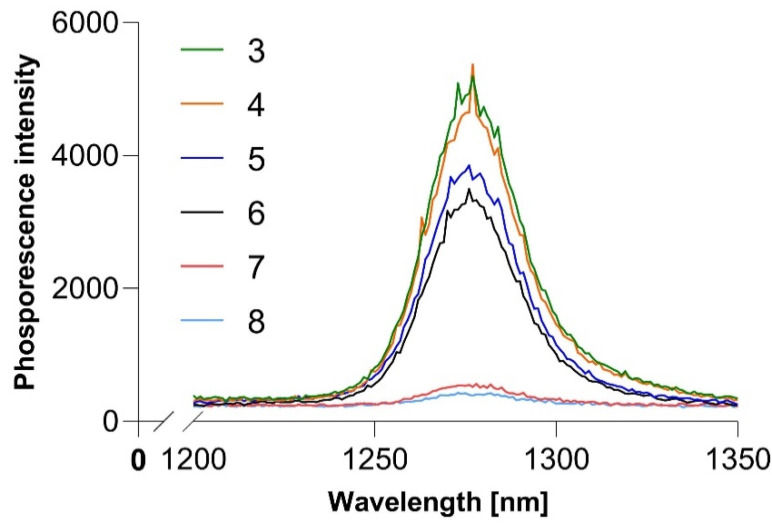
Singlet oxygen phosphorescence in DMF.

Using singlet oxygen phosphorescence at approximately 1270 nm, we measured the singlet oxygen-generating ability of our synthesized compounds. The results are shown in Figure 9 and Table 4. We can observe that free porphyrins generate the most singlet oxygen as determined by their phosphorescence intensity. Zinc porphyrins generate about 50% less singlet oxygen, and cobalt porphyrins generate less than 20% of the free-base porphyrins. Singlet oxygen quantum yield was calculated in reference to meso-tetra(4-N-methylpyridyl)porphine (TmPyP). These results contradict our expectations that zinc porphyrins would be the most efficient generators as they contain relatively the heaviest atom in the series and one with a “closed shell.”

### 2.6. Biological Activity

**Figure 10:**
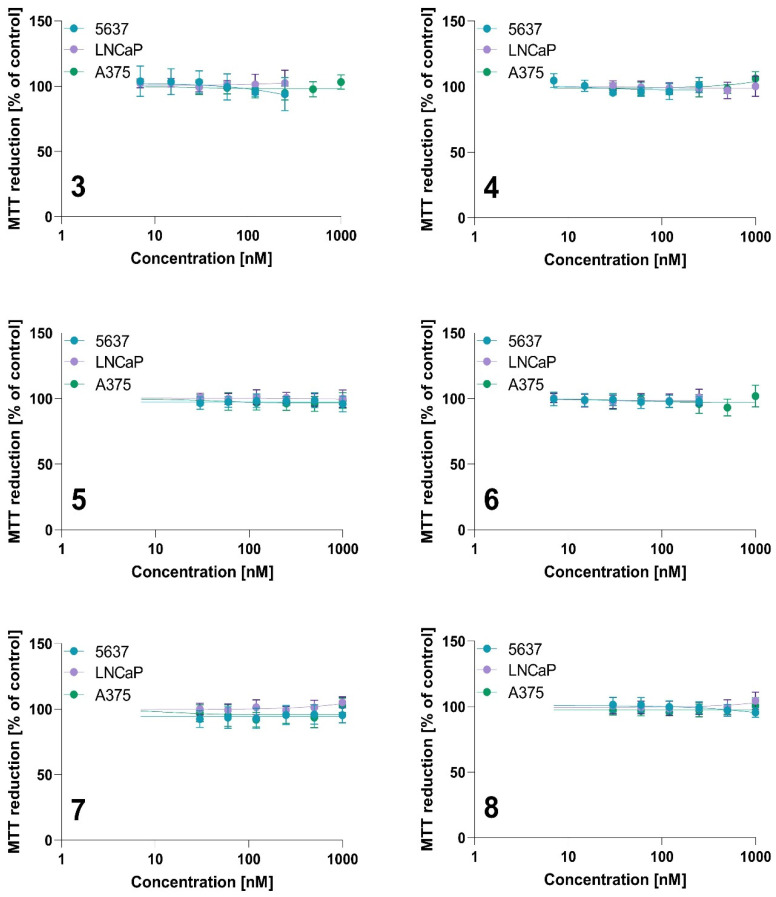
The dark toxicity of tested photosensitizers against 5637, LNCaP, and A375 cells for compounds **3**, **4**, **5**, **6**, **7** and **8**. Data are expressed as the mean ± SD from at least three independent experiments.

**Figure 11:**
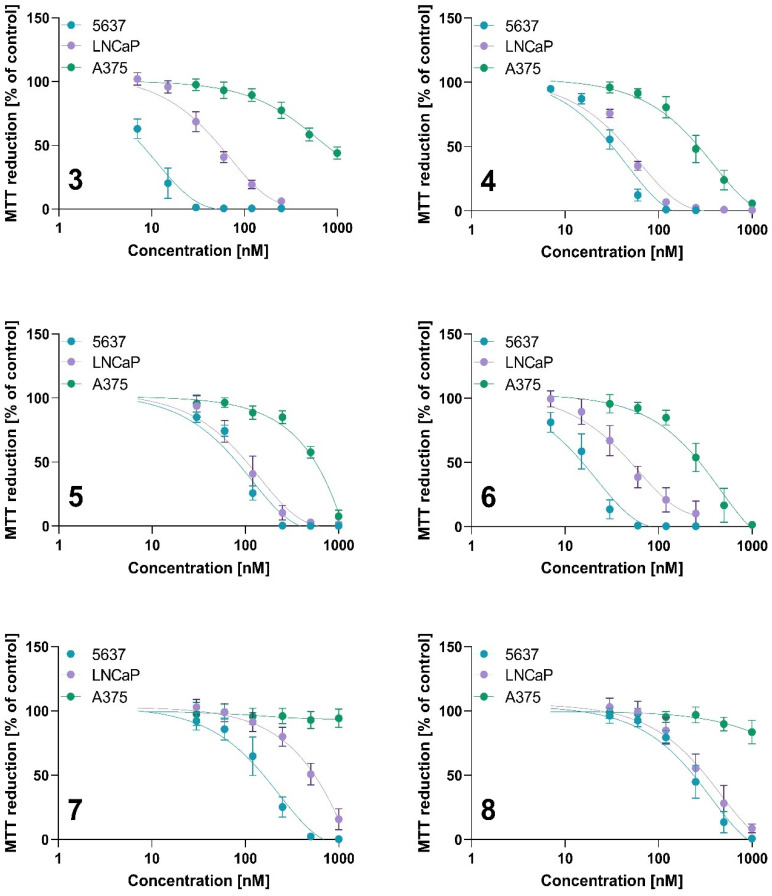
The phototoxicity of tested photosensitizers against 5637, LNCaP, and A375 cells for compounds **3**, **4**, **5**, **6**, **7** and **8**. After incubation with tested PSs, cells were irradiated at a light dose of 10 J/cm^2^. Cytotoxic effect was determined using the MTT assay. Data are expressed as the mean ± SD from at least three independent experiments.

The photodynamic activity was measured using the MTT assay, and the results are presented in Table 5 and Figure 10 and Figure 11. All tested compounds did not exert a cytotoxic effect without irradiation in tested concentration ranges (Figure 10).

The results of in vitro activity studies correlate with singlet oxygen generation. Derivatives containing cobalt in their structure show the lowest singlet oxygen generation, and both tested compounds showed the lowest activity against cancer cells. Interestingly, A375 melanoma cells were completely resistant to cobalt-based derivatives at the tested concentration range (Table 5, Figure 11). However, cobalt derivatives were active against bladder and prostate cancer cells with IC_50_ values below 0.6 µM. The highest activity was observed against 5637 cells treated with **3** (IC_50_ value of 8.01 ± 2.12 nM), free base 4-methoxy-3-(1,4,7,10-tetraoxoundecyl)phenyl derivative. This compound also exerted strong cytotoxic activity against LNCaP cells (IC_50_ value of 49.71 ± 11.55 nM), while only modest activity against A375 (754.69 ± 145.56 nM).

The A375 cell line presents the highest resistance to PDT among the tested cell lines. A375 cells are derived from a metastatic melanoma patient, present an HLA-A2 phenotype, and carry two mutant genes—B-Raf proto-oncogene (BRAF) and Cyclin-Dependent Kinase Inhibitor 2A (CDKN2) [70]. Scientific research shows differences in the susceptibility to photodynamic therapy between pigmented and unpigmented melanoma [71]. The presence of melanin that absorbs PDT light and has an antioxidant effect might decrease therapeutic outcomes [72]. However, based on literature data, A375 cells did not express MART-1/MelanA and Pmel17/gp100, which are involved in melanosome formation and melanin synthesis [73]; thus, A375 is considered an amelanotic phenotype. Therefore, the higher resistance to the tested compounds seems unrelated to the melanin content in the cells. On the other hand, melanoma cells have high reactive oxygen species (ROS) levels and a more robust antioxidant defense system than melanocytes. Scientific research indicates that melanoma cells have efficient mechanisms involved in antioxidant defense, such as the activation of the nuclear factor erythroid 2–related factor 2 (NRF2) and a higher level of glutathione (GSH) and nicotinamide adenine dinucleotide phosphate (NADPH) [74]. The formation of NADPH in cells is related to the pentose pathway. NADPH contributes to regenerating reduced glutathione and ROS production through NADPH oxidases catalyzing electron transfer from the NADPH molecule to molecular oxygen, thus maintaining the redox balance. Paudel et al. found that melanoma cells with reduced sensitivity to BRAF inhibitor exhibit an enhanced anti-oxidation and redox buffer capacity, specifically through NADPH oxidizing enzymes [74]. Thus, the lowest activity against A375 could be related to being more resistant to ROS generation. The A375 cell line showed the highest sensitivity to compound **4**, a free-base porphyrin substituted with a methoxy group in the *meta* position, with an IC_50_ value of 250.59 nM.

Interestingly, the zinc-containing derivatives showed different biological activity depending on the location of the methoxy group and the PEG substituent. A methoxy group at the phenyl *meta*-position and PEG at the *para*-position in compound **6** increased the photodynamic activity (IC_50_ values of 15.56 nM, 48.63 nM, and 284.56 nM for 5637, LNCaP, and A375 cells, respectively) compared to **5** with a methoxy group at the *para*-position and PEG at the *meta*-position (IC_50_ values of 79.71 nM, 106 nM, and 578.01 nM for 5637, LNCaP, and A375 cells, respectively). The position of both the methoxy and PEG functional groups showed a stronger relationship for the zinc derivatives than the cobalt-containing compounds and metal-free counterparts. It is well-known that the location of the zinc atom in the porphyrin ring increases the molecule’s stability; therefore, zinc is widely used in synthesizing new metalloporphyrins [75]. As reported by Pavlíčková et al., the incorporation of a zinc ion and PEGylation significantly increased the photodynamic activity of Purpurin 18. The incorporation of the PEG moiety in the structure of Purpurin 18 increased the cytotoxic activity and singlet oxygen generation [38]. Purpurin 18 exhibited the highest phototoxicity in the prostatic cancer cell lines with IC_50_ values of 160 nM and 340 nM for the PC-3 and LNCaP cells, respectively. The Purpurin 18 derivative containing a zinc ion without PEG3 spacers exerted a slightly lower cytotoxic activity, with IC_50_ values of 210 nM and 470 nM for PC-3 and LNCaP, respectively. Interestingly, PEGylation of Purpurin 18 decreased the IC_50_ value to 40 nM, while the PEGylated zinc-contained derivative reached an IC_50_ value of 20 nM in the LNCaP cells [38]. These findings showed that PEG incorporation and zinc insertion increase activity against LNCaP cells. We observed that the free bases **3** and **4** and zinc derivative **6** exerted a similar cytotoxic activity against LNCaP. This result suggested that metal insertion did not change activity against LNCaP cells. On the other hand, it seems that more important is the localization of the PEG chains in the phenyl ring, while compound **5** (with PEG chains at the *meta* position) exerted a lower activity than its analog, compound **6** (with PEG chains at the *para* position), against LNCaP cells. Králová et al. synthesized the series of porphyrins with monoethyleneglycol chains containing hydroxy and methoxy as terminal groups [76]. The authors showed that porphyrins with monoethyleneglycol functionalities in the *meta* position increased the PDT efficacy compared to a parental photosensitizer, tetrahydroxy-phenyl porphyrin (m-THPP). The IC_50_ values for the m-THPP and PEGylated derivatives in human promyelocytic leukemia (HL-60) cells were similar. However, the authors used different light doses to irradiate the free and PEGylated compounds (13.3 J/cm^2^ and 2.5 J/cm^2^, respectively). Therefore, the overall photodynamic dose (regarding the drug dose and light dose) is required to reach lower for *meta*-ethylene glycol-functionalized porphyrin than for m-THPP. The symmetrical *meta* derivative was needed at a 47-fold lower concentration to achieve an IC_50_ value in HL-60 cells compared to the derivative with glycol chains in the *para* position. To the contrary, our results showed that when comparing IC_50_ values between all tested cell lines, it seems that the *para* derivatives **4** and **6** exerted the most prominent cytotoxic activity, even against the more resistant A375 cells.

On the other hand, PEGylation might cause the opposite effect and decrease anticancer activity. As reported by Nawalany et al., PEGylated p-THPP exerted a lower photocytotoxic effect against human colorectal carcinoma (HCT-116) and human prostate carcinoma (DU-145) cell lines compared to free p-THPP after irradiation at a dose of 15 J/cm^2^ [39]. The free p-THHP had IC_50_ values of 0.8 µg/mL (which corresponds to 1.2 µM) and 2.4 µg/mL (which corresponds to 3.5 µM) in HCT-116 and DU-145 cells, respectively. Noteworthy, p-THPP also decreased the cell viability in dark conditions, with IC_50_ values of 5.4 µg/mL (which corresponds to ~8 µM) and 14 µg/mL (which corresponds to ~20 µM). At the same time, no cytotoxic effect was observed for PEGylated p-THPP without irradiation [39]. Thus, PEGylated derivatives might still exert photocytotoxic activity, while their dark toxicity can be diminished. Moreover, the authors did not observe the apparent relationship between phototoxicity and the length of the PEG chain for the porphyrin–PEG conjugates. Therefore, as presented in the literature, PEGylation might improve as well as decrease the activity compared to its non-PEGylated counterparts. Moreover, the localization of the PEG substituents might change the cytotoxic activity.

## 3. Materials and Methods

Reagents and solvents for synthesis were purchased from commercial suppliers (Merck (Kenilworth, NJ, USA), TCI (Tokyo, Japan), Alfa Aesar (Waltham, MA, USA), and Avantor (Radnor, PA, USA)). ESI mass spectrometry for aldehydes was performed with an Agilent 1200 (Agilent, Santa Clara, CA, USA) with an ESI-MS/MS 6410 B Triple Quad detector. Porphyrin MALDI-TOF mass spectrometry was performed with UltrafleXtreme (Bruker Daltonics, Billerica, MA, USA); the matrix used was α–cyano-4-hydroxy cinnamic acid and the laser power was a 100 µJ/pulse. NMR data were collected on a Bruker AVANCE II 400 or AVANCE III 500 spectrometer. UV-Vis spectra were recorded on a UV-Vis Jasco V-770 spectrophotometer (JASCO, Tokyo, Japan). Singlet oxygen measurement was performed on a FluoTime 300 fluorescence spectrophotometer (Pico-Quant, Berlin, Germany). The experiment was performed at an excitation wavelength of 409 nm. IR spectra were recorded on a Shimadzu IRAffinity-1 spectrometer (Shimadzu, Kioto, Japan) using KBr tablets. The IR spectra are included in the Supplementary Material in Figures S44–S49.

Reagents used for the in vitro experiments, Dulbecco’s Modified Eagle’s Medium (DMEM), fetal bovine serum (FBS), phosphate-buffered saline (PBS), trypsin-EDTA, L-glutamine (200 mM), penicillin (10,000 units), streptomycin (10 mg/mL) solution, dimethyl sulfoxide (DMSO), and 3-(4, 5-dimethylthiazol-2-yl)-2,5-diphenyltetrazolium bromide (MTT), were obtained from Sigma Aldrich (St. Louis, MO, USA). The Roswell Park Memorial Institute (RPMI) 1640 Medium was obtained from Gibco (Thermo Fisher Scientific, Waltham, MA, USA). The DMSO for dissolving the formazan crystals was obtained from Avantor Performance Materials (Gliwice, Poland).

### 3.1. Molecular Modeling

Molecular modeling experiments were performed using the Gaussian 09 program [77]. The global minima of the structures were determined with the restricted DFT with a correlation-exchange functional—PBE1PBE. Geometrical optimization was carried out with the basis set 6-31G(d,p), and vibrational analysis confirmed the correctness of the found global minima (no imaginary frequencies were observed). Electronic spectra simulations were performed according to TD-DFT, PBE1PBE functional, basis function 6-31+G(d,p), and a solvation model—the Polarizable Continuum Model (PCM) in an integral equation formalism variant (IEFPCM). Contours of the simulated spectra generated with the Gaussian broadening of the spectral lines method implemented in Chemcraft [78] software were calculated according to the formula I = I_n × exp − (ln2 × ((v − v_n)/λ)^2), where λ is the full width at half maximum (this parameter value was chosen as 12), and values I_n_ and ν_n_ correspond to the computed transition oscillator strengths and locations on the wavelength axis, respectively.

### 3.2. PEGylated Aldehyde Synthesis—General Procedure

Aldehyde PEGylation was performed using the standard nucleophilic substitution method. To a solution of the corresponding aldehyde (vanillin, isovanillin) in DMF, we added 1.1 equivalent of Br(CH_2_CH_2_O)CH_3_ and 1.1 equivalent of K_2_CO_3_. The reaction was then heated to 80 °C and stirred for 24 h. After that, the reaction was quenched with distilled water and the product was extracted with ethyl acetate and purified with column chromatography using ethyl acetate and silica gel.

3-methoxy-4-(1,4,7,10-tetraoxoundecyl)benzaldehyde (1). MS (ESI) [M+H]^+^ = 299.2 m/z. ^1^H NMR (500 MHz, DMSO-*d*_6_) δ 9.84 (s, 1H), 7.53 (dd, *J* = 8.2, 1.9 Hz, 1H), 7.39 (d, *J* = 1.8 Hz, 1H, H2), 7.18 (d, *J* = 8.3 Hz, 1H), 4.24–4.16 (m, 2H), 3.83 (s, 3H), 3.78 (dd, *J* = 5.2, 4.0 Hz, 2H), 3.59 (dd, *J* = 5.9, 3.5 Hz, 2H), 3.55–3.52 (m, 2H), 3.52–3.49 (m, 2H), 3.42 (dd, *J* = 5.7, 3.8 Hz, 2H), 3.23 (s, 3H). Signals annotations shown in Figure 3. ^13^C NMR (126 MHz, DMSO-*d*_6_) δ 191.36, 153.43, 149.22, 129.70, 125.96, 112.17, 109.68, 71.26, 69.97, 69.78, 69.59, 68.68, 68.12, 58.01, 55.49. Signals annotations shown in Figure 3. Rf (ethyl acetate): 0.52, yield 79.2%.

4-methoxy-3-(1,4,7,10-tetraoxoundecyl)benzaldehyde (2). MS (ESI) [M+H]^+^ = 299.2 m/z, ^1^H NMR (500 MHz, DMSO-*d*_6_) δ 9.83 (s, 1H, CHO), 7.56 (dd, *J* = 8.2, 1.9 Hz, 1H, H6), 7.41 (d, *J* = 1.8 Hz, 1H, H2), 7.18 (d, *J* = 8.3 Hz, 1H, H5), 4.17–4.13 (m, 2H, 2′), 3.87 (s, 3H, C4-OCH3), 3.76 (dd, *J* = 5.2, 4.0 Hz, 2H, 3′), 3.59 (dd, *J* = 5.9, 3.5 Hz, 2H, 5′), 3.55–3.52 (m, 2H, 6′), 3.52–3.50 (m, 2H, 8′), 3.42 (dd, *J* = 5.7, 3.8 Hz, 2H, 9′), 3.23 (s, 3H, 11′). ^13^C NMR (126 MHz, DMSO-*d*_6_) δ 191.32, 154.32, 148.32, 129.59, 126.00, 111.48, 110.89, 71.24, 69.93, 69.77, 69.58, 68.79, 67.89, 58.00, 55.85. Rf (ethyl acetate): 0.43, yield 83.6%.

### 3.3. PEGylated Porphyrin Synthesis—General Procedure

Free porphyrins were synthesized according to the modified Adler–Longo procedure [43]. An equimolar mixture of pyrrole and PEGylated aldehyde in propionic acid was heated to 140 °C over 24 h. Then, after cooling, the reaction was poured into distilled water, and the acid was neutralized with a saturated Na_2_CO_3_ solution. The resulting precipitate was filtered and purified with column chromatography using silica gel.

5,10,15,20-tetra [4-methoxy-3-(1,4,7,10-tetraoxoundecyl)phenyl]porphyrin (3). MS (MALDI-TOF) [M+H]^+^ 1383.6589 m/z. ^1^H NMR (500 MHz, CDCl_3_) δ 8.90 (s, 8H), 7.84 (s, 4H), 7.77 (d, *J* = 5.5 Hz, 4H), 7.27 (s, 4H), 4.35 (t, *J* = 4.9 Hz, 8H), 4.16 (s, 12H), 3.96 (t, *J* = 4.7 Hz, 8H), 3.75 (s, 8H), 3.62 (s, 8H), 3.49 (d, *J* = 3.3 Hz, 8H), 3.32 (s, 8H), 3.20 (d, *J* = 1.8 Hz, 12H), −2.75 (s, 2H). Signals annotations shown in Figure 3.

^13^C NMR (126 MHz, CDCl_3_) δ 149.41, 146.43, 134.72, 130.94, 127.91, 120.51, 119.72, 110.00, 71.70, 70.79, 70.54, 70.36, 69.70, 68.73, 58.79, 56.18. Signals annotations shown in Figure 3.

UV-Vis (DMF) λ_max_ [nm] (log ε): 425 (4.49), 518 (3.21), 555 (2.91), 593 (2.57), 650 (2.47). IR ν [cm^−1^]: 3318, 2872, 1601, 1582, 1506, 1472, 1414, 1346, 1319, 1256, 1233, 1172, 1140, 1024, 976, 945, 897, 856, 799, 734, 617. Column chromatography—first ethyl acetate:acetone 2:1 to remove impurities then acetone:ethyl acetate 2:1 to elute the porphyrin. Rf (acetone:ethyl acetate 2:1) 0.63, yield 33.7%.

5,10,15,20-tetra [3-methoxy-4-(1,4,7,10-tetraoxoundecyl)phenyl]porphyrin (4)—MALDI-TOF[M-H]^+^ 1381.6303 m/z. ^1^H NMR (800 MHz, CDCl_3_) δ 8.91 (s, 8H), 7.78 (s, 4H), 7.73 (dd, *J* = 5.1, 2.5 Hz, 4H), 7.29 (d, *J* = 7.9 Hz, 4H), 4.50 (t, *J* = 5.1 Hz, 8H), 4.12–4.09 (m, 8H), 3.97 (d, *J* = 4.6 Hz, 12H), 3.90 (dd, *J* = 5.7, 4.1 Hz, 8H), 3.80 (dd, *J* = 5.7, 4.1 Hz, 8H), 3.76–3.73 (m, 8H), 3.63–3.60 (m, 8H), 3.42 (s, 12H), −2.74 (s, 2H). Signals annotations shown in Figure 3. ^13^C NMR (201 MHz, CDCl_3_) δ 148.37, 147.77, 135.42, 127.51, 119.97, 118.91, 111.85, 72.15, 71.13, 70.91, 70.78, 70.00, 68.90, 59.22, 56.30. Signals annotations shown in Figure 3.

UV-Vis (DMF) λ_max_ [nm] (log ε): 425 (4.59), 518 (3.13), 555 (2.83), 591 (2.39), 648 (1.79). IR ν [cm^−1^]: 3318, 2926, 1582, 1558, 1506, 1472, 1456, 1408, 1350, 1319, 1254, 1231, 1140, 1036, 974, 914, 860, 804, 740, 626. Column chromatography—first chloroform:acetone 1:1 to remove impurities then ethyl acetate:acetone 2:1 to elute the porphyrin Rf(ethyl acetate:acetone 2:1) 0.28, yield 27.8%.

### 3.4. Metallated PEGylated Porphyrins—General Procedure

Metallated porphyrins were synthesized by the reaction of the free porphyrin and an equimolar amount of the corresponding metal salt (zinc acetate or cobalt(II) chloride) in DMF at 80 °C for 24 h, and then evaporated under vacuum. Then, they were purified using column chromatography on silica gel with acetone:ethyl acetate 2:1 or dichloromethane:methanol 10:1 as the mobile phase.

5,10,15,20-tetra [4-methoxy-3-(1,4,7,10-tetraoxoundecyl)phenyl]porphyrin zinc(II) (5)-MALDI-TOF[M]^+^ 1444.6373 m/z. ^1^H NMR (400 MHz, CDCl_3_) δ 8.95 (s, 8H), 7.88 (s, 4H), 7.81–7.74 (m, 4H), 7.24 (dd, *J* = 7.0, 4.2 Hz, 4H), 4.35 (s, 8H), 4.15 (s, 12H), 3.89 (dt, *J* = 14.6, 7.3 Hz, 8H), 3.68–3.55 (m, 8H), 3.49–3.32 (m, 8H), 3.06–2.89 (m, 8H), 2.83–2.60 (m, 12H), 2.52 (dd, *J* = 22.2, 17.4 Hz, 8H). ^13^C NMR (101 MHz, CDCl_3_) δ 150.27, 149.12, 146.27, 135.73, 131.76, 127.63, 127.57, 121.01, 120.49, 109.76, 109.73, 71.01, 70.97, 70.88, 70.75, 70.44, 70.42, 69.98, 69.82, 69.74, 68.93, 58.25, 58.16, 58.01, 56.19. UV-Vis (DMF) λ_max_ [nm] (log ε): 426 (4.64), 554 (3.29), 596 (2.92). IR ν [cm^−1^]: 2930, 2872, 1600, 1578, 1558, 1506, 1456, 1408, 1338, 1256, 1207, 1170, 1140, 1024, 999, 957, 795, 772, 719, 615. Rf (acetone:ethyl acetate 2:1) 0.72, yield 93.3%.

5,10,15,20-tetra [3-methoxy-4-(1,4,7,10-tetraoxoundecyl)phenyl]porphyrin zinc(II) (6)–MALDI-TOF[M-OCH_3_]^+^ 1416.6075 m/z. ^1^H NMR (500 MHz, CDCl_3_) δ 8.99 (s, 8H), 7.80–7.75 (m, 4H), 7.72 (d, *J* = 7.9 Hz, 1H), 7.22 (s, 4H), 4.36 (d, *J* = 5.3 Hz, 8H), 3.99–3.88 (m, 20H), 3.73 (s, 8H), 3.66–3.54 (m, 16H), 3.45 (s, 8H), 3.32–3.24 (m, 12H). 13C NMR (126 MHz, CDCl_3_) δ 150.38, 147.94, 147.41, 136.02, 131.87, 127.21, 120.71, 118.67, 111.52, 71.80, 70.78, 70.76, 70.56, 70.54, 70.43, 70.41, 69.70, 69.68, 68.57, 58.91, 56.08, 56.07. UV-Vis (DMF) λ_max_ [nm] (log ε): 425 (4.78), 551 (3.44), 592 (2.67). IR ν [cm^−1^]: 2928, 2874, 1601, 1578, 1558, 1506, 1456, 1408, 1339, 1317, 1258, 1238, 1209, 1170, 1136, 1062, 1024, 999, 957, 797, 772, 719, 617. Rf (acetone:ethyl acetate 2:1) 0.69, yield 87.6%.

5,10,15,20-tetra [4-methoxy-3-(1,4,7,10-tetraoxoundecyl)-phenyl] porphyrin cobalt(II) (7)–MALDI-TOF [M]^+^ 1439.5713 m/z. IR ν [cm^−1^]: 2930, 2872, 1601, 1578, 1558, 1506, 1456, 1410, 1350, 1318, 1300, 1211, 1172, 1136, 1024, 960, 796, 771, 710, 619. UV-Vis (DMF) λ_max_ [nm] (log ε): 429 (5.13), 547 (4.03), 589 (3.66); Rf (dichloromethane: methanol 10:1) 0.4, yield 62.8%.

5,10,15,20-tetra [3-methoxy-4-(1,4,7,10-tetraoxoundecyl)-phenyl] porphyrin cobalt(II) (8)–MALDI-TOF[M]^+^ 1439.5794 m/z. IR ν [cm^−1^]: 2924, 2872, 1601, 1578, 1558, 1510, 1456, 1406, 1350, 1318, 1259, 1211, 1172, 1136, 1028, 1006, 934, 868, 796, 775, 712, 626. UV-Vis (DMF) λ_max_ [nm] (log ε): 430 (5.06), 547 (3.97), 586 (3.60); Rf (dichloromethane:methanol 10:1) 0.37, yield 56.2%.

### 3.5. Singlet Oxygen Measurement

Tested porphyrins and TmPyP, the reference compound, were dissolved in dry DMF and diluted until ca. 0.1 absorbance. They were then transferred to a quartz cuvette. The measurement was conducted on a FluoTime 300 fluorescence spectrophotometer (Pico-Quant) with an excitation wavelength of 409 nm. Singlet oxygen quantum yields were calculated with TmPyP as reference (Φ_Δ_ = 0.73) using the following equation: $\Phi_{\Delta }PS=\Phi_{\Delta }R\left(\frac{1-10^{-Abs\;R}}{1-10^{-Abs\;PS}}\right)\left(\frac{S_{e}^{PS}}{S_{e}^{R}}\right)$, where PS is the tested photosensitizer, R is the reference, Abs is the absorbance, and S_e_ is the singlet oxygen phosphorescence signal [79,80].

### 3.6. Chromatographic Analysis of Purity

The chromatographic analysis was performed on an Agilent 1260 Infinity II LC System (Agilent Technologies, Bolinem, Germany) equipped with a quaternary pump (model G7111B) and degasser, a vial sampler (model G7129A) set at 20 °C, multicolumn thermostat (model G7116A) set at 35 °C, and two detectors—a diode array (DAD WR, model G7115A) and evaporative light scattering (ELSD, model G4260B). The detection wavelength was adjusted for each tested compound at their absorption maxima. The column used as stationary phase (C-18(2) 100 Å Luna^®^, 150 × 4.6 mm ID, 5 µm, Phenomenex, Torrance, CA, USA) and gradient solvent systems of H_2_O (phase A) and acetonitrile (phase B) used as the mobile phase at a flow rate of 1.0 mL/min. A sample volume of 10 µL was injected onto the column. The sample was prepared by dissolution of the compound in acetonitrile. All chromatograms showed a single main peak between 14.94 min and 17.71 min, with purity from 95% to 100%. Detailed data can be found in the Supplementary Material.

### 3.7. Cytotoxic Activity of the Tested Compounds

Human grade II bladder carcinoma 5637 cell line and the human prostate carcinoma LNCaP cell line were obtained from the American Type Culture Collection (ATCC; Manassas, VA, USA), while the human malignant melanoma A375 cell line was purchased from the European Collection of Authenticated Cell cultures (ECACC, Salisbury, UK). LNCaP and A375 cells were maintained in DMEM, while 5637 cells were cultured in the RPMI medium. Media were supplemented with 10% fetal bovine serum (FBS), 1% (*v*/*v*) antibiotics, and 1% (*v*/*v*) L-glutamine solution (final concentration 2 mM). All cell culture media were phenol-red-free. Cells were cultured at 37 °C in 5% CO_2_ and 95% relative humidity.

Stock solutions (1mM) were prepared by dissolving compounds in DMSO and stored in the dark at −20 °C. The stock solutions were diluted to the final working concentrations in cell culture media. The stock solutions were clear and free of undissolved compounds (checked by both centrifugations and microscopic examination). The final concentration of DMSO in the experiments did not exceed 0.1% in the cell culture medium. The dark- and light-dependent cytotoxicity were determined for each photosensitizer.

The cytotoxic effect of the tested formulations was determined using the MTT assay [81]. The 5637, A375, and LNCaP cells were seeded at a density of 15 × 10^3^ cells/well in 96-well plates and incubated overnight. The 5637 cells were treated with PSs at concentrations of 7, 15, 30, 60, 125, and 250 nM for compounds **3**, **4**, and **6**; and 30, 60, 125, 250, 500, and 1000 nM for compounds **5**, **7**, and **8**. The LNCaP cells were treated with PSs at concentrations of 7, 15, 30, 60, 125, and 250 nM for compounds **3** and **6**; and at concentrations of 30, 60, 125, 250, 500, and 1000 nM for compounds **4**, **5**, **7**, and **8**. The A375 cells were treated with PSs at concentrations of 30, 60, 125, 250, 500, and 1000 nM. After 24 h, the cells were washed twice with PBS, and a fresh medium was added to each well. Then, the cells were irradiated at a light dose of 10 J/cm^2^ using a lamp emitting light at a wavelength of 425 nm or not irradiated in the case of dark control plates. The laser radiation power density was approximately 25 mW/cm^2^. A radiometer device PM16-130 Power Meter with Slim Photodiode Sensor (ThorLabs, Newton, NJ, USA) was used to measure the illumination power before each experiment. Cytotoxic activity was measured by using the MTT assay 24 h after irradiation. The MTT solution (5 mg/mL PBS) in cell culture medium (final concentration of 0.59 mg/mL) was then added to each well and incubated for 1.5 h under standard cell culture conditions. The formazan crystals were dissolved in 200 μL DMSO, and the absorbance was measured at 570 nm with a plate reader (Biotek Instruments, Elx-800, Winooski, VT, USA). Cell viability was calculated as a percentage of the control. All experiments were repeated at least three times (three independent experiments performed in hexaplicates). The IC_50_ values were determined using GraphPad 8.0 software (GraphPad Software, Inc., La Jolla, CA, USA).

## 4. Conclusions

This work presents the synthesis, photochemical properties, and biological activity of novel photosensitizers containing short PEG chains. The proposed strategy might improve the solubility and cellular uptake while not diminishing efficacy due to the enormous PEG pendants usually employed in this modification. We have successfully used a simple synthetic method to obtain the porphyrins with nanomolar photodynamic activity against cancer cells. Tested compounds have strong absorption in the 420–435 nm range with a high molar extinction coefficient. The computational model used in predicting the UV-Vis spectra gave good agreement with the experimental data. It also confirmed the assumptions of the Gouterman model of the greatest influence of the four molecular orbitals (HOMO-1, HOMO, LUMO, and LUMO+1) on the formation of the Q bands and the Soret bands. Singlet oxygen quantum yield was highest for free-base porphyrins and lowest for the cobalt complexes. In vitro experiments showed that all the tested compounds did not exert dark toxicity. Interestingly, different susceptibility to the tested compounds was observed across cell lines. The metal-free compound **3** showed the strongest cytotoxic effect against 5637 cells, with an IC_50_ value of 8 nM. In contrast, this compound exerted 6- and 94-fold lower activity against LNCaP and A375 cells. Prostate cancer LNCaP cells showed the highest sensitivity to the three tested compounds, **3**, **4**, and **6**, with IC_50_ values of 49 nM, 44 nM, and 48 nM, respectively. The most resistant to treatment was melanoma A375 cells. Interestingly, the cobalt-based compounds were completely inactive against A375 cells. The activity of the compounds against specific cell lines strictly depended on their chemical structure, PEG group location in the phenyl ring, and the type of metal atom coordinated in the main ring. Further studies are required to examine the underlying mechanisms, particularly considering the localization of PSs, photokilling mode of action, and cell-type-specific responses to PDT.

## Figures and Tables

**Table 1:** The essential vertical excitations of **3** at the PCM/TD-DFT/PBE1PBE/6-31+G(d,p)//PBE1PBE/6-31G(d,p) of the theory.

Experimental Band Maximum (nm)	Simmulated Band Maximum (nm)	Transition Number	λ (nm)	EE ^1^ (eV)	OS ^2^ (f)	Orbital Transition Composition ^3^ (% of Contribution)	Type	Band ^4^
597	592	1	591.757	2.095	0.106	HOMO → LUMO (73), HOMO-1 → LUMO+1 (24)	π-π*	Q
564	557	2	557.636	2.223	0.157	HOMO → LUMO+1 (73), HOMO-1 → LUMO (25)	π-π*	Q
426	428	3	430.757	2.878	1.023	HOMO-1 → LUMO+1 (43), HOMO-2 → LUMO (21)	π-π*	B
426	428	4	426.167	2.909	1.061	HOMO-1 → LUMO (37), HOMO-2 → LUMO (26)	π-π*	B

^1^ Excitation Energy; ^2^ Oscillator strength; ^3^ Only transitions with contribution above 20%; ^4^ Band type: Q—Q Band; B—Soret Band.

**Table 2:** The essential vertical excitations of **4** at the PCM/TD-DFT/PBE1PBE/6-31+G(d,p)//PBE1PBE/6-31G(d,p) of the theory.

Experimental Band Maximum (nm)	Simmulated Band Maximum (nm)	Transition Number	λ (nm)	EE ^1^ (eV)	OS ^2^ (f)	Orbital Transition Composition ^3^ (% of Contribution)	Type	Band ^4^
597	597	1	596.454	2.079	0.150	HOMO → LUMO (76), HOMO-1 → LUMO+1 (22)	π-π*	Q
564	564	2	563.773	2.199	0.227	HOMO → LUMO+1 (77), HOMO-1 → LUMO (22)	π-π*	Q
426	426	3	428.806	2.891	1.177	HOMO-1 → LUMO+1 (48)	π-π*	B
426	426	4	423.431	2.928	1.273	HOMO-1 → LUMO (48)	π-π*	B

^1^ Excitation Energy; ^2^ Oscillator strength; ^3^ Only transitions with contribution above 20%; ^4^ Band type: Q—Q Band; B—Soret Band.

**Table 3:** The essential vertical excitations of **5** at the PCM/TD-DFT/PBE1PBE/6-31+G(d,p)//PBE1PBE/6-31G(d,p) of theory.

Experimental Band Maximum (nm)	Simmulated Band Maximum (nm)	Transition Number	λ (nm)	EE ^1^ (eV)	OS ^2^ (f)	Orbital Transition Composition ^3^ (% of Contribution)	Type	Band ^4^
561	563	1	563.030	2.202	0.139	HOMO → LUMO (71), HOMO-1 → LUMO+1 (24]	π-π*	Q
561	563	2	563.030	2.202	0.139	HOMO → LUMO+1 (71), HOMO-1 → LUMO (24)	π-π*	Q
431	420	3	419.747	2.954	1.429	HOMO-1 → LUMO+1 (38) HOMO-1 → LUMO (26)	π-π*	B
431	420	4	419.747	2.954	1.429	HOMO-1 → LUMO (38), HOMO-1 → LUMO+1 (26)	π-π*	B

^1^ Excitation Energy; ^2^ Oscillator strength; ^3^ Only transitions with contribution above 20%; ^4^ Band type: Q—Q Band, B—Soret Band.

**Table 4:** Singlet oxygen quantum yield of synthesized porphyrins.

	3	4	5	6	7	8
Φ_Δ_	0.54	0.53	0.43	0.4	0.06	0.05

**Table 5:** The IC_50_ values of **3**, **4**, **5**, **6**, **7**, **8** against 5637, LNCaP, and A375 cells. Data are expressed as the mean ± SD from at least three independent experiments.

Compound	IC_50_ [nM]					
	5637		LNCaP			A375
	0 J/cm^2^	10 J/cm^2^	0 J/cm^2^	10 J/cm^2^	0 J/cm^2^	10 J/cm^2^
**3**	>250	8.01 ± 2.12	>250	49.71 ± 11.55	>1000	754.69 ± 145.56
**4**	>250	32.13 ± 3.86	>1000	44.91 ± 1.98	>1000	250.59 ± 64.51
**5**	>1000	79.71 ± 7.08	>1000	106.00 ± 26.27	>1000	578.01 ± 25.44
**6**	>250	15.56 ± 3.50	>250	48.63 ± 13.21	>1000	284.56 ± 83.21
**7**	>1000	149.92 ± 42.12	>1000	557.75 ± 104.99	>1000	>1000
**8**	>1000	247.94 ± 45.45	>1000	333.53 ± 116.33	>1000	>1000

## Data Availability

Not applicable.

## Supplementary Materials

The following supporting information can be downloaded at: https://www.mdpi.com/article/10.3390/ijms231710029/s1.
