# Supplementary material for: Novel Short PEG Chain-Substituted Porphyrins: Synthesis, Photochemistry, and In Vitro Photodynamic Activity against Cancer Cells
Source: Int J Mol Sci. 2022 Sep 2;23(17):10029. doi: 10.3390/ijms231710029 (PMC9456001; doi:10.3390/ijms231710029)
Supplement: Supplementary file 1 [file ijms-23-10029-s001.zip › ijms-1837827-supplementary.pdf]

Novel Short PEG Chains Substituted Porphyrins: Synthesis, Photochemistry and In Vitro Photodynamic Activity Against Cancer Cells

Dawid Lazewski[1], Malgorzata Kucinska[2], Edward Potapskiy[1], Joanna Kuzminska[3], Artur Tezyk[4], Lukasz Popenda[5], Stefan Jurga[5], Anna Teubert[6], Zofia Gdaniec[6], Jacek Kujawski[7], Katarzyna Grzyb[8], Tomasz Pedzinski[8,9], Marek Murias[2,9], Marcin Wierzchowski[1]

[1] Department of Chemical Technology of Drugs, Poznan University of Medical Sciences, Grunwaldzka 6 Street, 60-780 Poznan, Poland

[2] Department of Toxicology, Poznan University of Medical Sciences, Dojazd 30 Street, 60-631 Poznan, Poland

[3] Department of Pharmaceutical Chemistry, Poznan University of Medical Sciences, Grunwaldzka 6 Street, 60-780 Poznan, Poland

[4] Department of Forensic Medicine, Poznan University of Medical Sciences, Swiecickiego 6 Street, 60-781 Poznan, Poland

[5] NanoBioMedical Centre, Adam Mickiewicz University in Poznan, Wszechnicy Piastowskiej 3 Street, 61-614 Poznan, Poland

[6] Institute of Bioorganic Chemistry, Polish Academy of Sciences, Noskowskiego 12/14 Street, 61-704 Poznan, Poland

[7] Department of Organic Chemistry, Poznan University of Medical Sciences, Grunwaldzka 6 Street, 60-780 Poznan, Poland

[8] Faculty of Chemistry, Adam Mickiewicz University in Poznan, Uniwersytetu Poznanskiego 8 Street, 61-614 Poznan

[9] Center for Advanced Technology, Adam Mickiewicz University in Poznan, Uniwersytetu Poznanskiego 10 Street, 61-614 Poznan

*Correspondence: marek.murias@ump.edu.pl (M.M.); mwierzch@ump.edu.pl (M.W.)

Supporting Information

Table of contents

1. NMR data 3
   1.1. NMR experiments of compound **1**. 3
   1.2. NMR experiments of compound **2**. 6
   1.3. NMR experiments of compound **3**. 9
   1.4. NMR experiments of compound **4**. 13
   1.5. NMR experiments of compound **5**. 17
   1.6. NMR experiments of compound 6. 21
2. Mass spectrometry data 25
   1.7. ESI experiments 25
   1.8. Data from MALDI-TOF experiments 26
3. IR data 32
4. UV-vis data 35
4 HPLC data 37

6 Computational data 38
The first twenty vertical excitations of **3** at the PCM/TD-DFT/PBE1PBE/6-31+G(d,p)//PBE1PBE/6-31G(d,p) of theory. 38
The first twenty vertical excitations of **4** at the PCM/TD-DFT/PBE1PBE/6-31+G(d,p)//PBE1PBE/6-31G(d,p) of theory. 40
The first twenty vertical excitations of **5** at the PCM/TD-DFT/PBE1PBE/6-31+G(d,p)//PBE1PBE/6-31G(d,p) of theory. 42
Atomic coordinates of global minimum of compound 3........ 43
Atomic coordinates of global minimum of compound 4........ 49
Atomic coordinates of global minimum of compound 5........ 55

1. NMR data

1.1. NMR experiments of compound **1**.

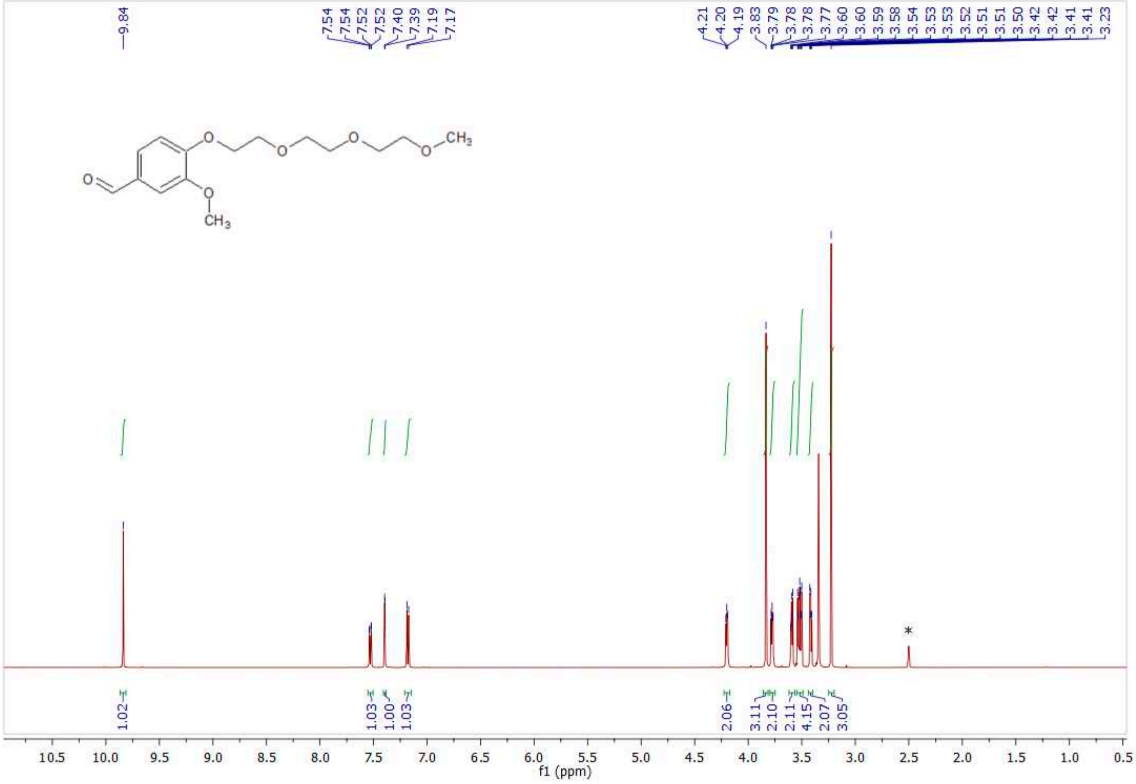

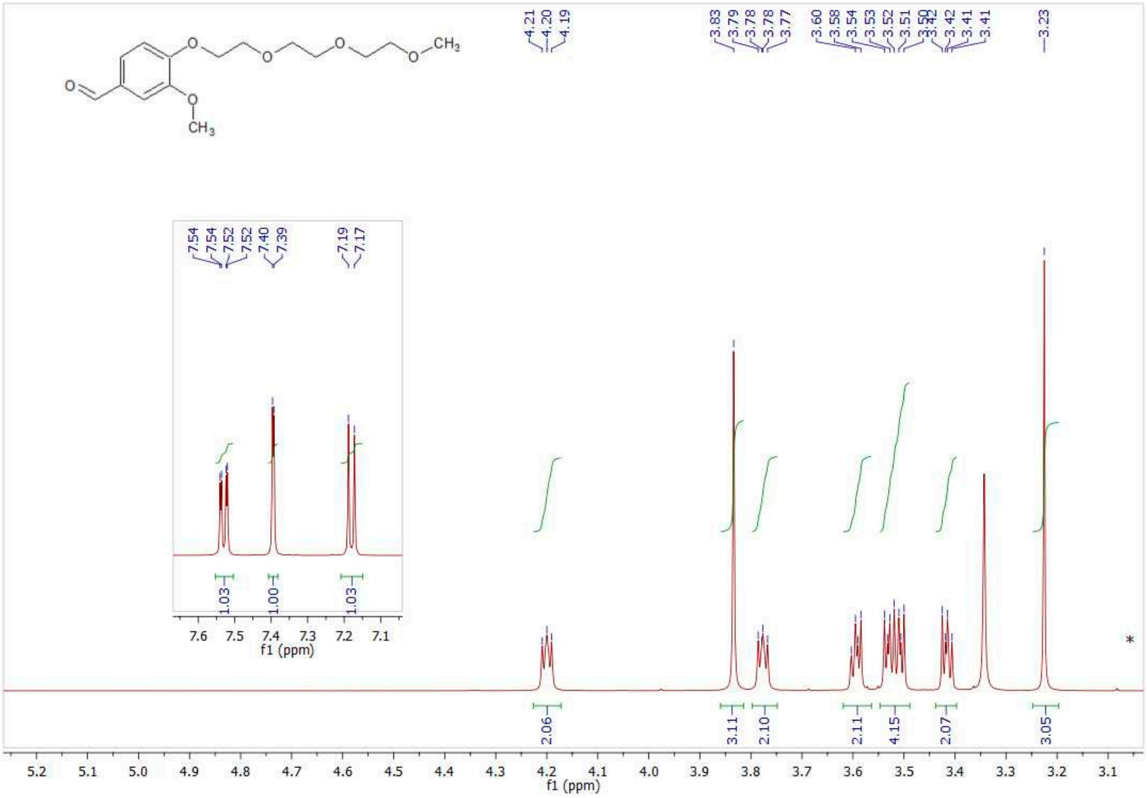

Figure S1. ^{1}H spectra NMR of aldehyde **1**.

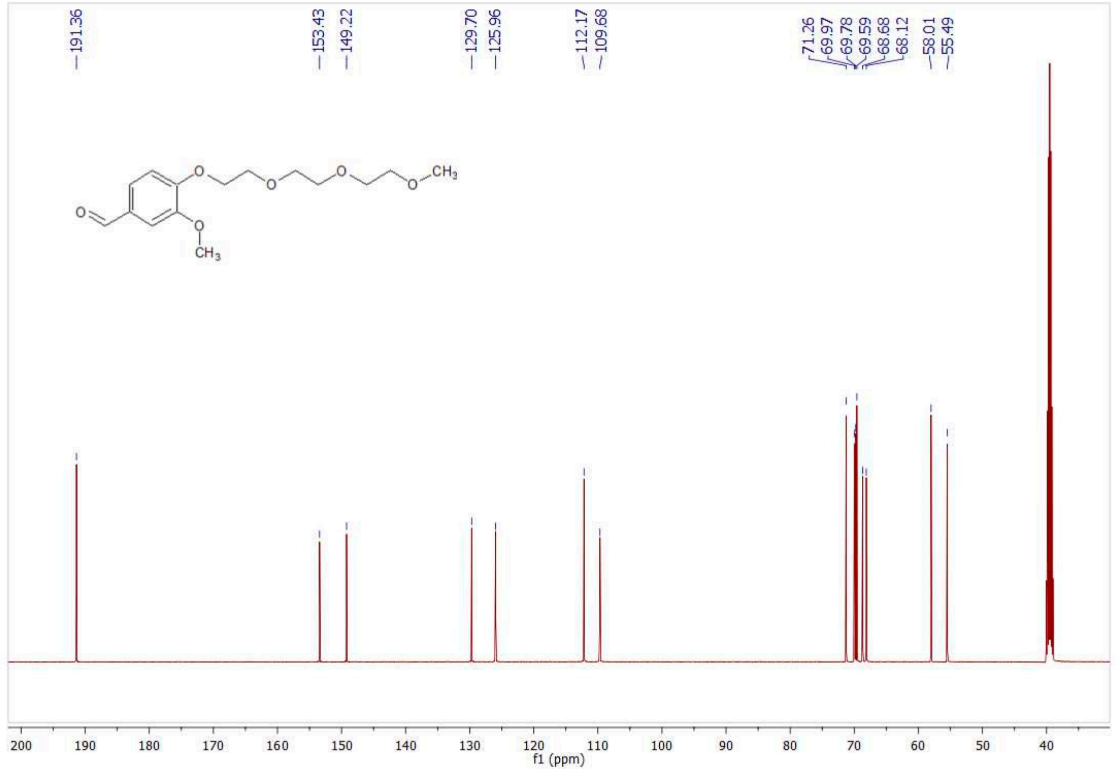

Figure S2. ^{13}C NMR spectrum of aldehyde **1**.

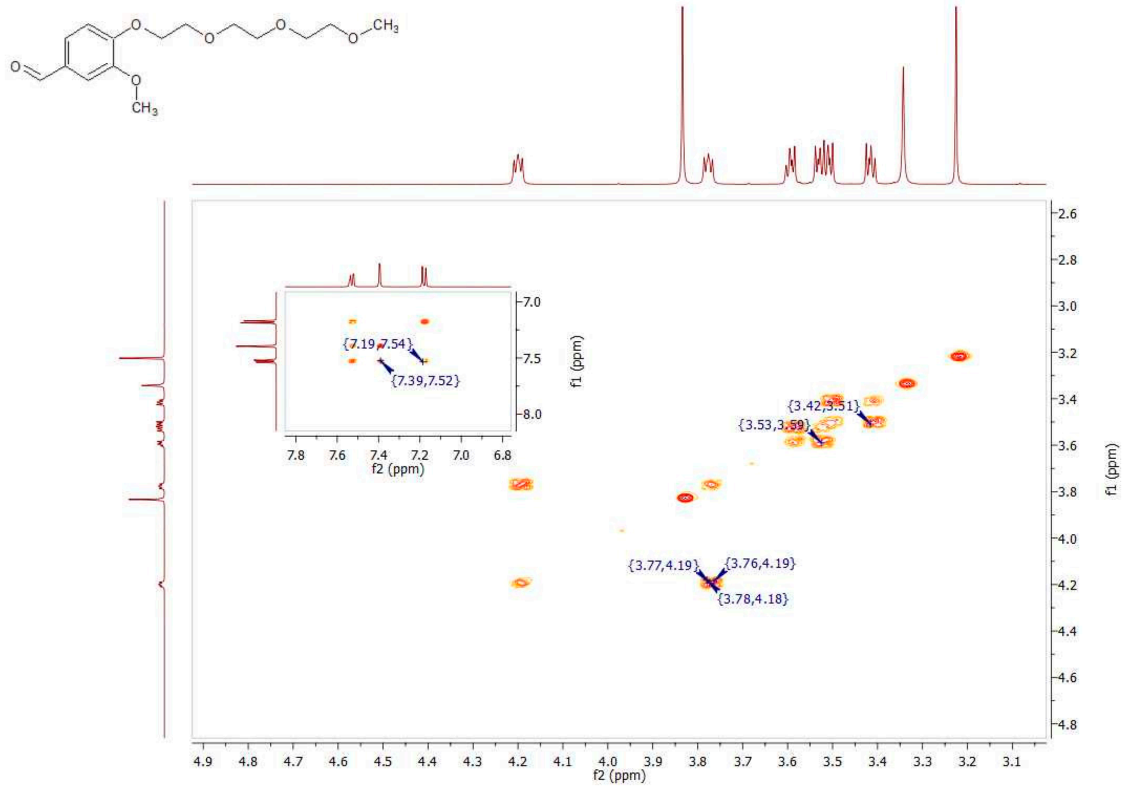

Figure S3. ^{1}H-^{1}H COSY spectrum of aldehyde **1**.

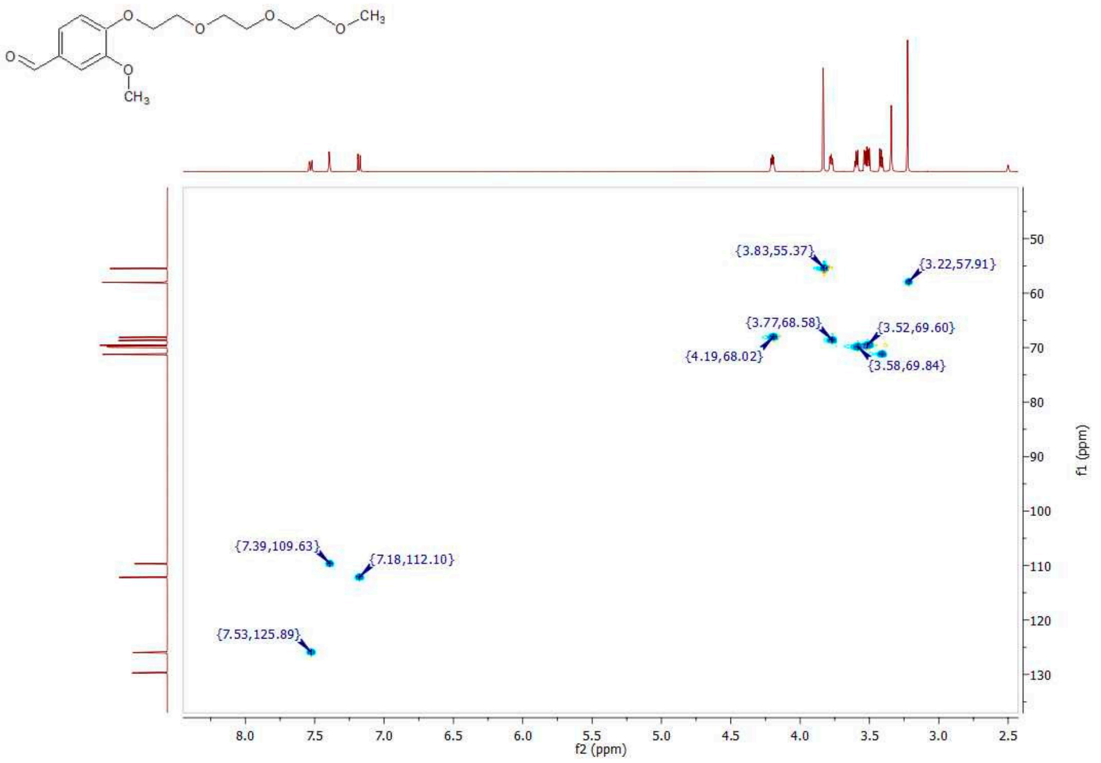

Figure S4. ^{1}H-^{13}C HSQC spectrum of aldehyde **1**.

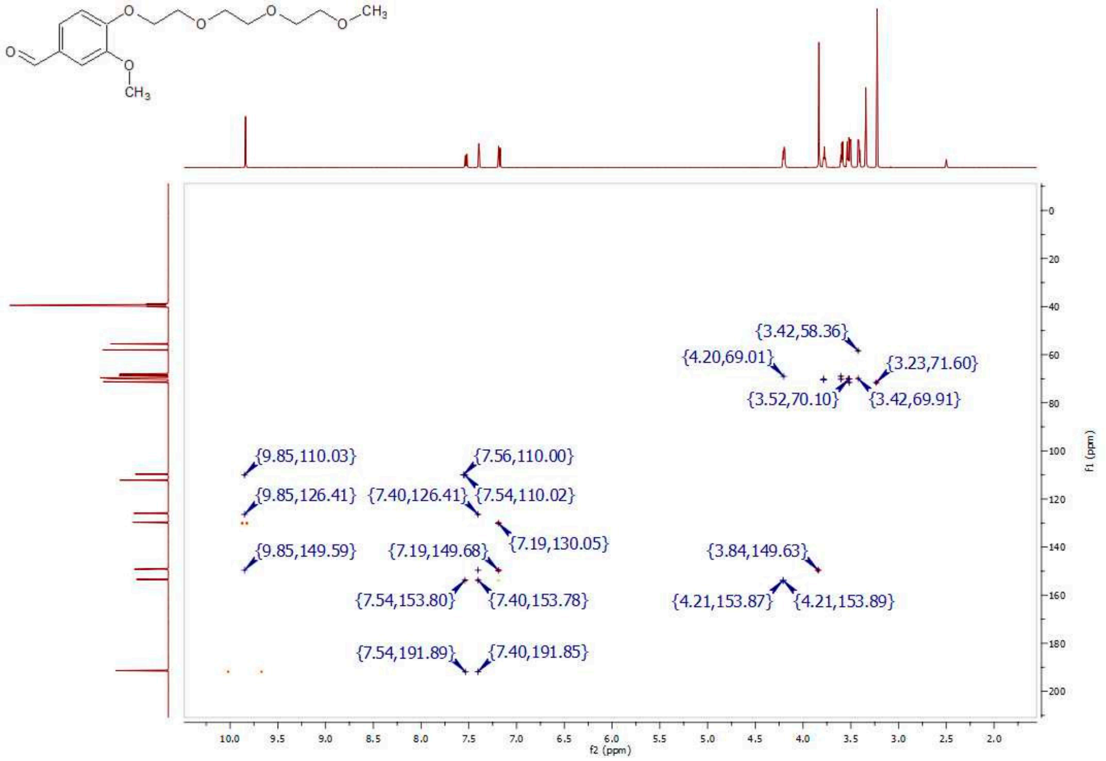

Figure S5. ^{1}H-^{13}C HMBC spectrum of aldehyde **1**.

1.2. NMR experiments of compound **2**.

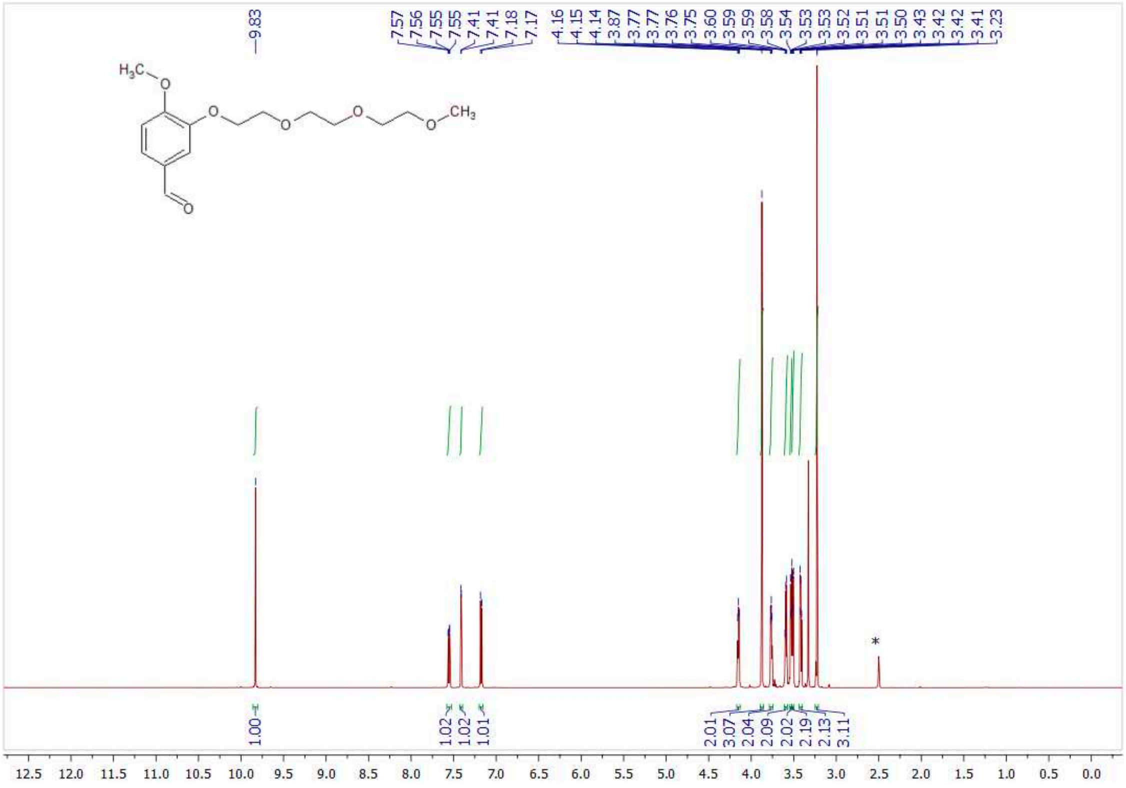

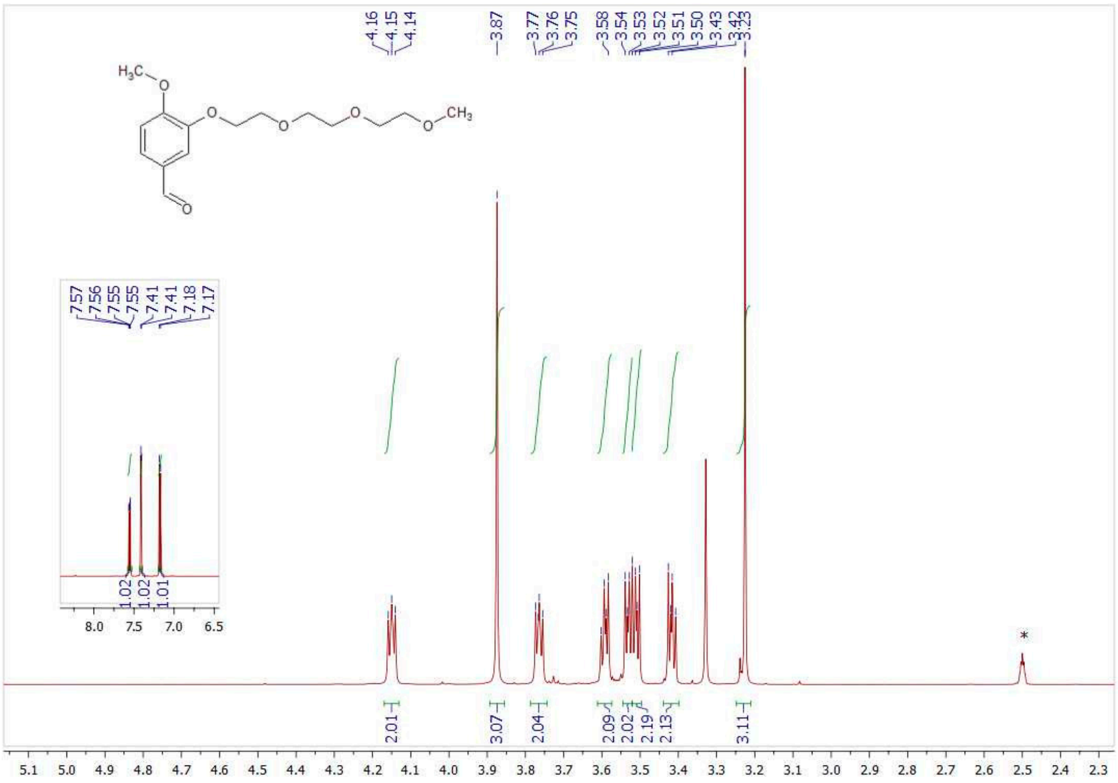

Figure S6. ^{1}H NMR spectra of aldehyde **2**.

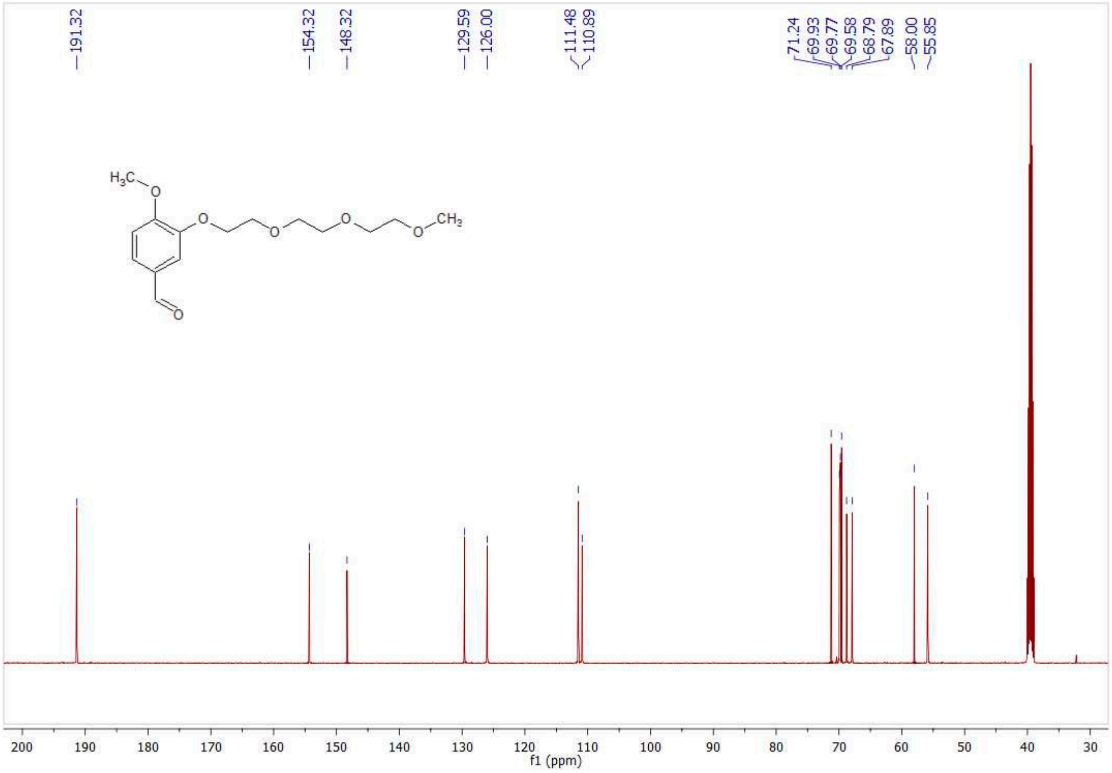

Figure S7. ^{13}C spectrum NMR of aldehyde **2**.

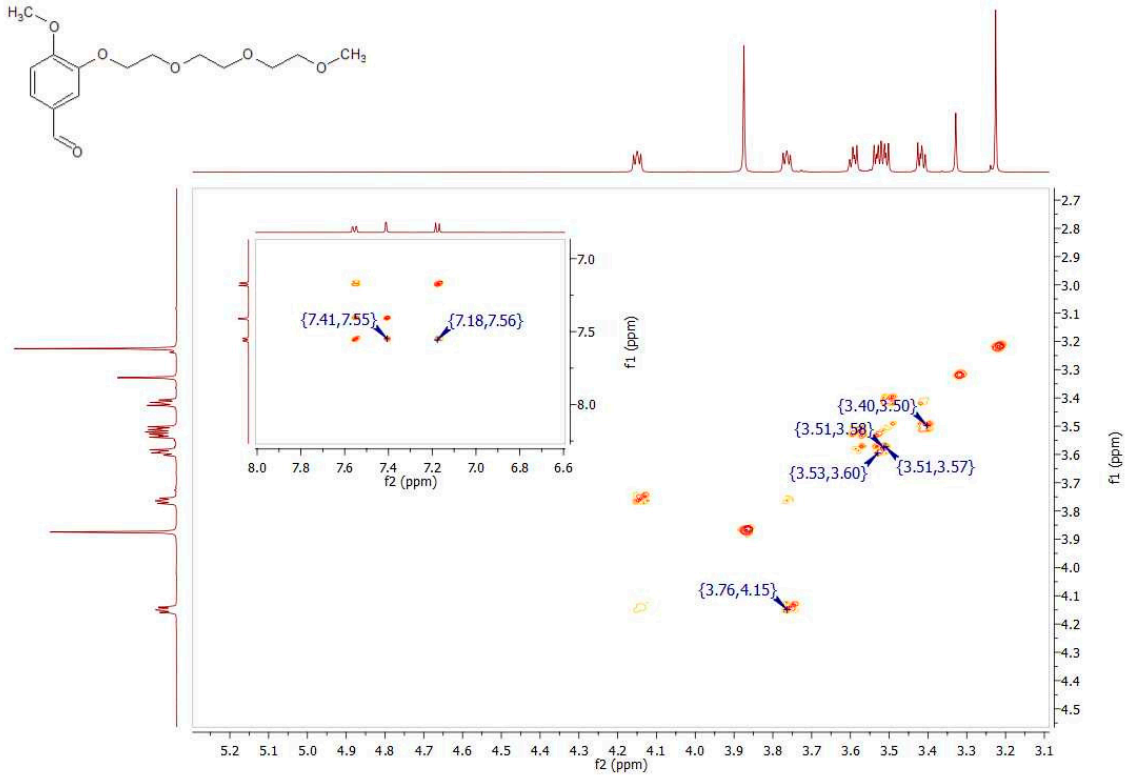

Figure S8. ^{1}H-^{1}H COSY spectrum of aldehyde **2**.

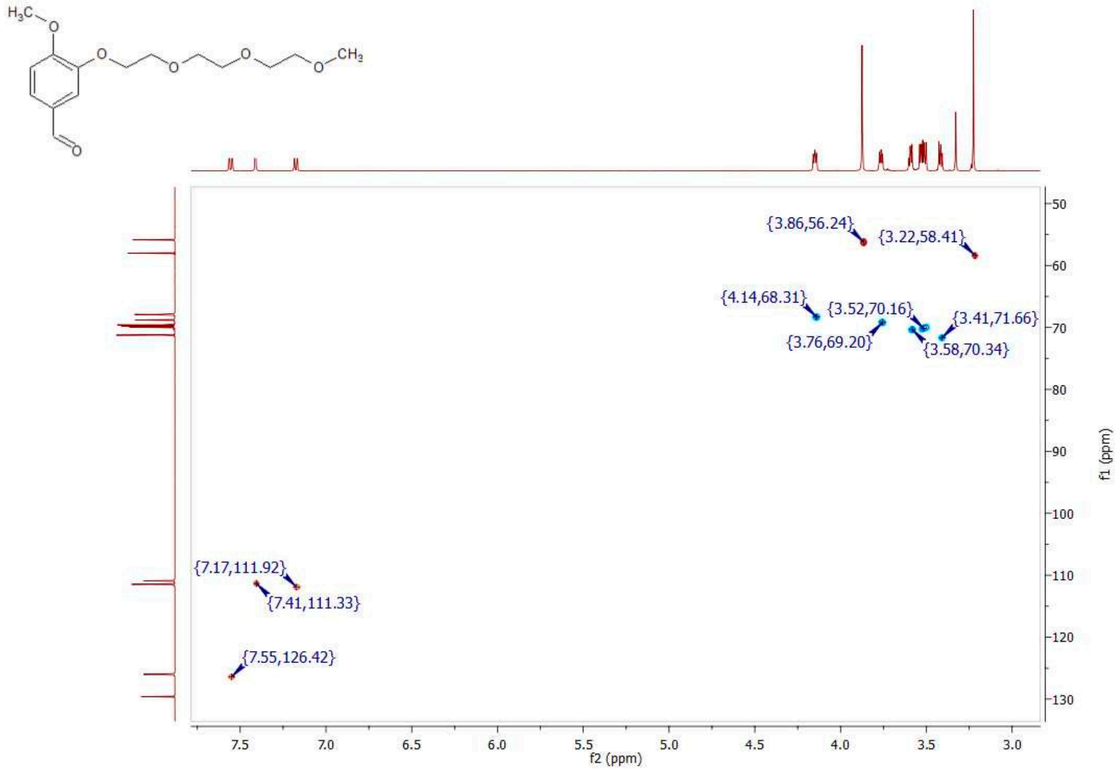

Figure S9. ^{1}H-^{13}C HSQC spectrum of aldehyde **2**.

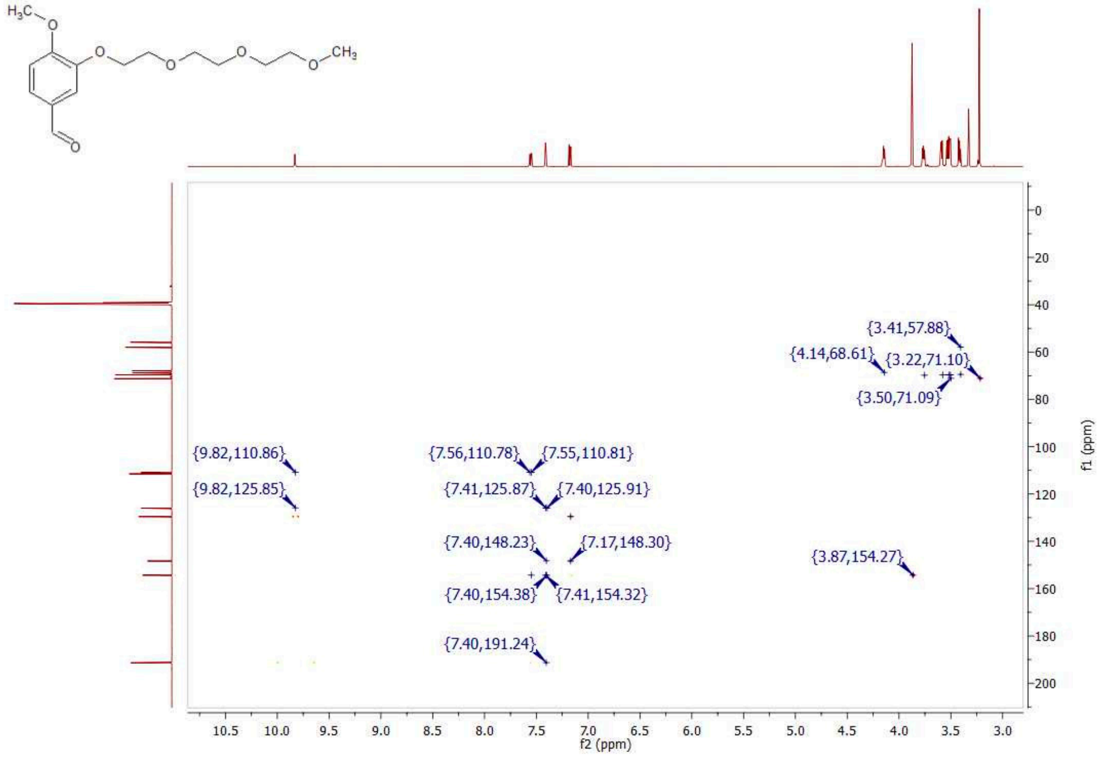

Figure S10. ^{1}H-^{13}C HMBC spectrum of aldehyde **2**.

1.3. NMR experiments of compound **3**.

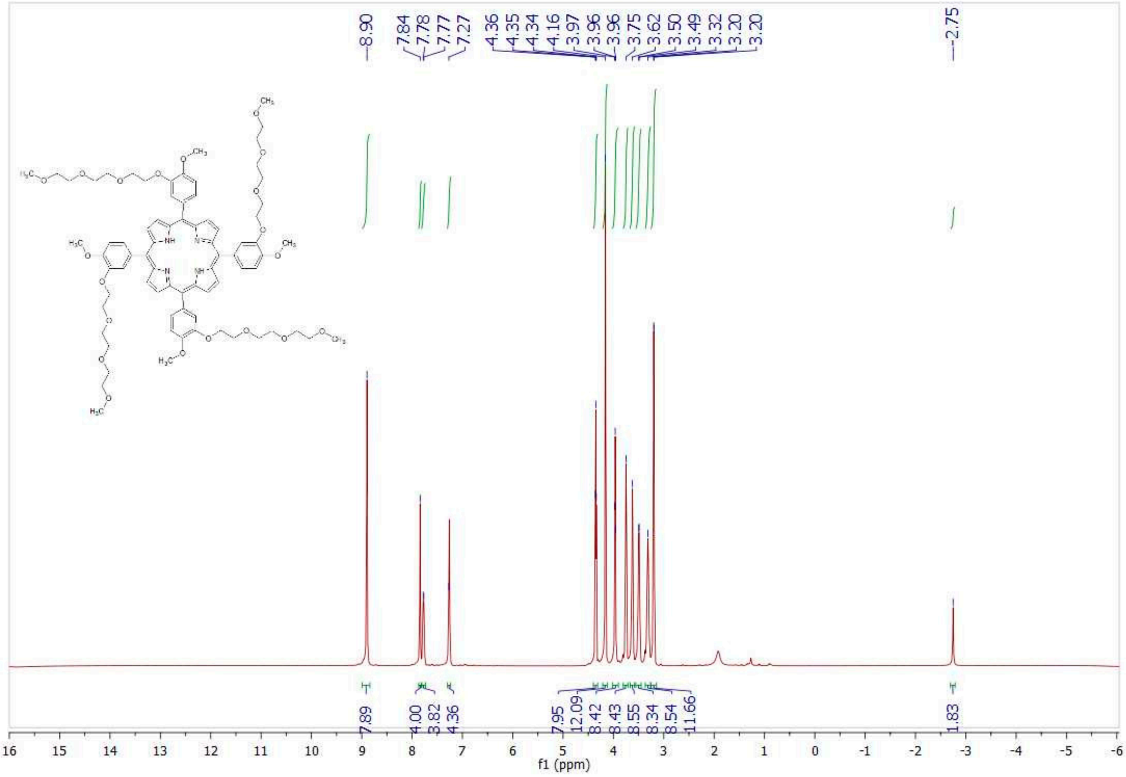

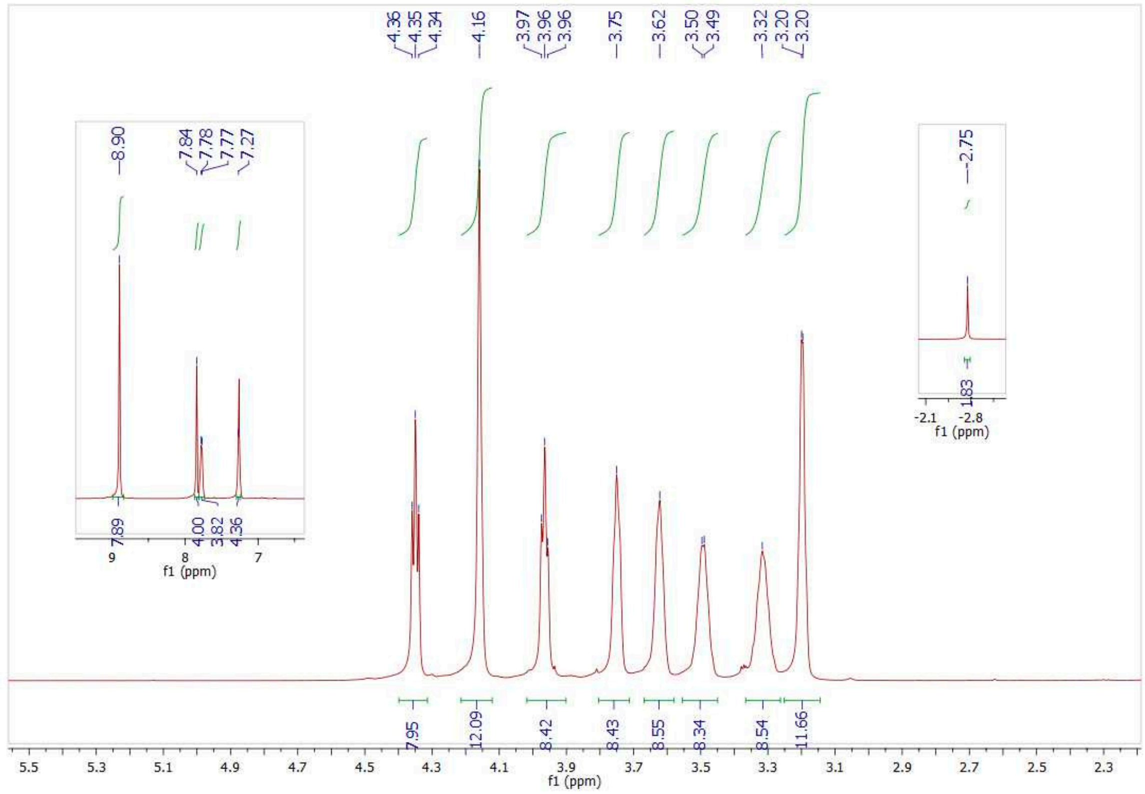

Figure S11. ^{1}H spectra NMR of porphyrin **3**.

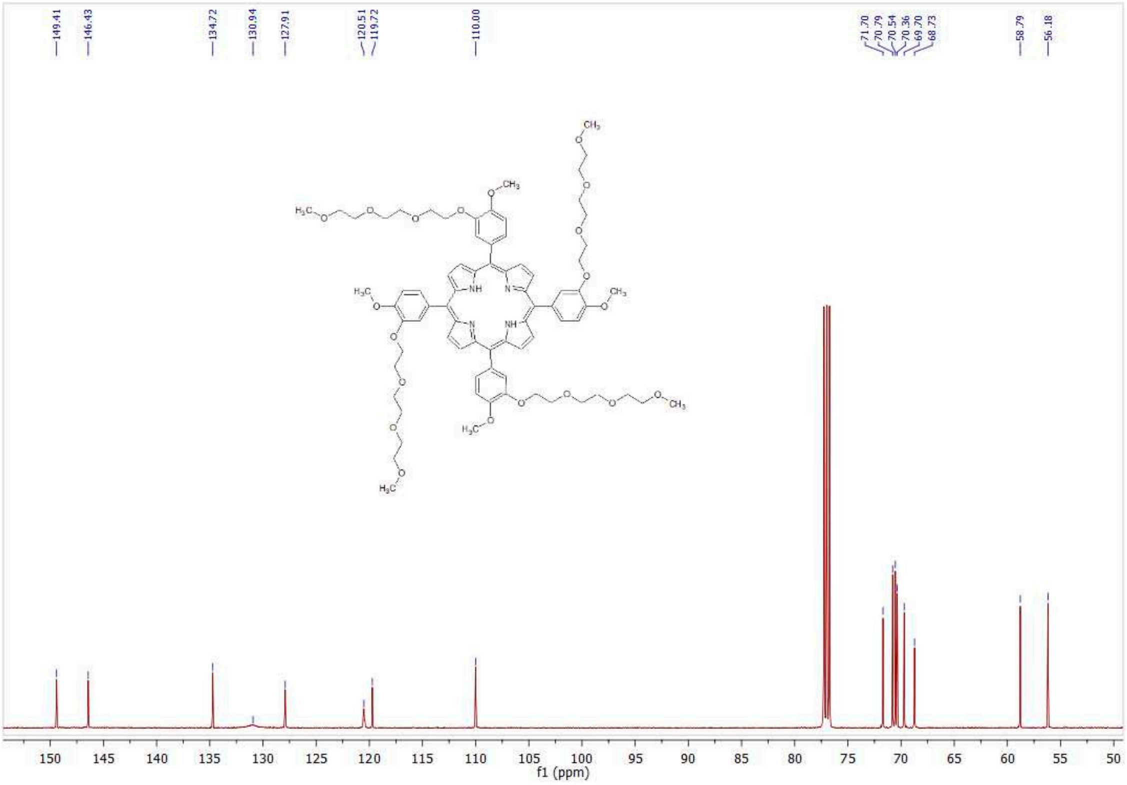

Figure S12. ^{13}C spectrum NMR of porphyrin **3**.

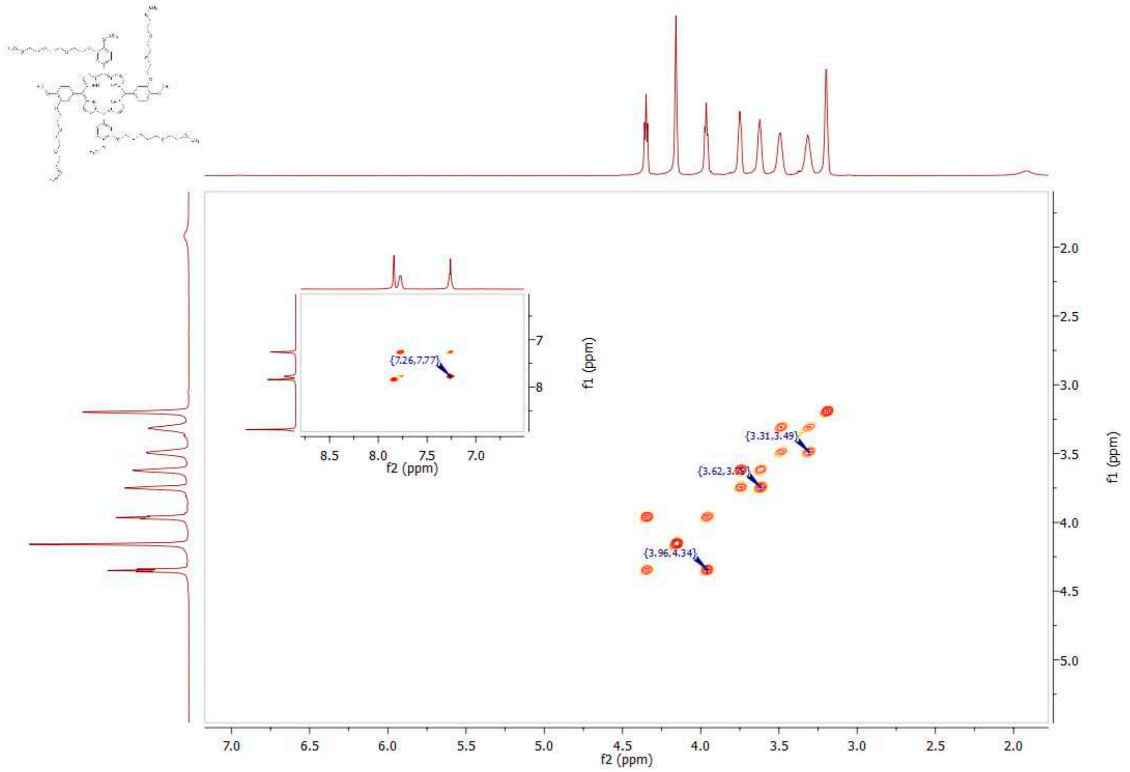

Figure S13. ^{1}H-^{1}H COSY spectrum of porphyrin **3**.

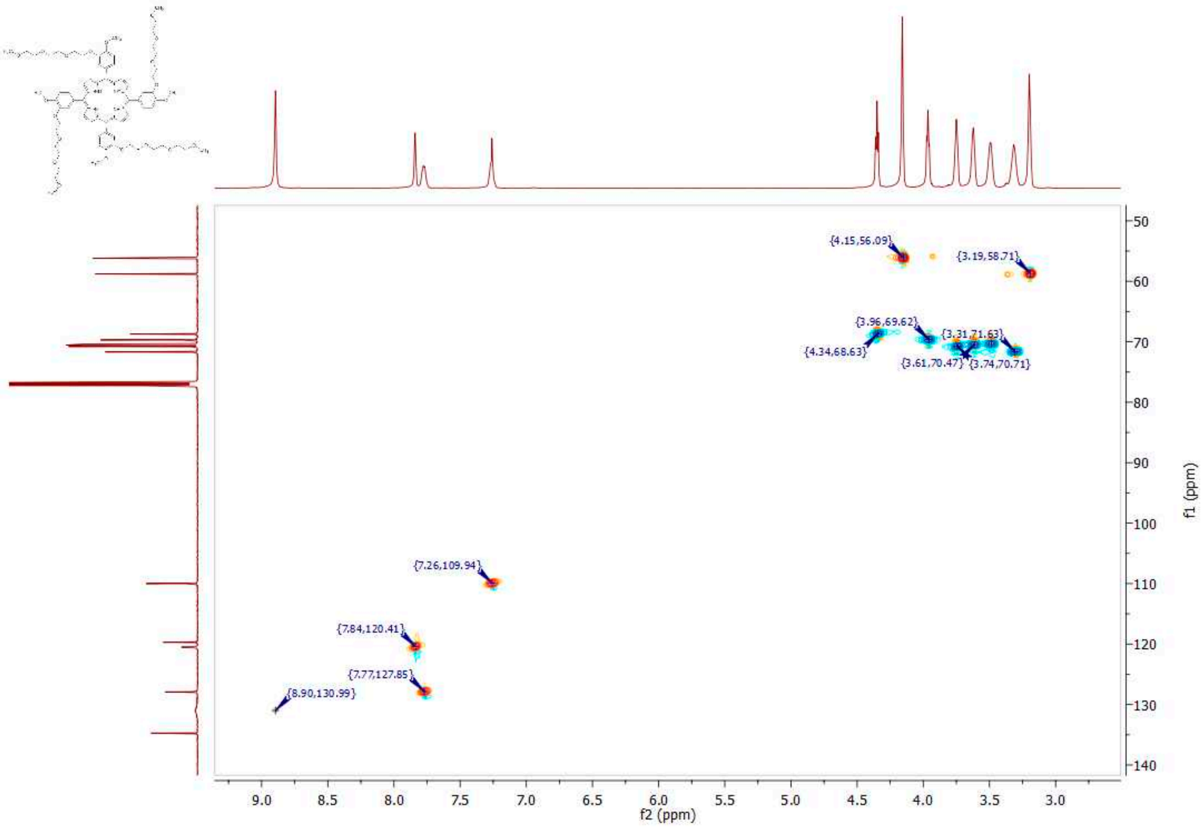

Figure S14. ^{1}H-^{13}C HSQC spectrum of porphyrin **3**.

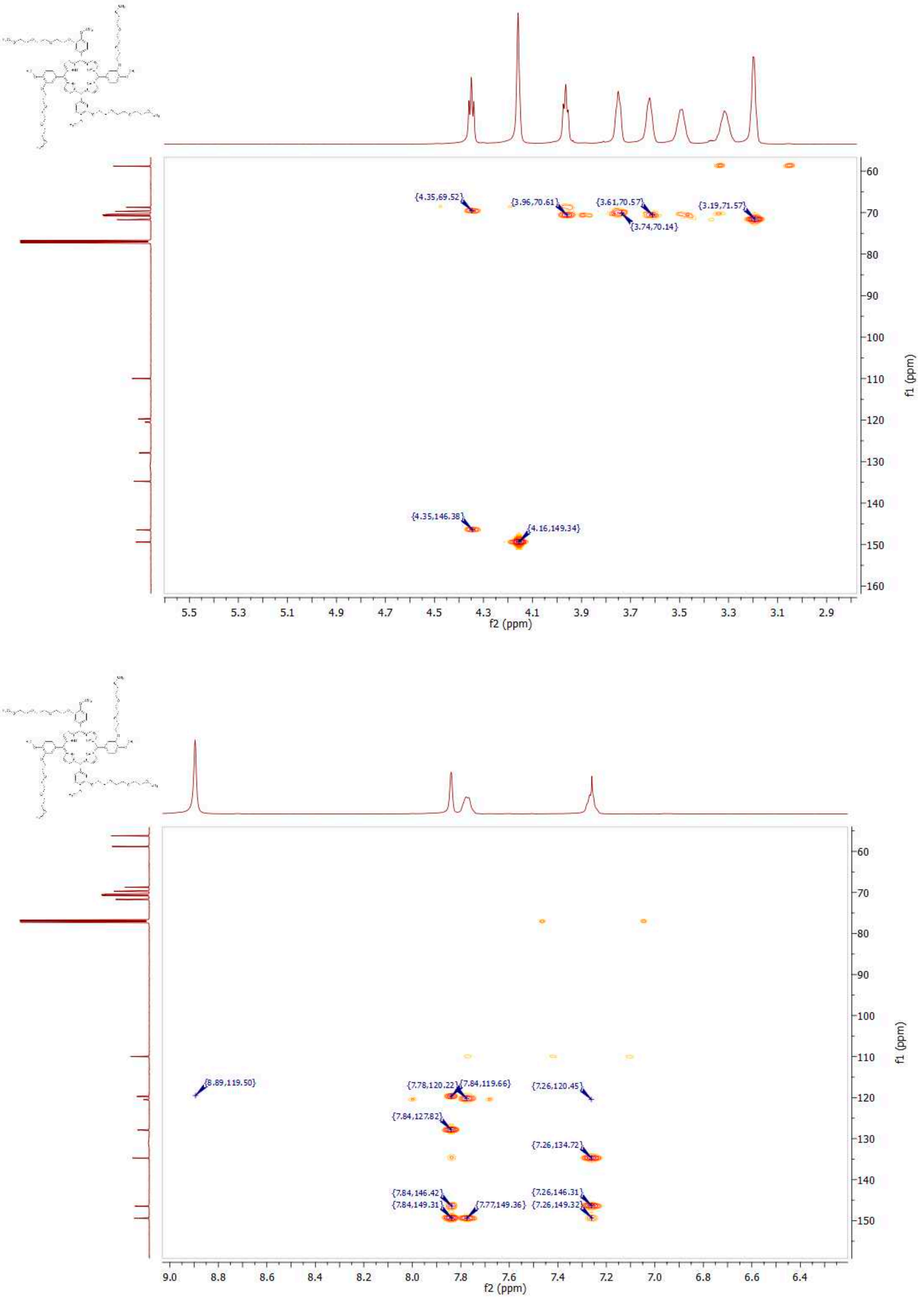

Figure S15. ^{1}H-^{13}C HMBC spectra of porphyrin **3.**

1.4. NMR experiments of compound **4**.

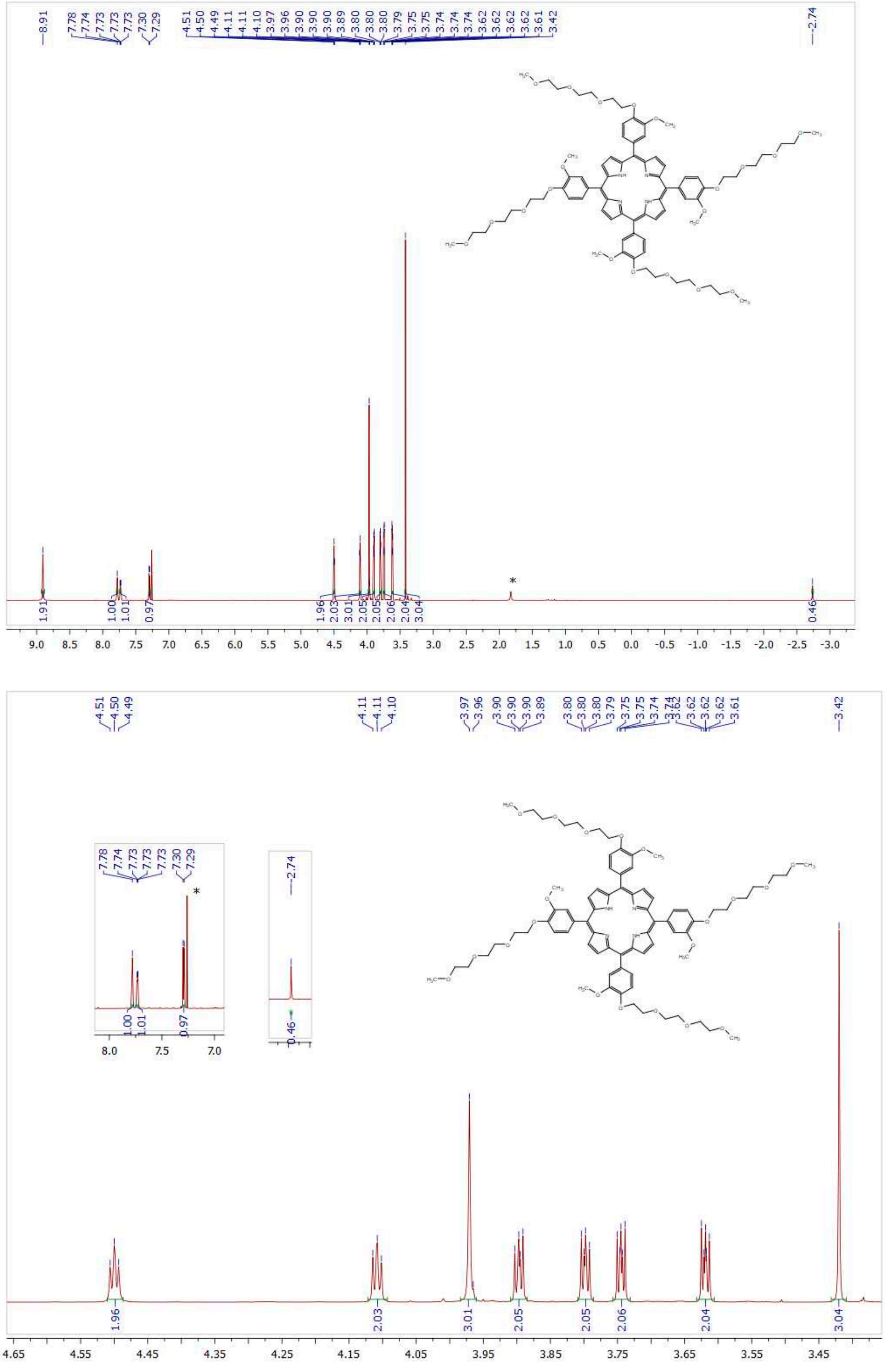

Figure S16. ^{1}H NMR spectra of porphyrin **4**.

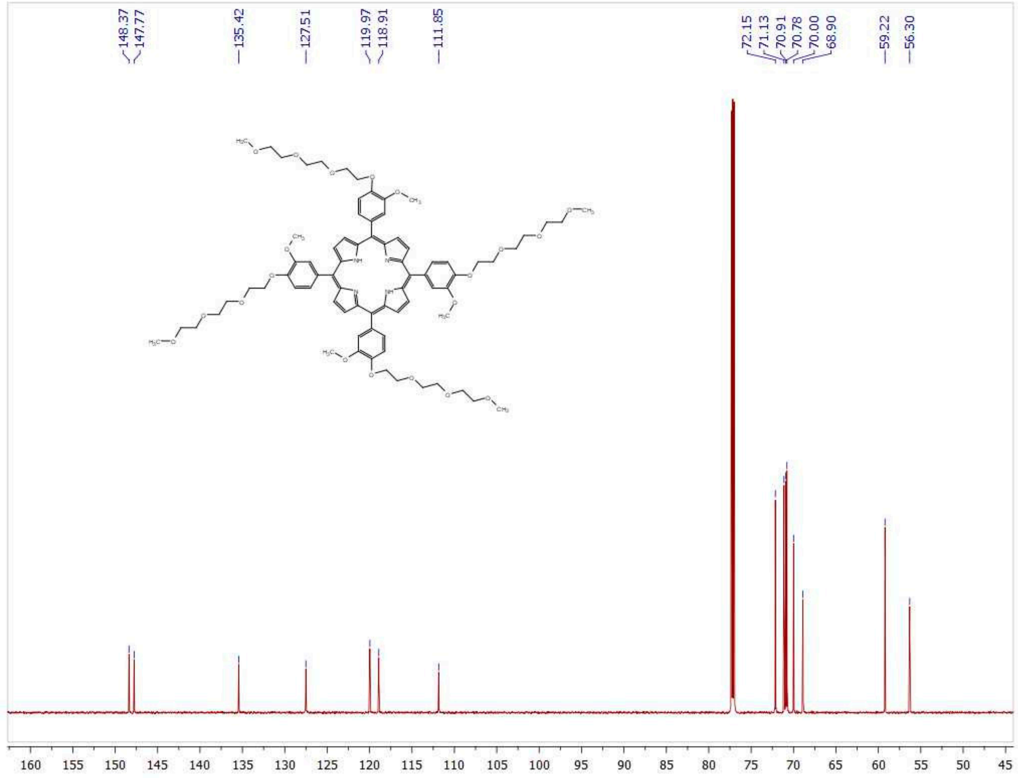

Figure S17. ^{13}C NMR spectrum of porphyrin **4**.

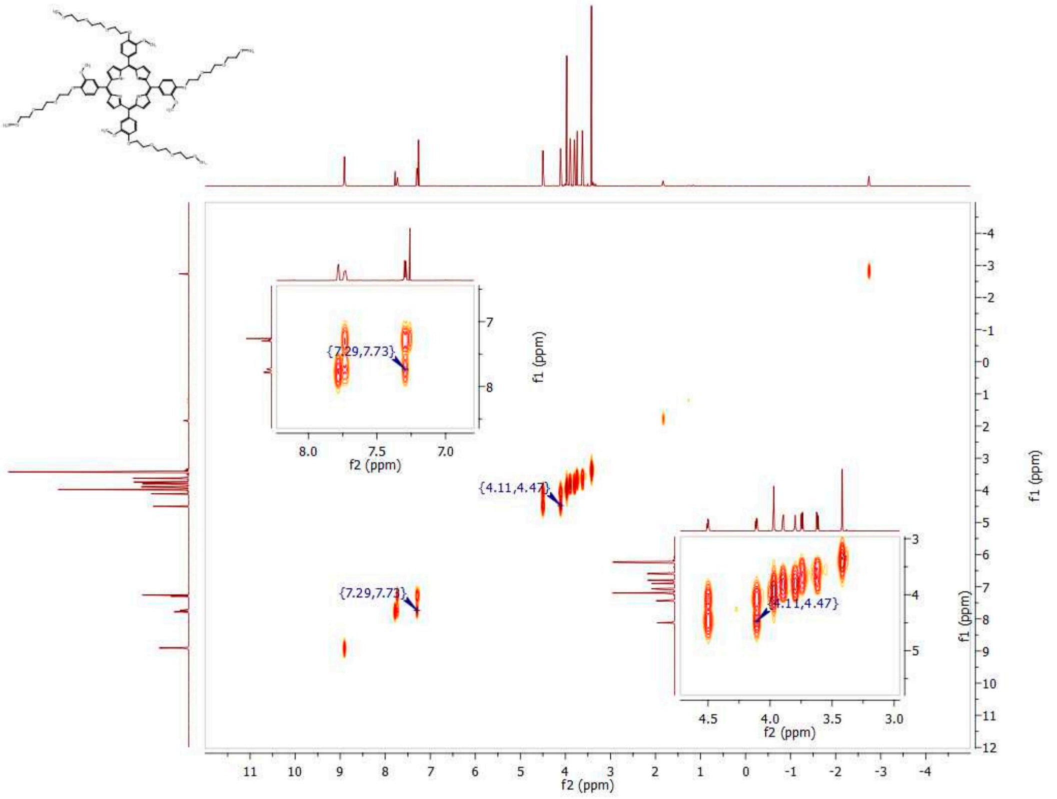

Figure S18. ^{1}H-^{1}H COSY spectrum of porphyrin **4**.

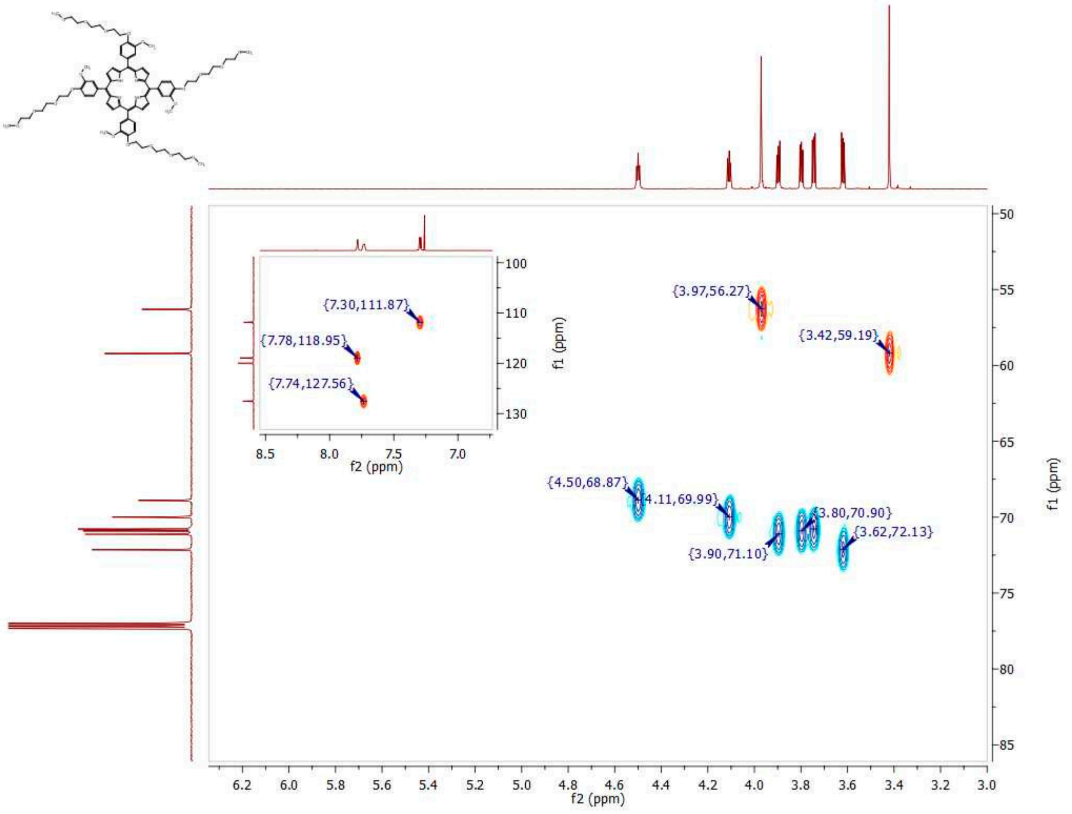

Figure S19. ^{1}H-^{13}C HSQC spectrum of porphyrin **4**.

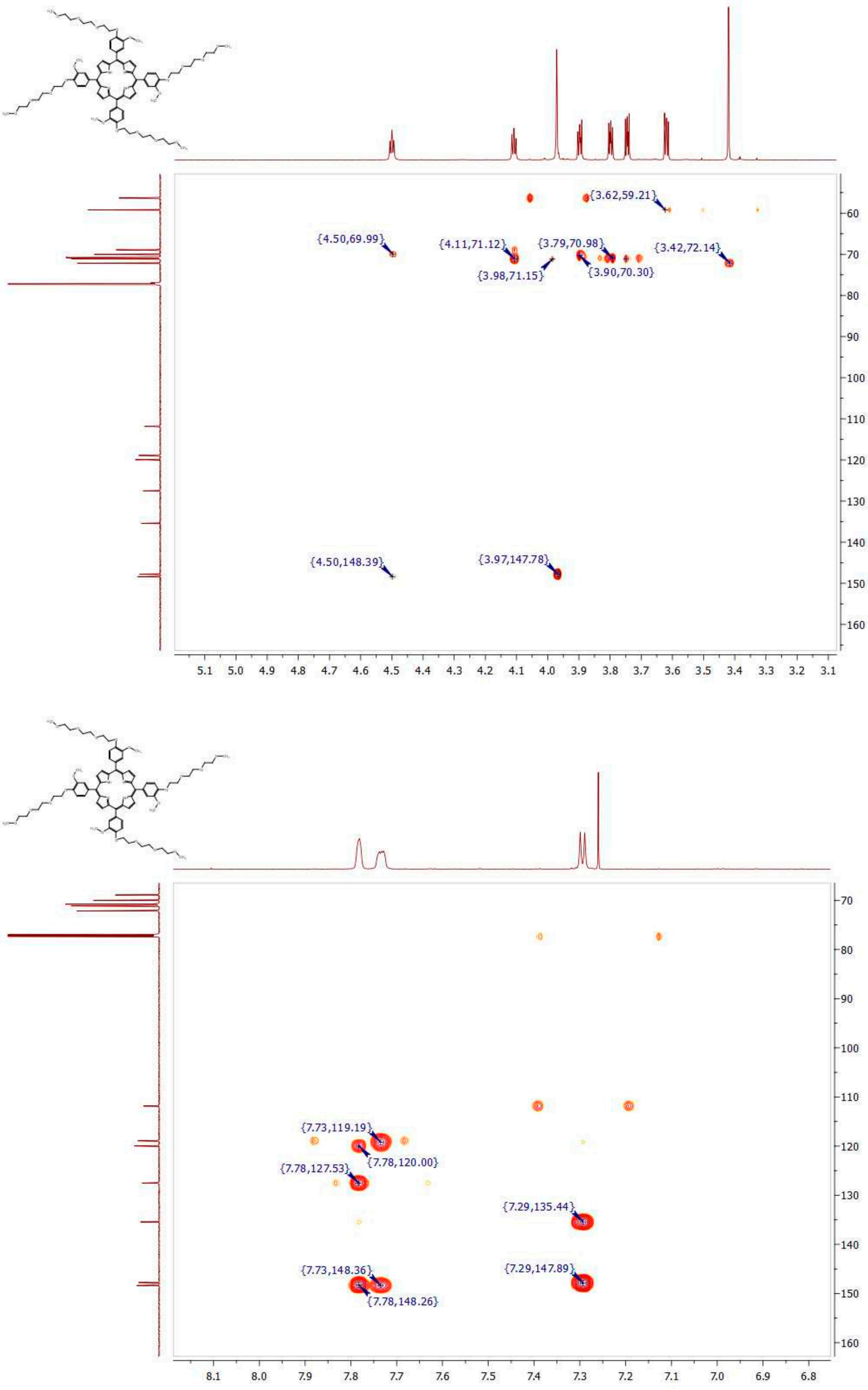

Figure S20. ^{1}H-^{13}C HMBC spectra of porphyrin **4.**

1.5. NMR experiments of compound **5**.

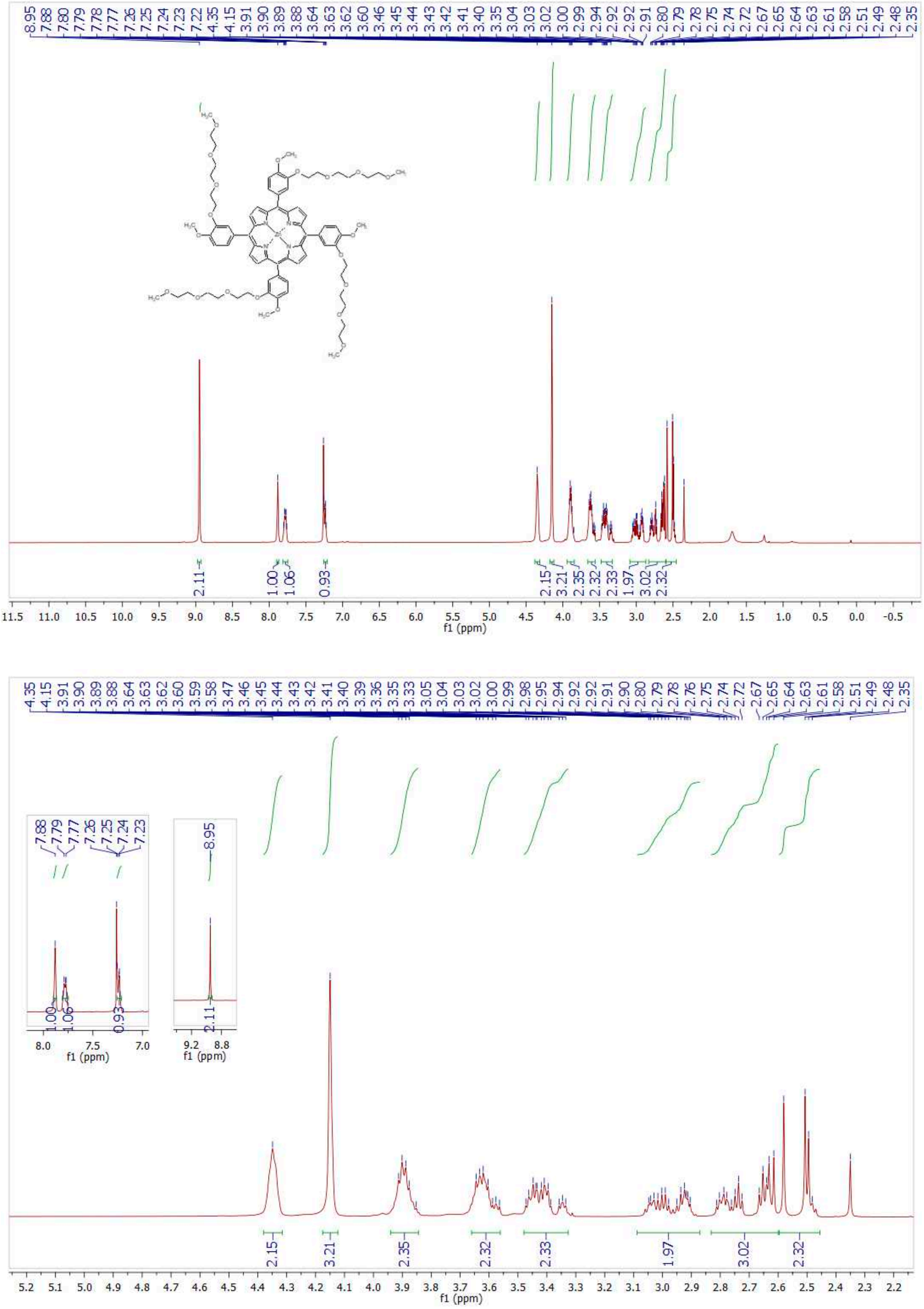

Figure S21. ^{1}H NMR spectra of porphyrin **5**.

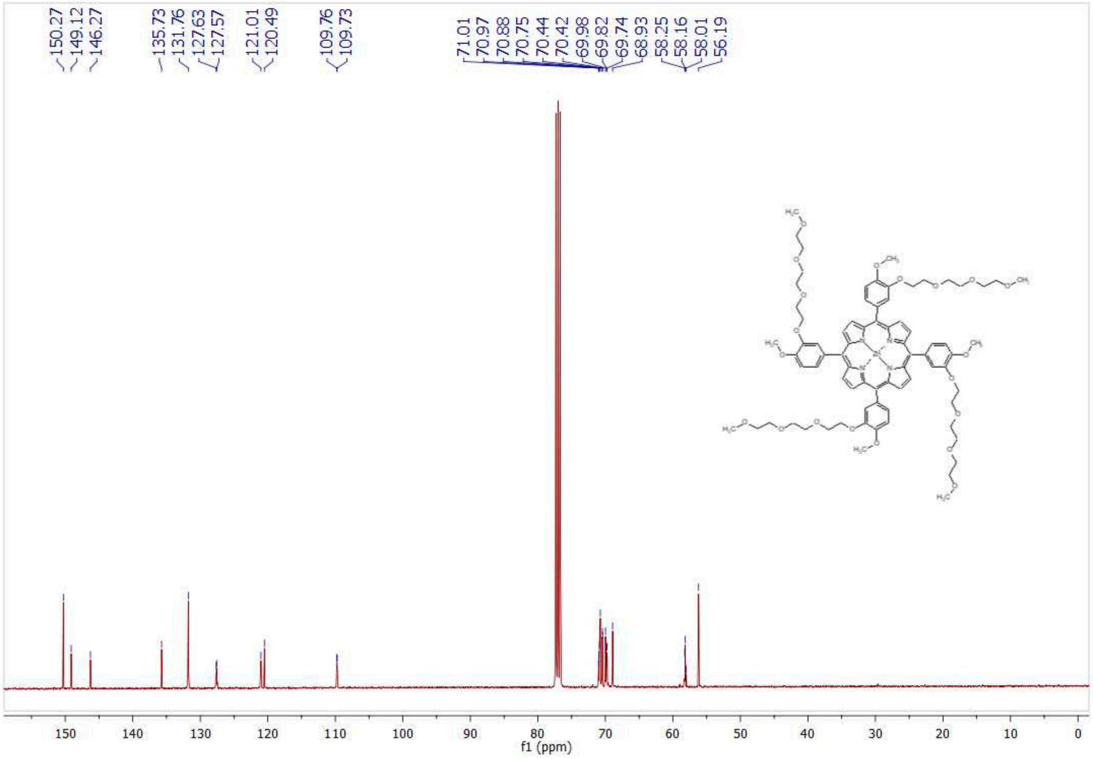

Figure S22. ^{13}C NMR spectrum of porphyrin **5**.

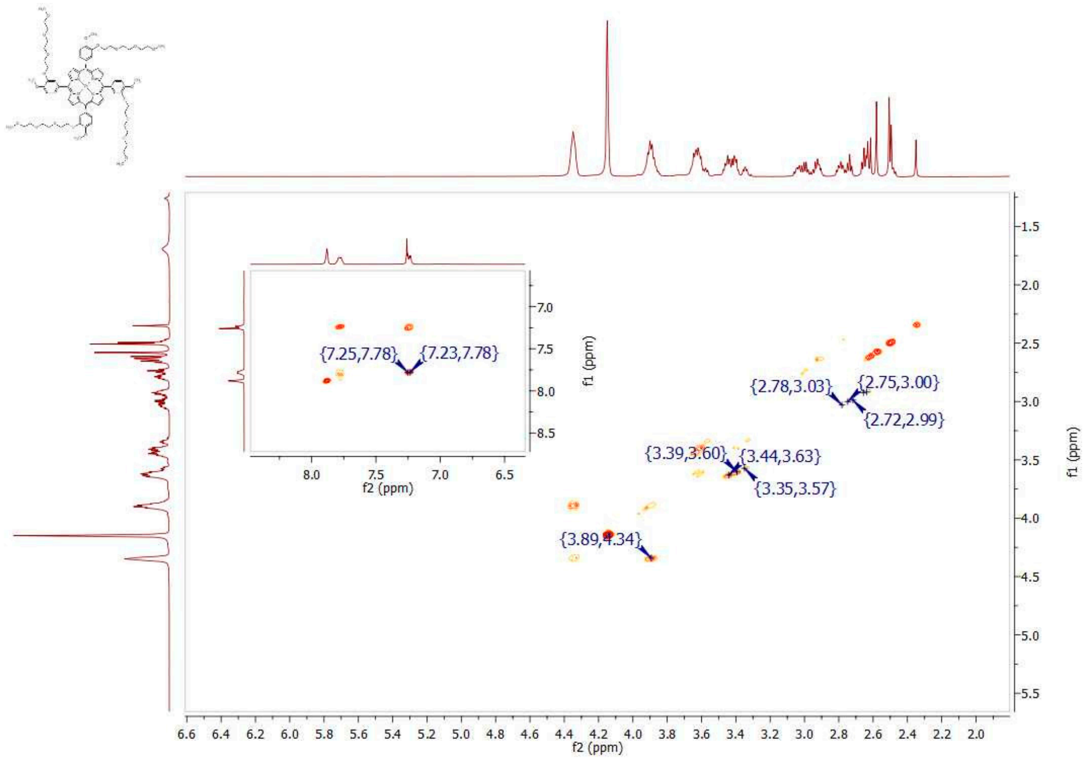

Figure S23. ^{1}H-^{1}H COSY spectrum of porphyrin **5**.

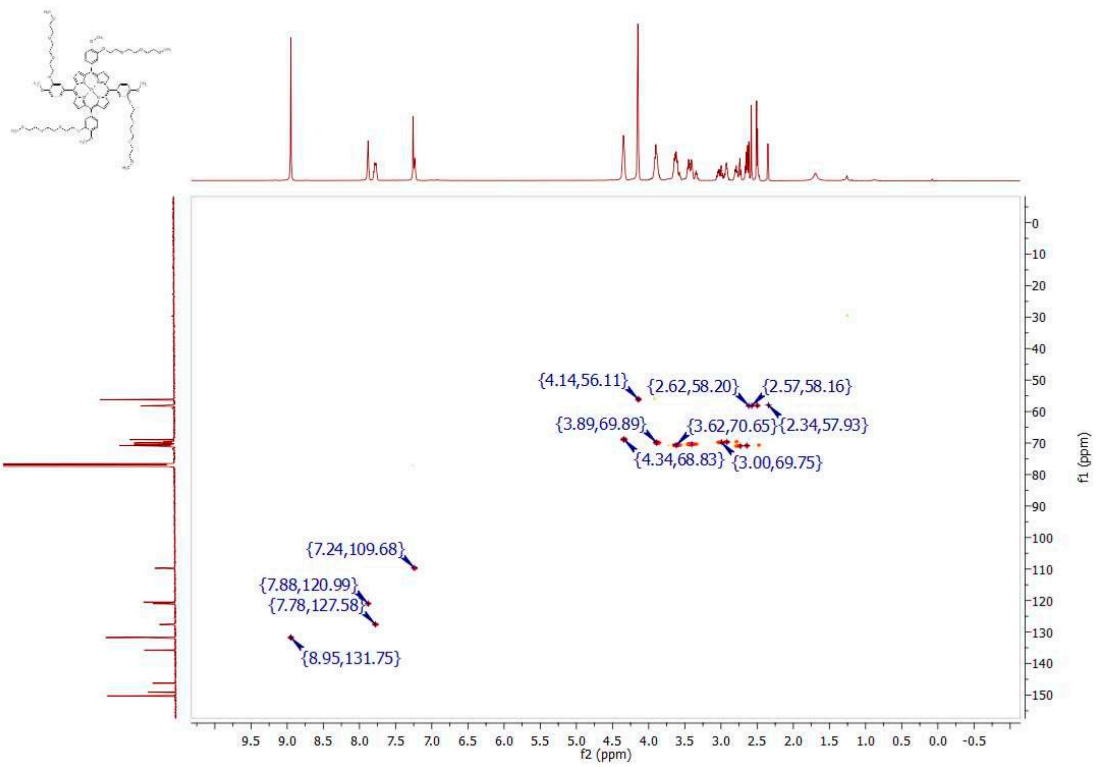

Figure S24. ^{1}H-^{13}C HSQC spectrum of porphyrin **5**.

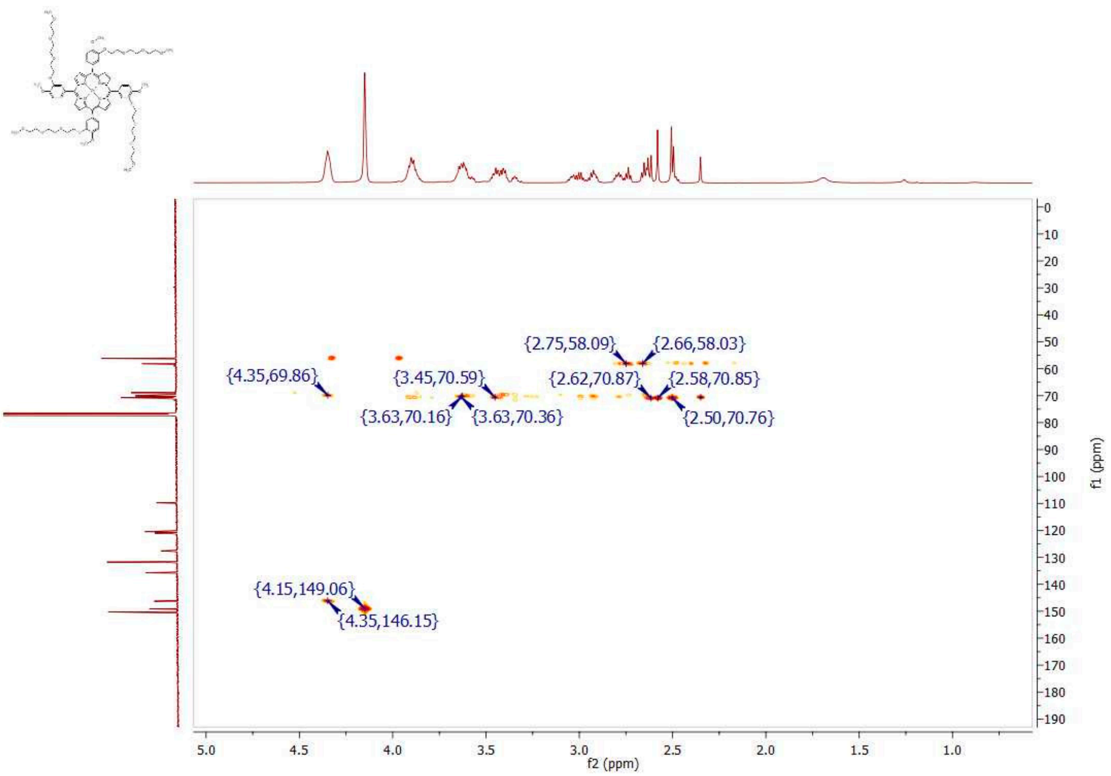

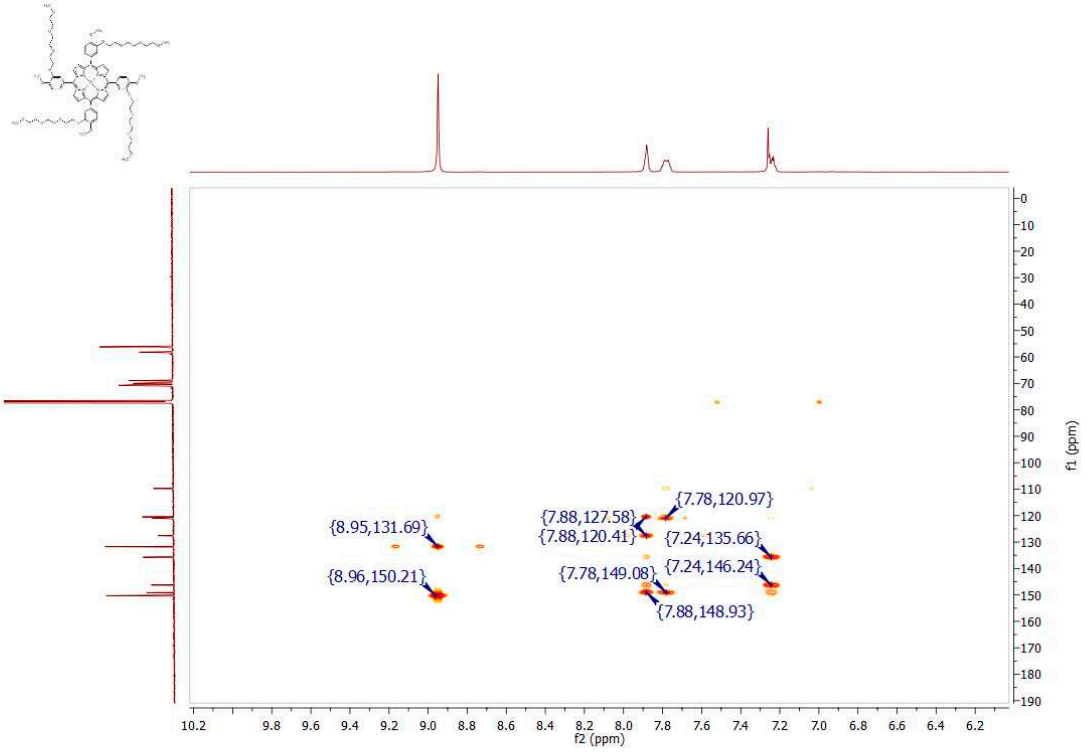

Figure S25. ^{1}H-^{13}C HMBC spectra of porphyrin **5.**

1.6. NMR experiments of compound **6**.

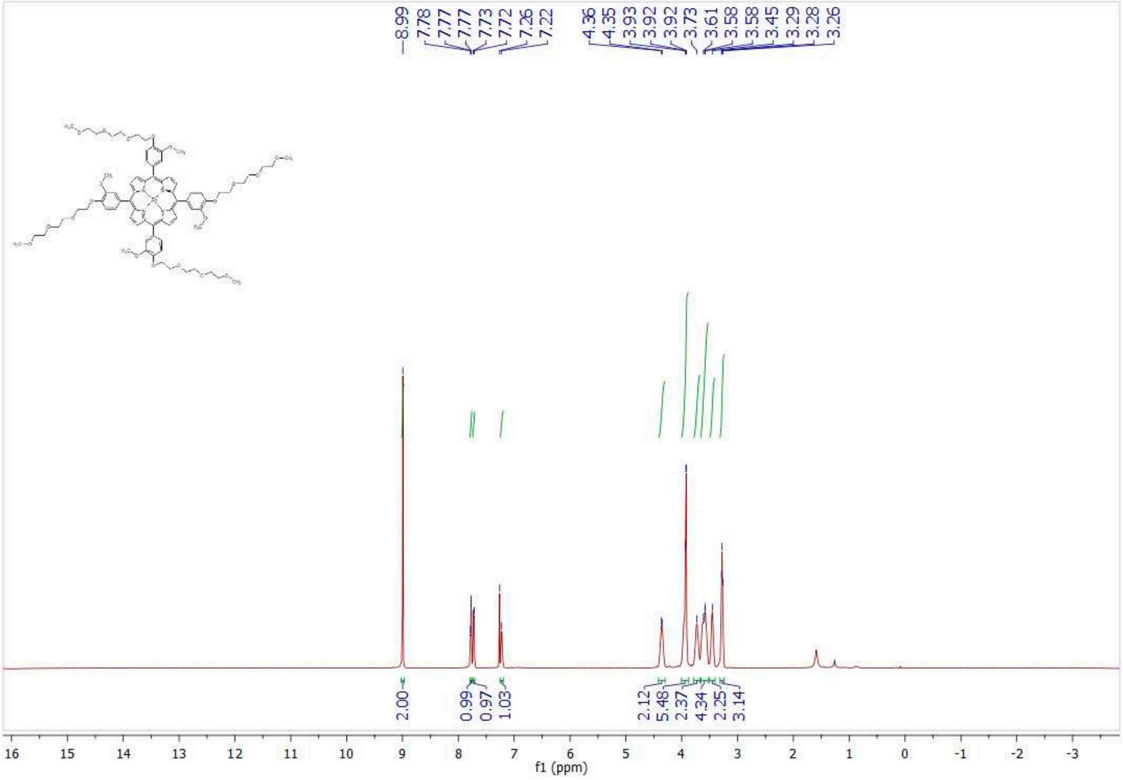

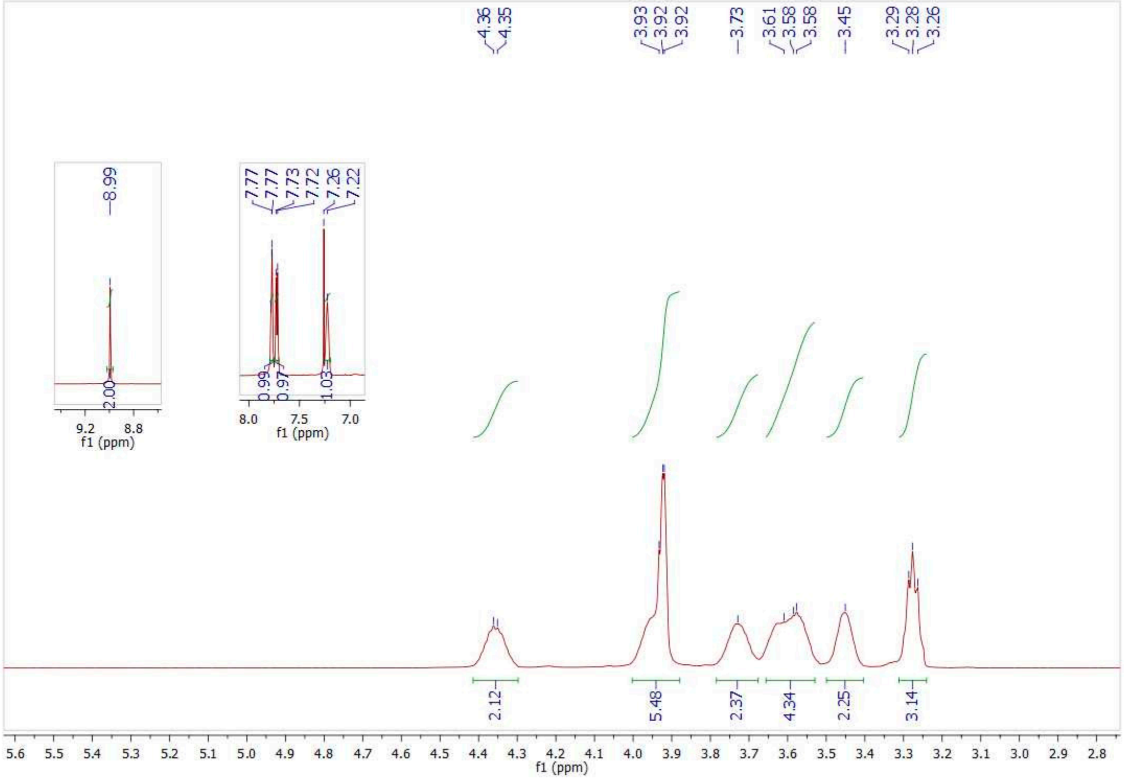

Figure S26. ^{1}H NMR spectra of porphyrin **6**.

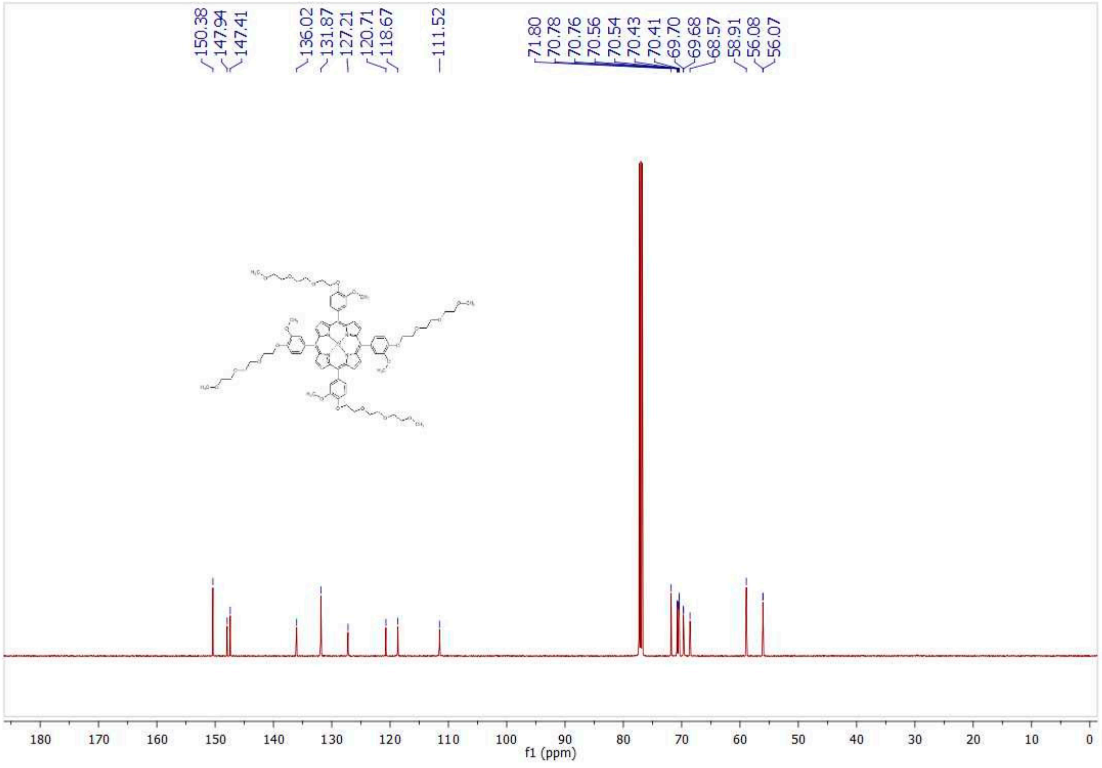

Figure S27. ^{13}C NMR spectrum of porphyrin **6**.

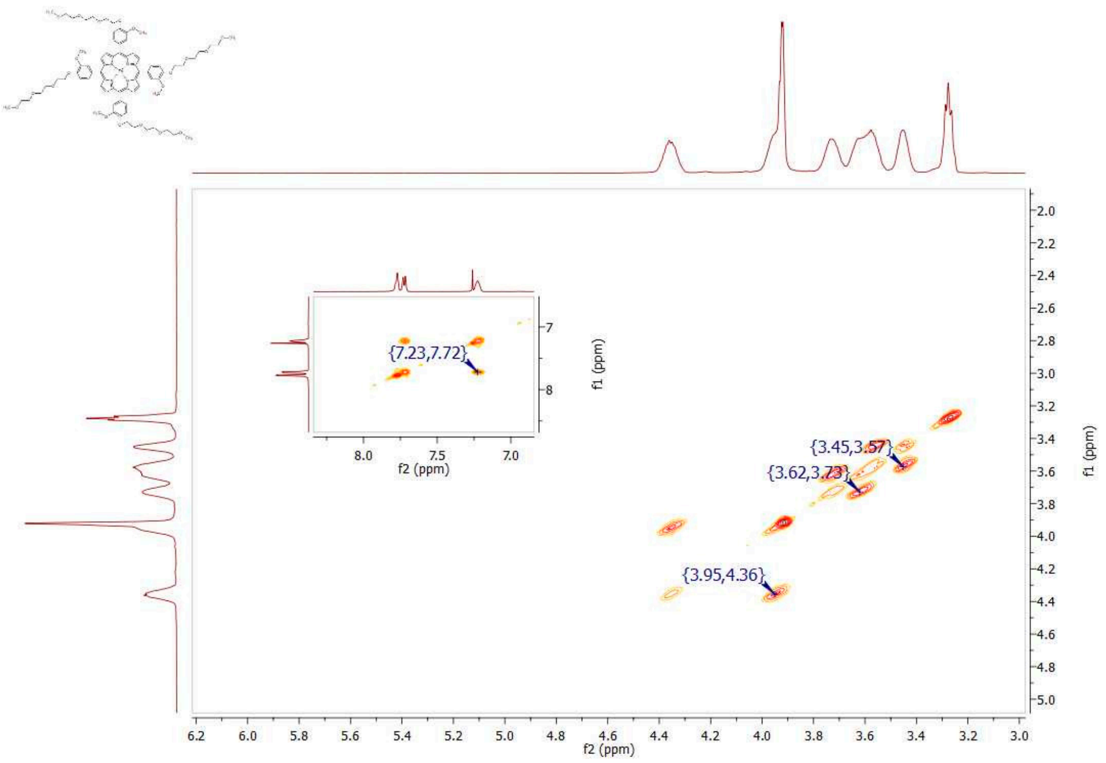

Figure S28. ^{1}H-^{1}H COSY spectrum of porphyrin **5**.

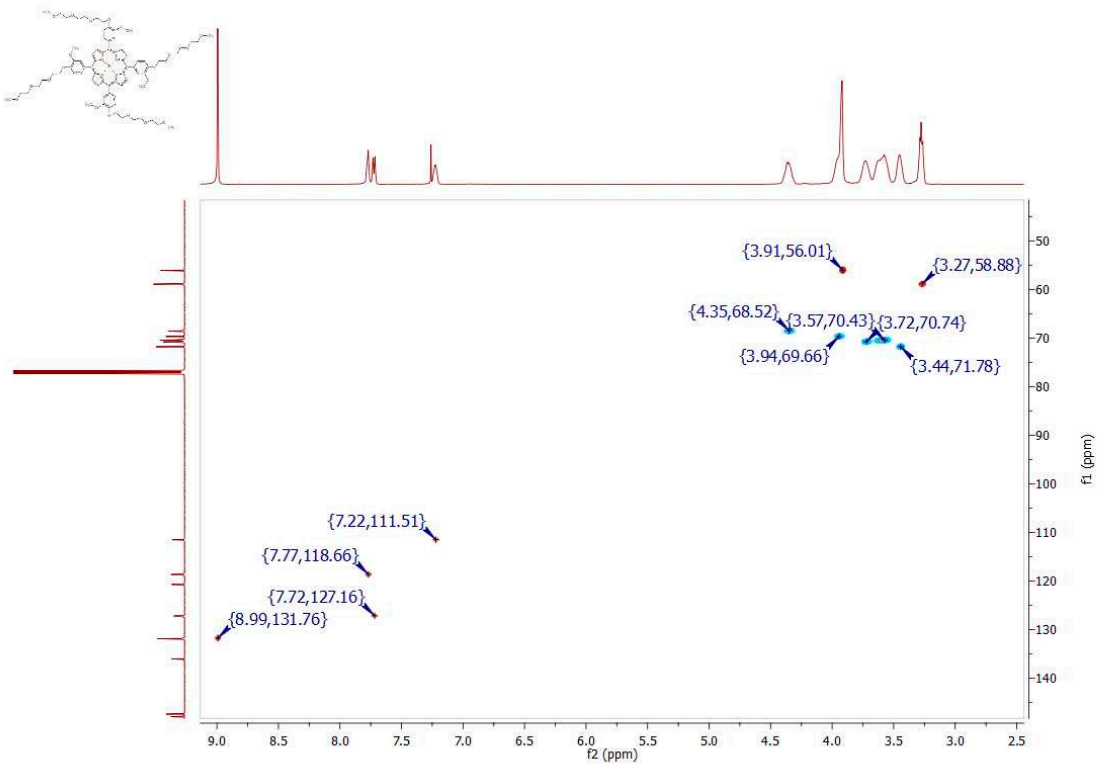

Figure S29. ^{1}H-^{13}C HSQC spectrum of porphyrin **6**.

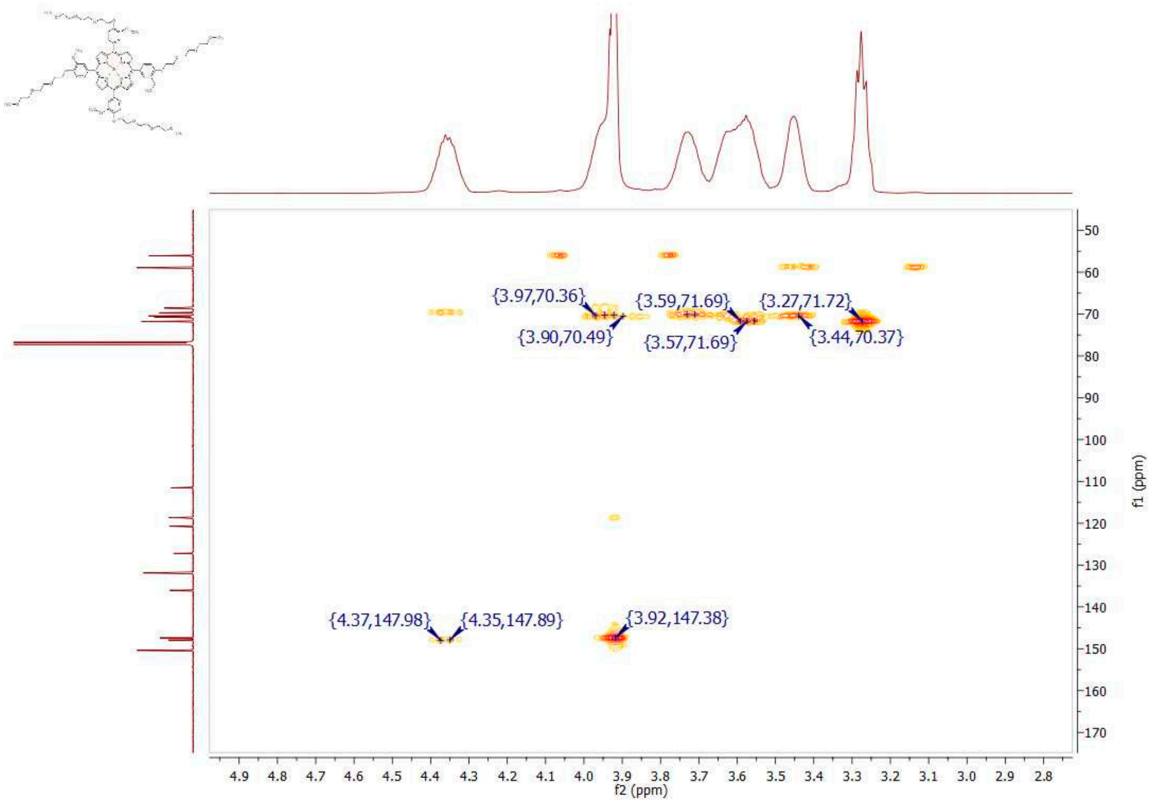

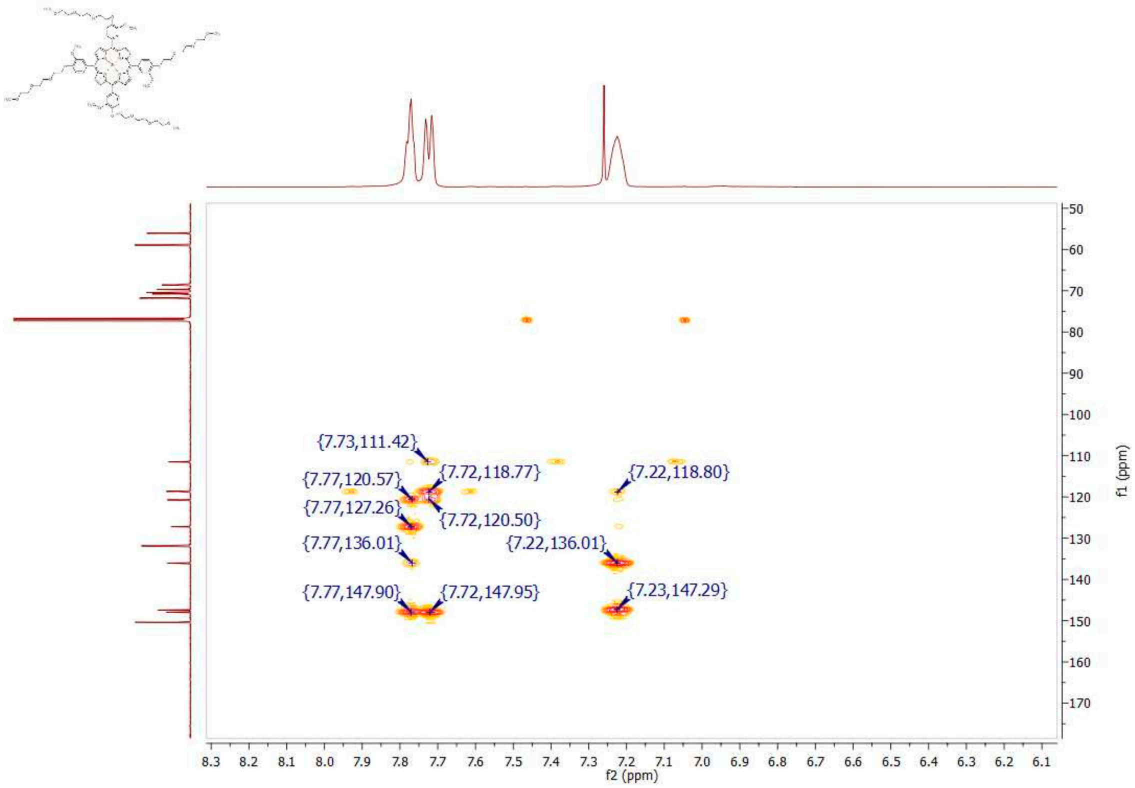

Figure S30. ^{1}H-^{13}C HMBC spectra of porphyrin **6.**

2. Mass spectrometry data

1.7. ESI experiments

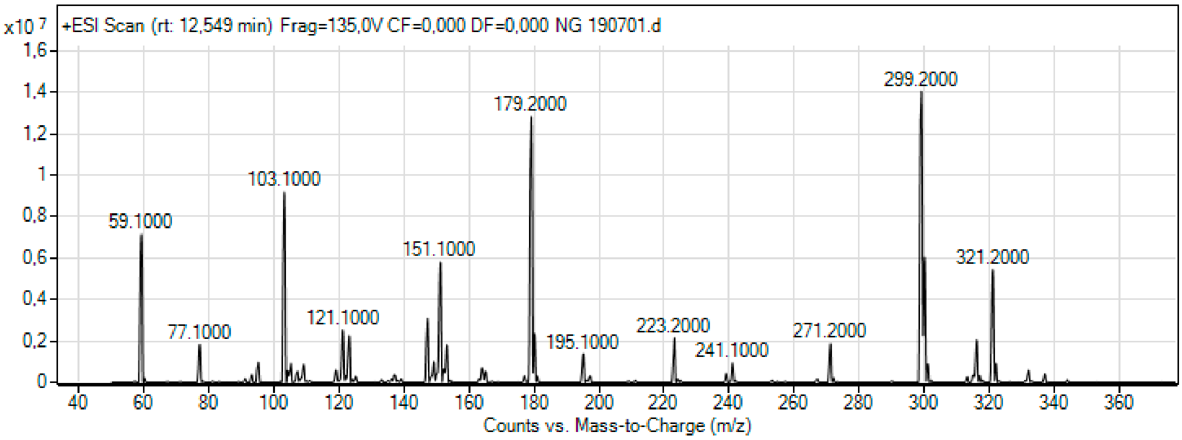

Figure S32. Mass spectra of aldehyde **1.**

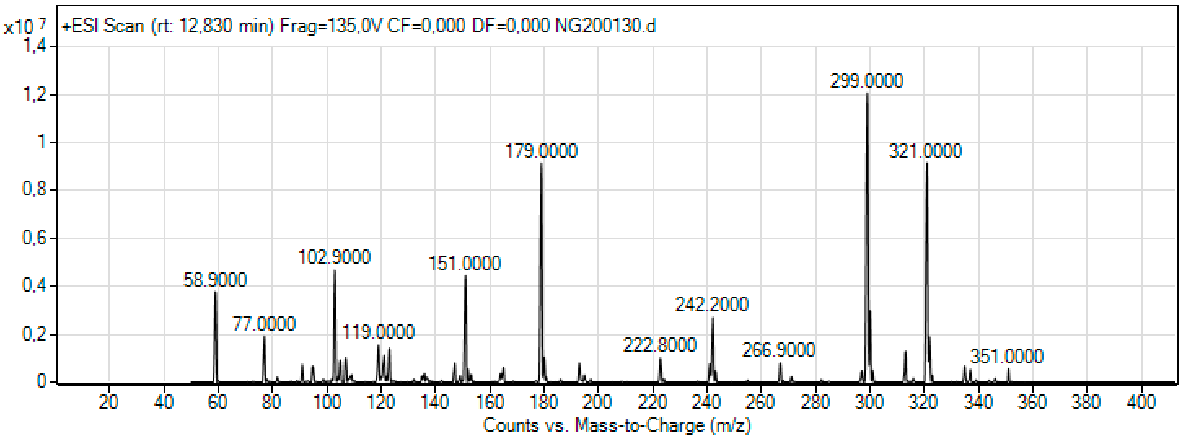

Figure S33. Mass spectra of aldehyde **2.**

1.8. Data from MALDI-TOF experiments

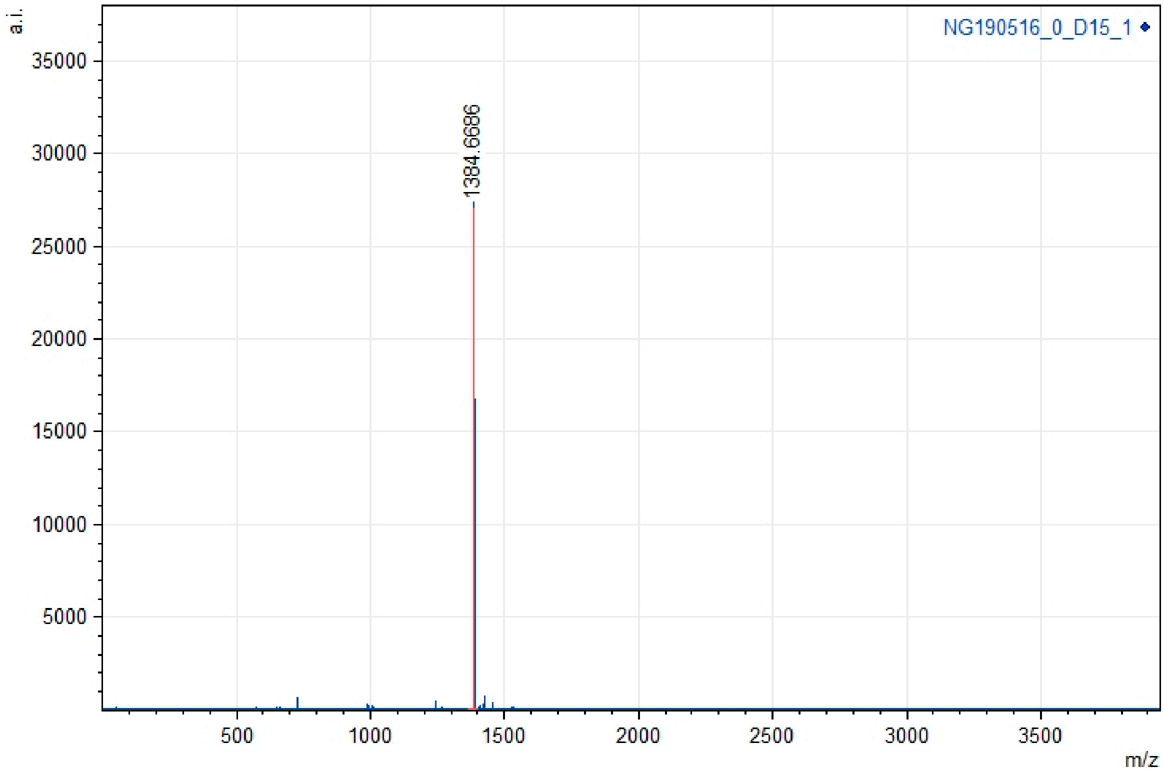

Figure S34. Mass spectra of porphyrin **3**.

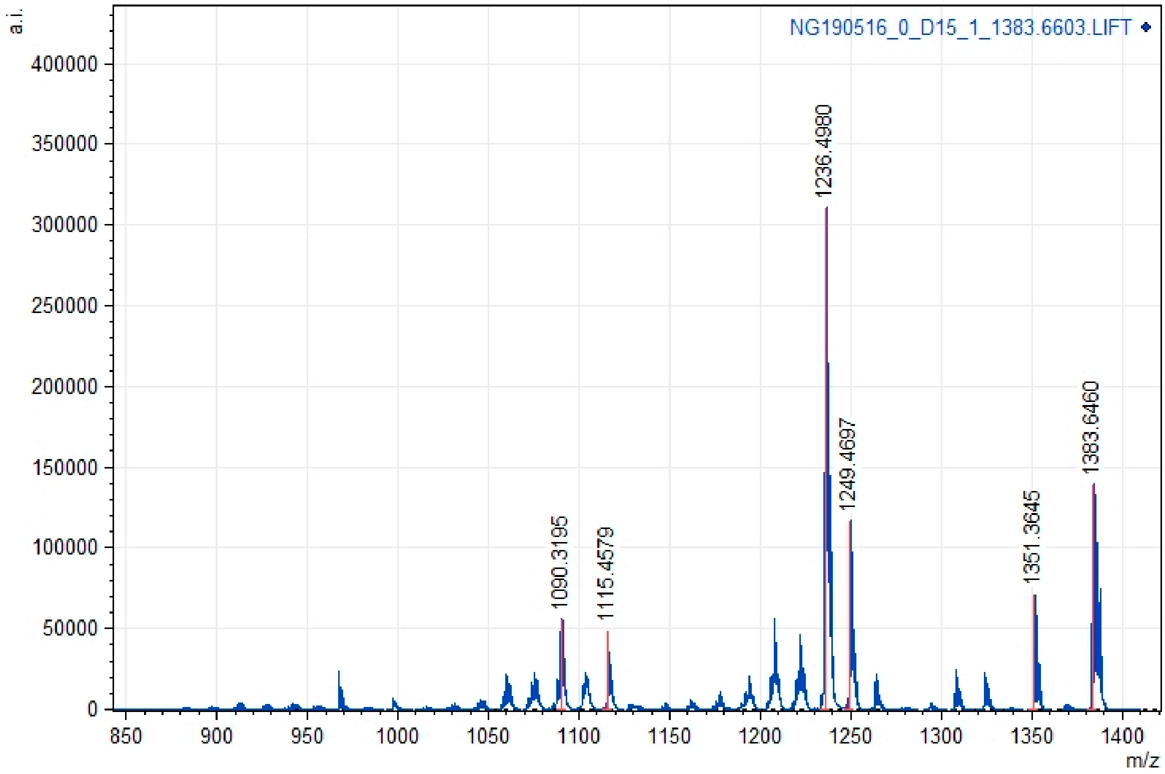

Figure S34. Fragmentation mass spectra of porphyrin **3.**

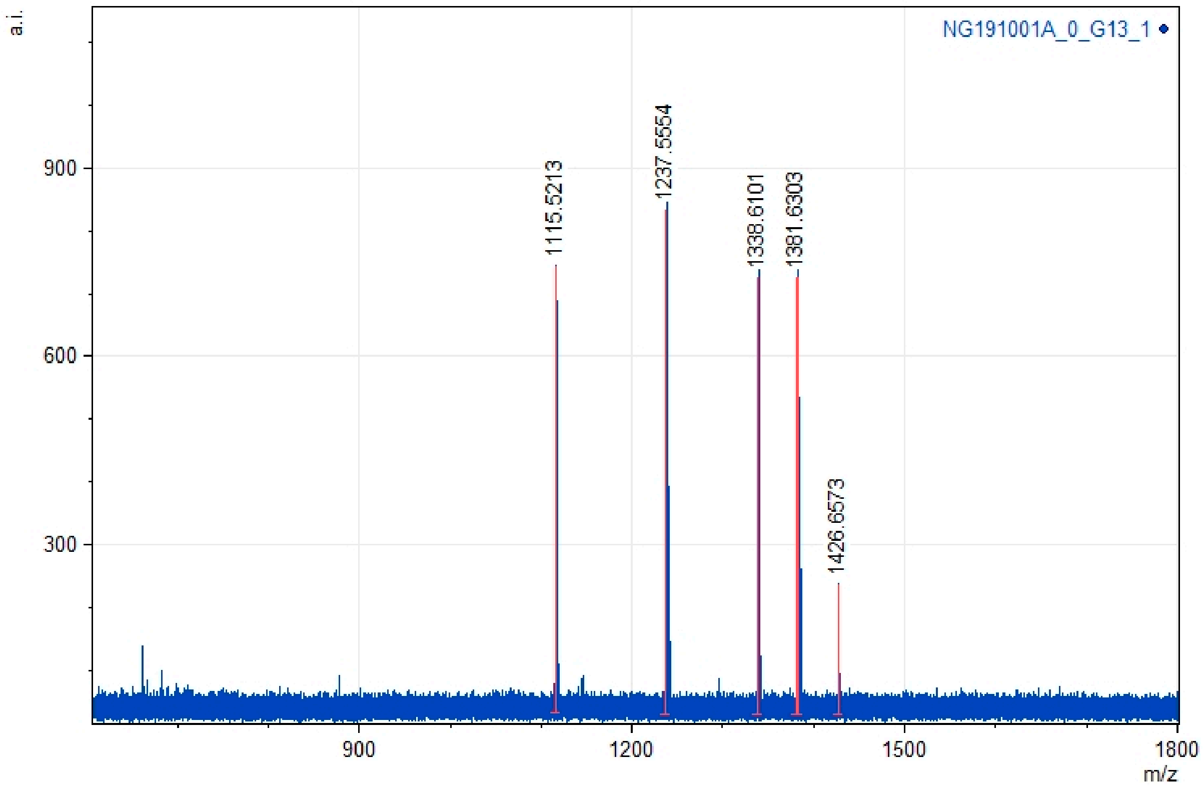

Figure S35. Mass spectra of porphyrin **4**.

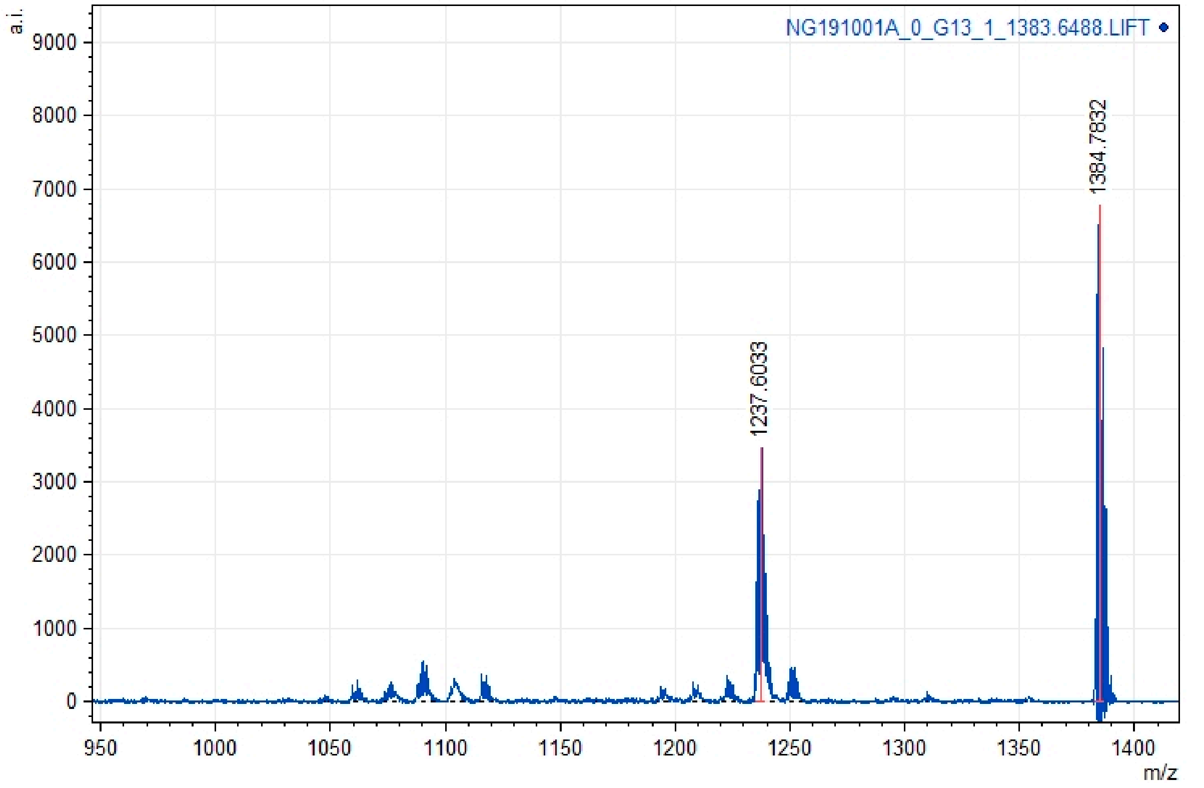

Figure S36. Fragmentation mass spectra of porphyrin **4**.

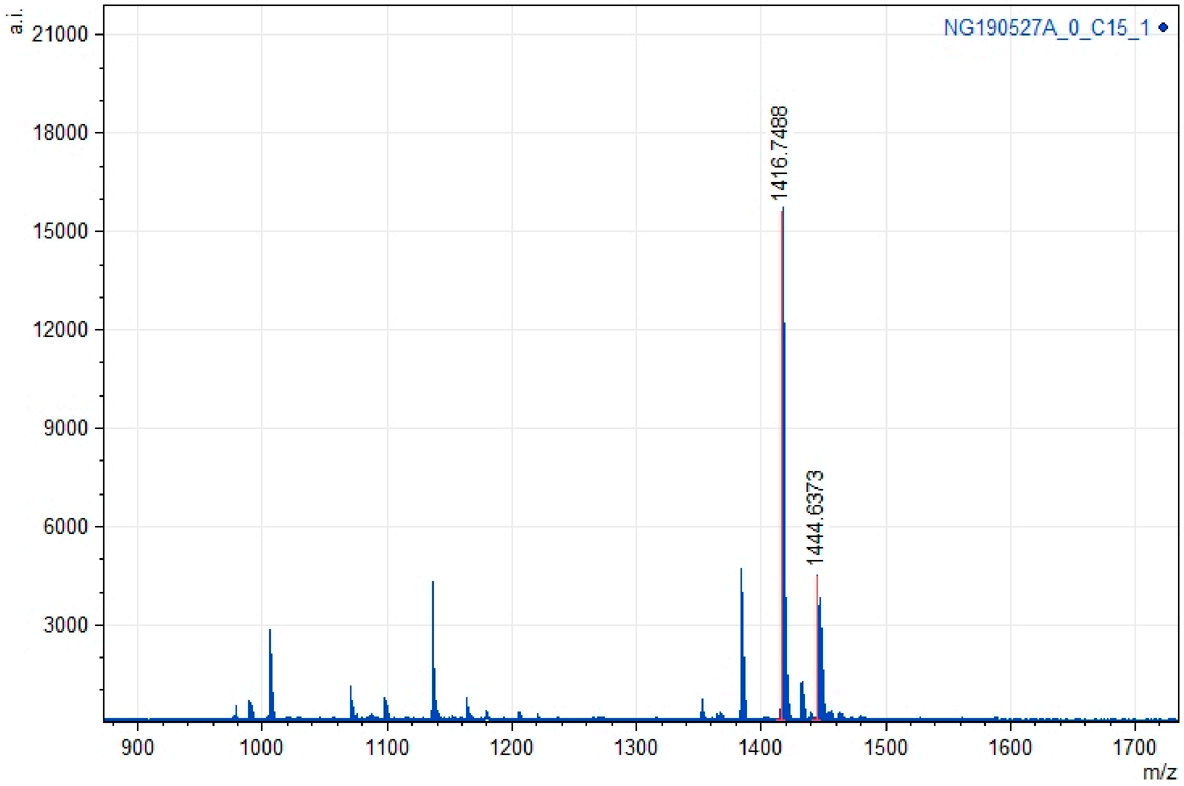

Figure S37. Mass spectra of porphyrin **5**.

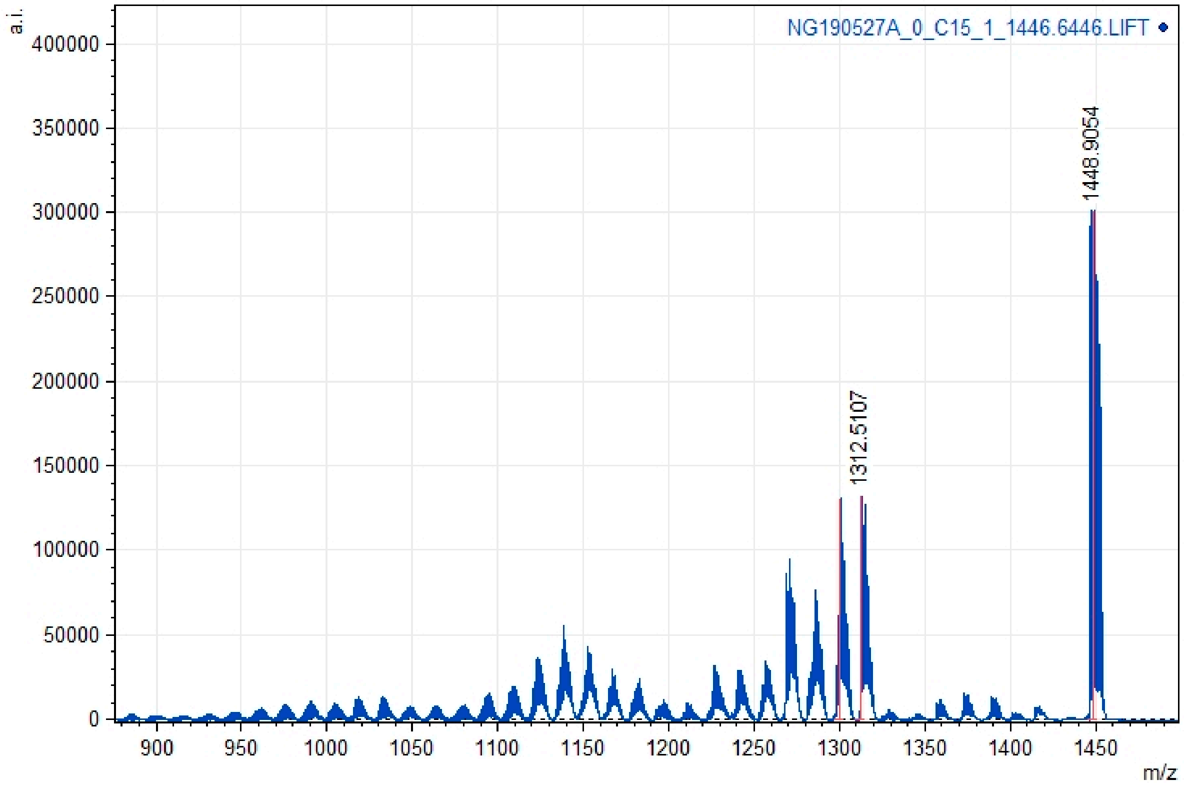

Figure S38. Fragmentation mass spectra of porphyrin **5**.

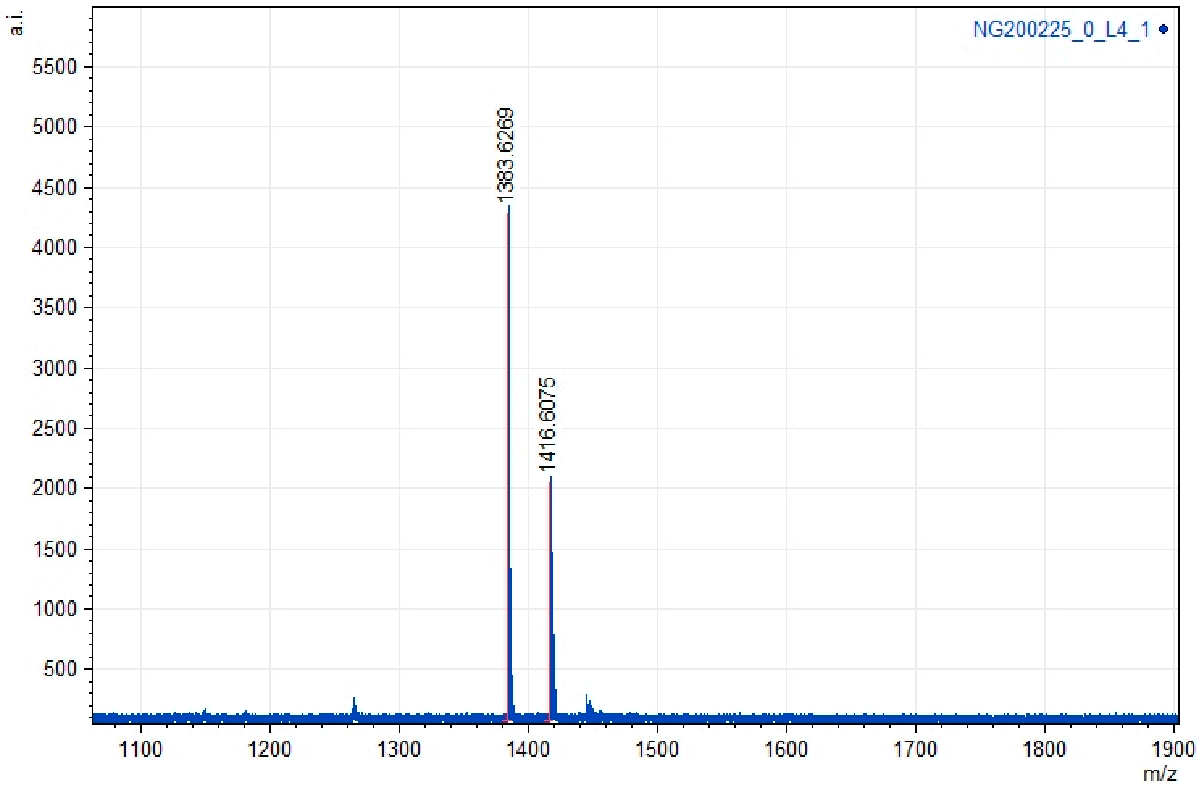

Figure S39. Fragmentation mass spectra of porphyrin **6**.

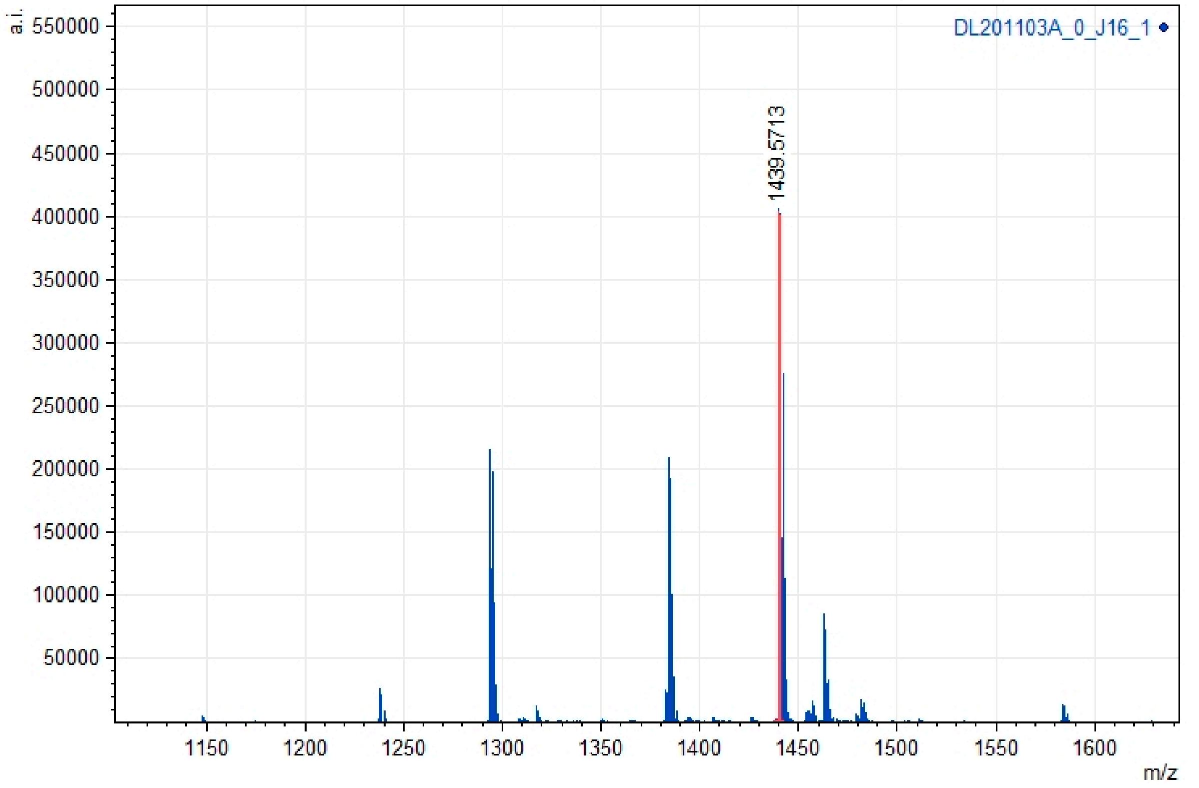

Figure S40. Mass spectra of porphyrin **7**.

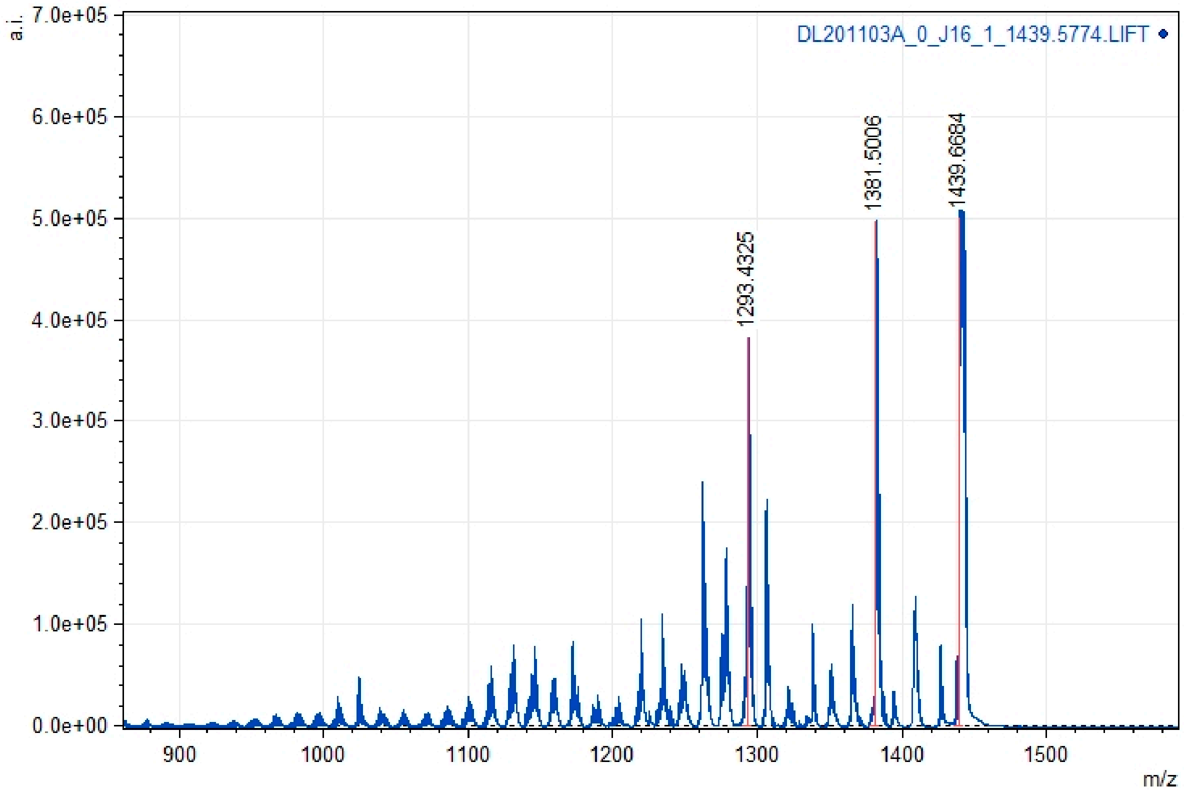

Figure S41. Fragmentation mass spectra of porphyrin **7**.

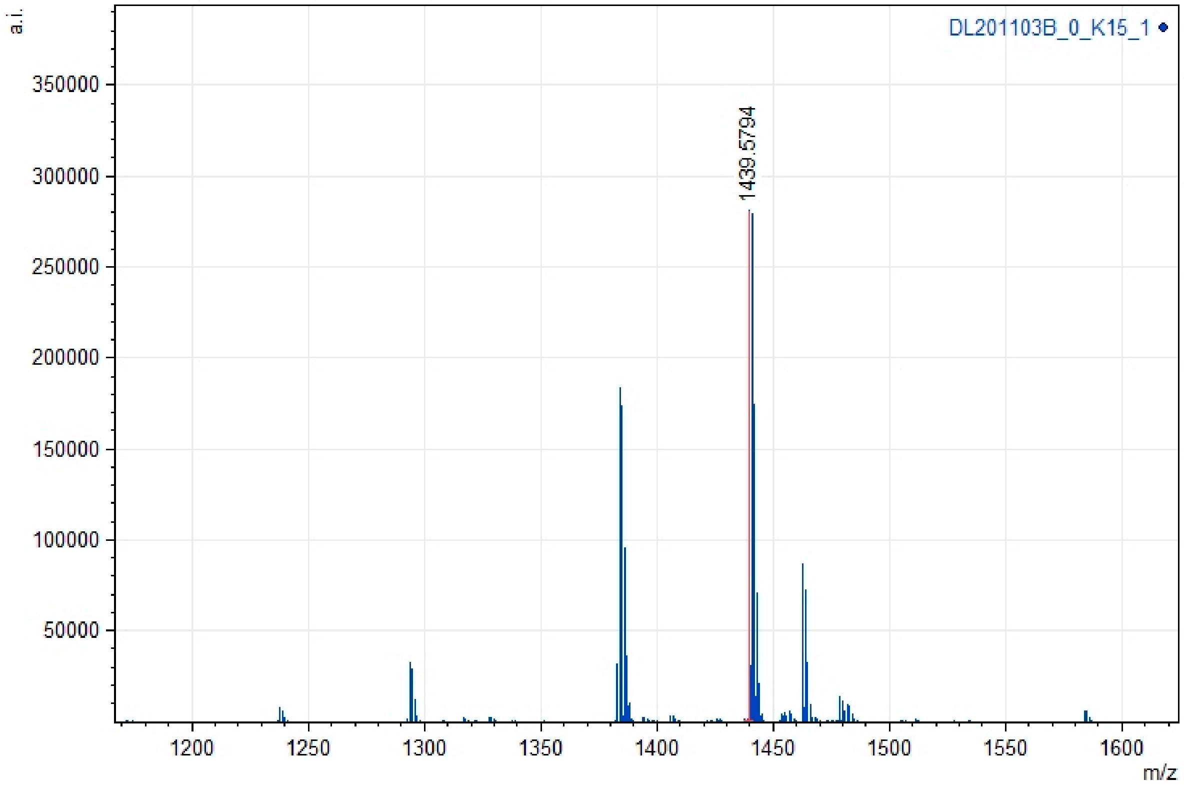

Figure S42. Mass spectra of porphyrin **8**.

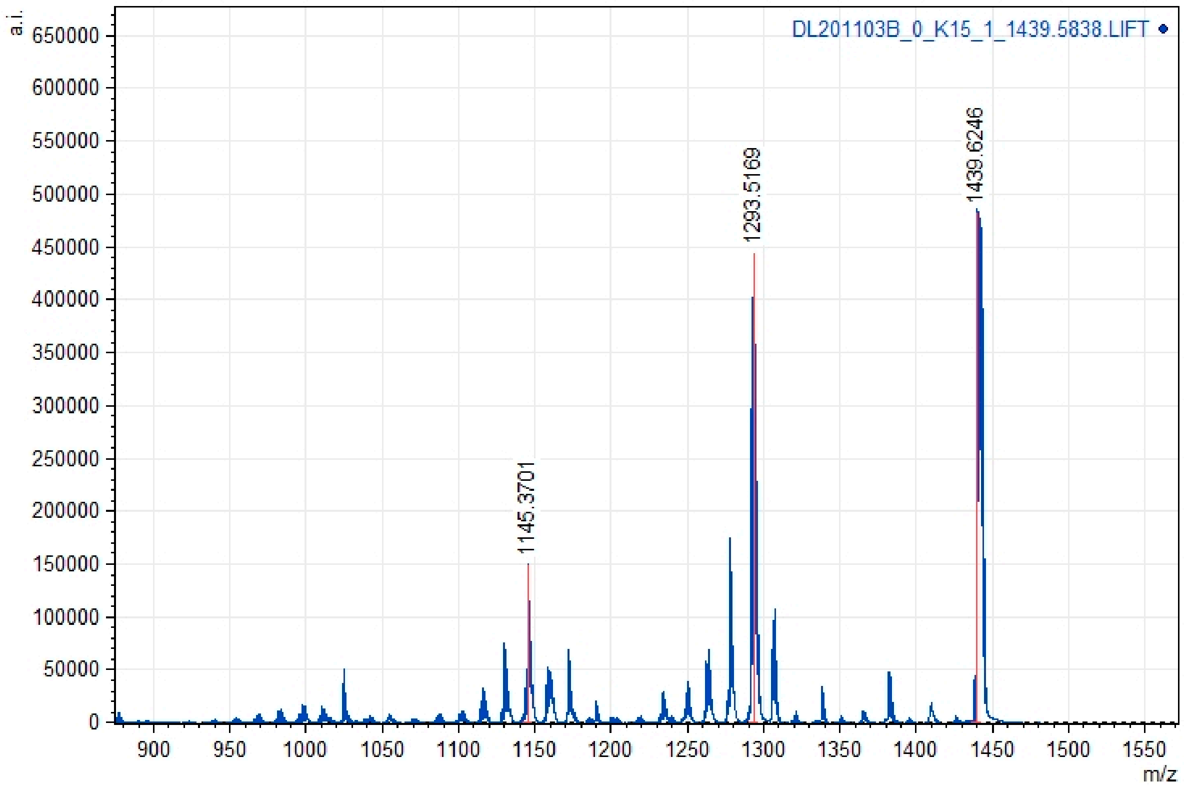

Figure S43. Fragmentation mass spectra of porphyrin **8**.

3. IR data

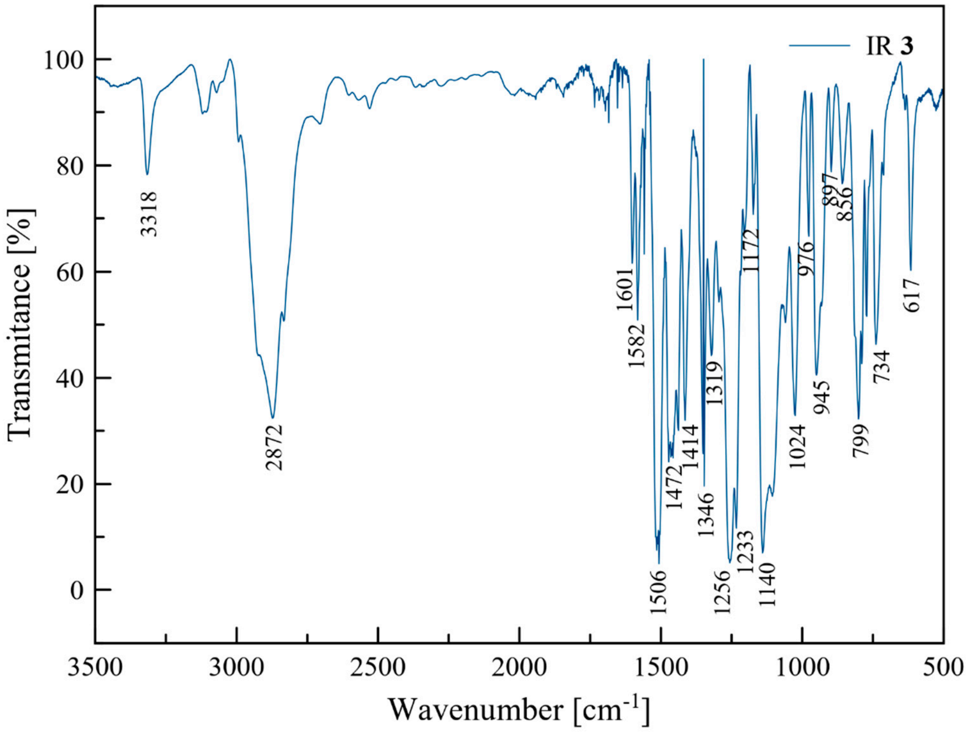

Figure S44. IR spectrum of porphyrin **3.**

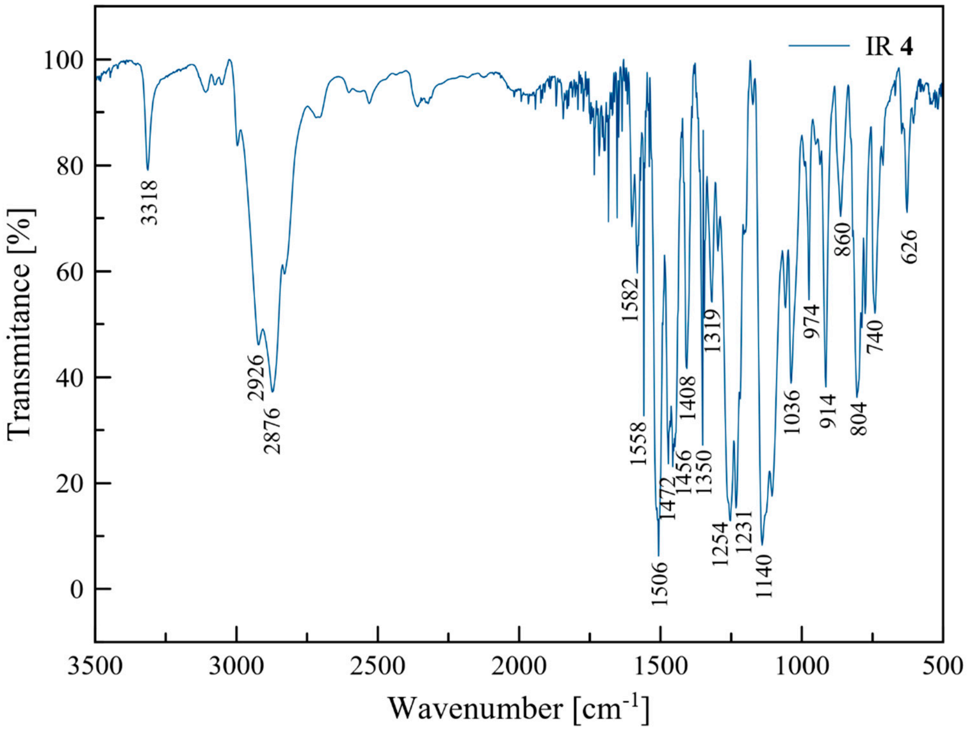

Figure S45. IR spectrum of porphyrin **4.**

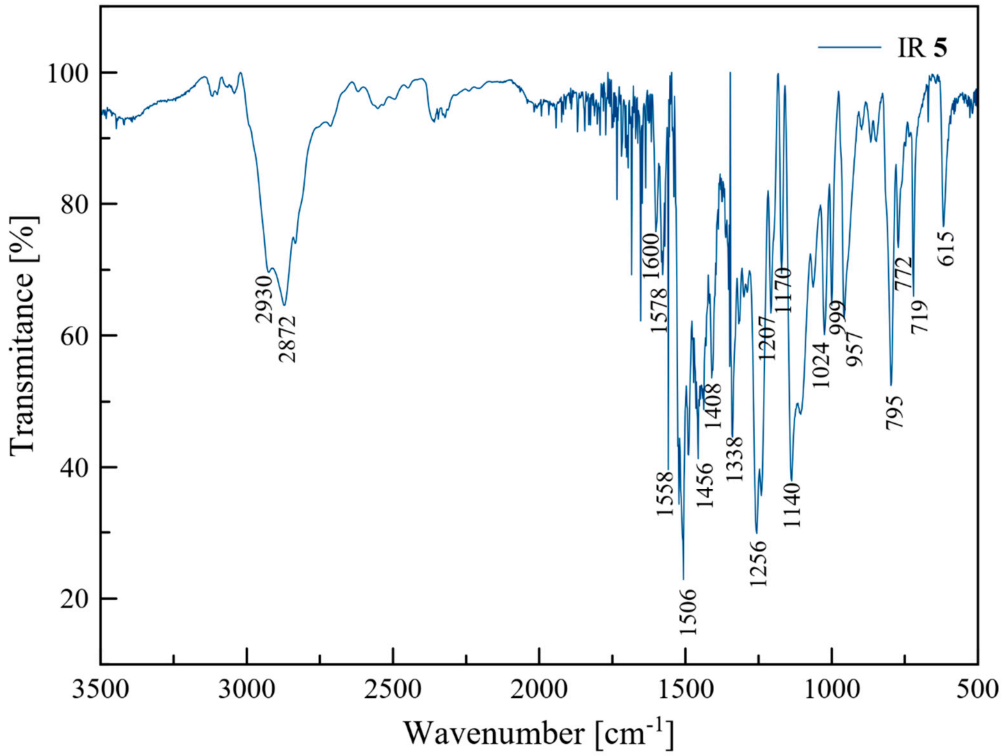

Figure S46. IR spectrum of porphyrin **5**.

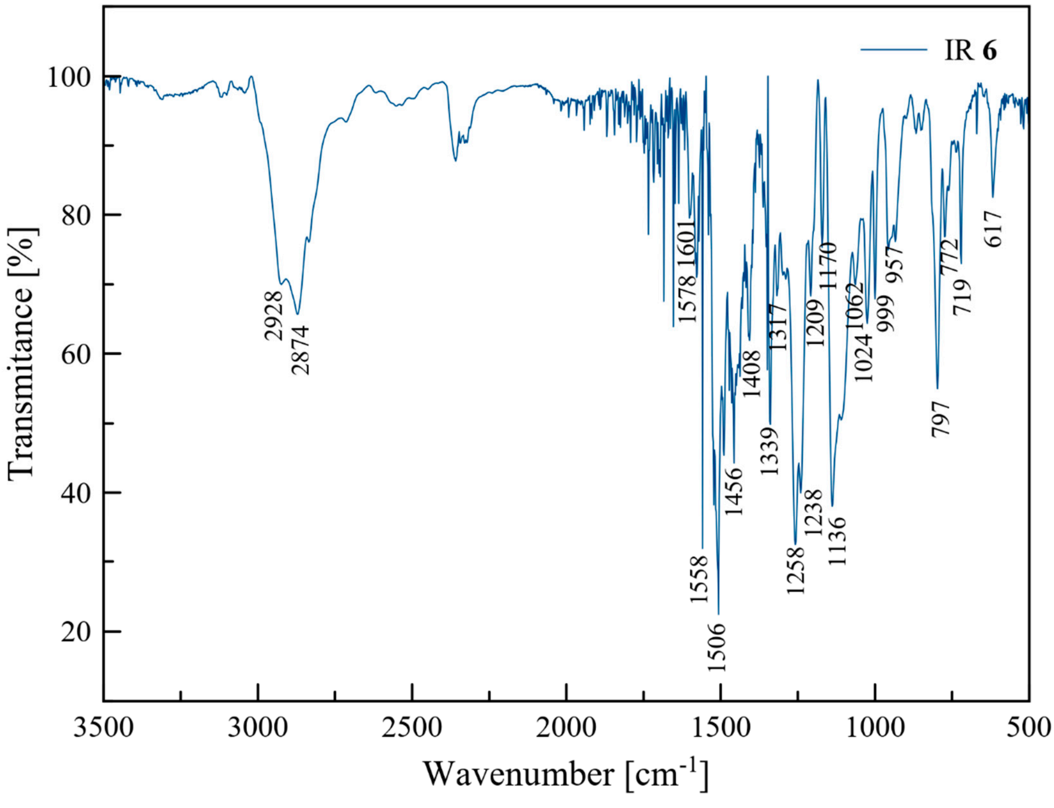

Figure S47. IR spectrum of porphyrin **6**.

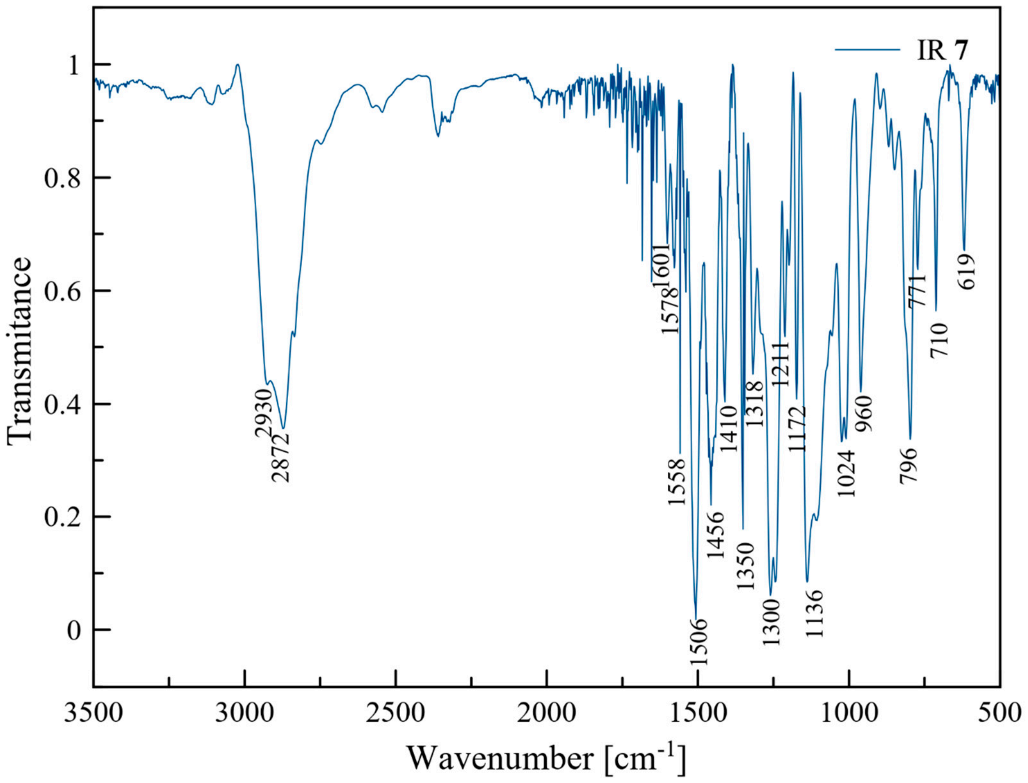

Figure S48. IR spectrum of porphyrin **7.**

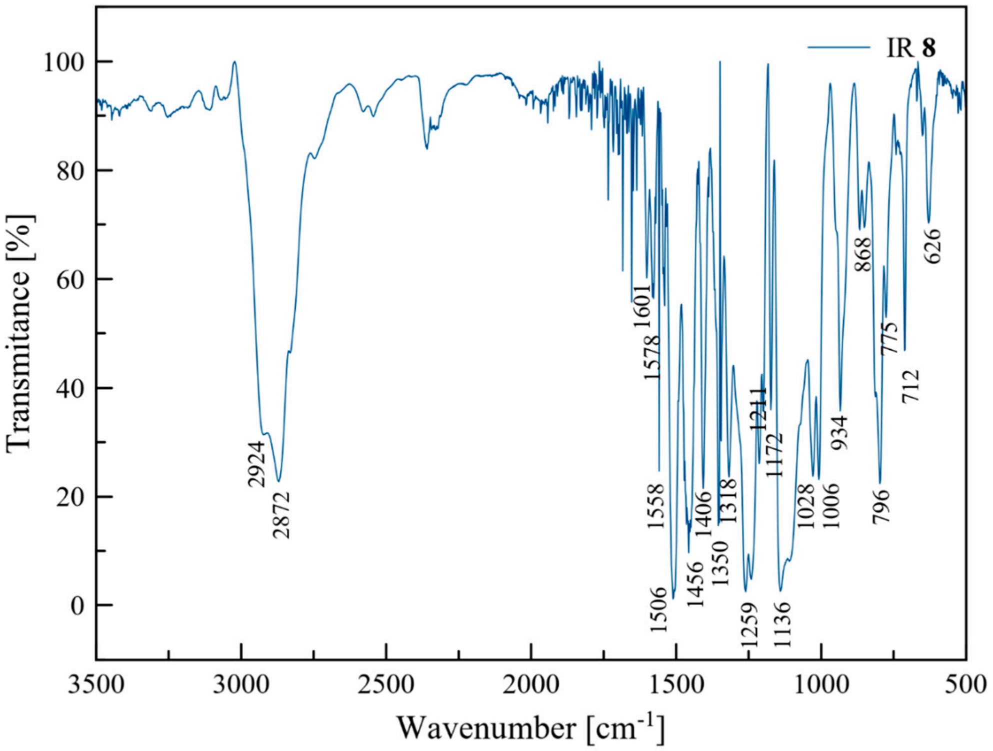

Figure S49. IR spectrum of porphyrin **8.**

4. UV-vis data

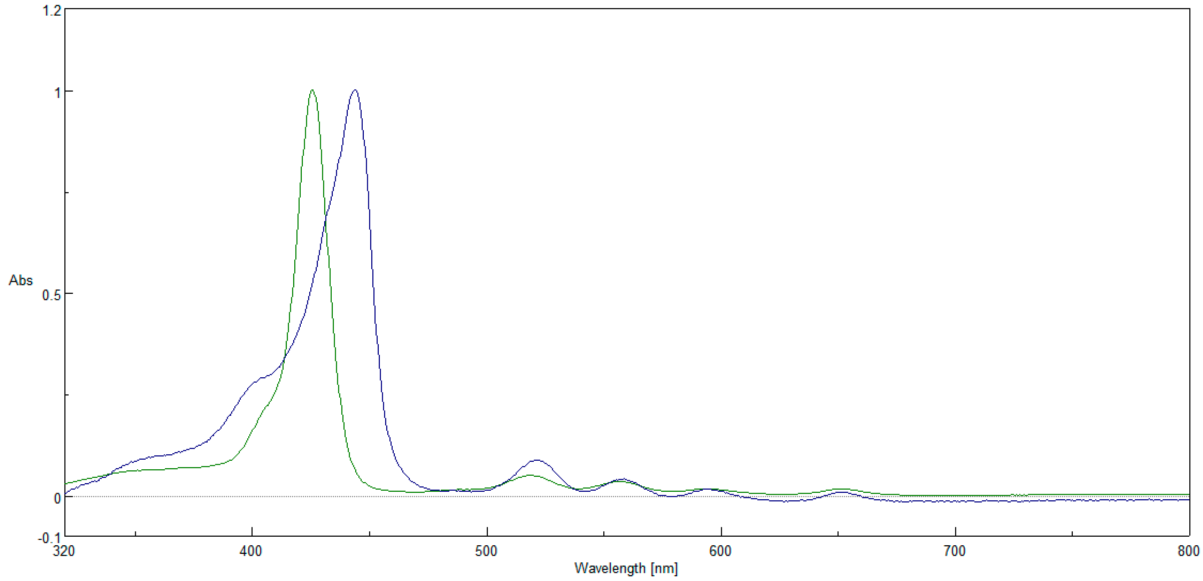

Figure S50. UV-vis spectrum of porphyrin **3** normalized in DMF (green) and water (blue).

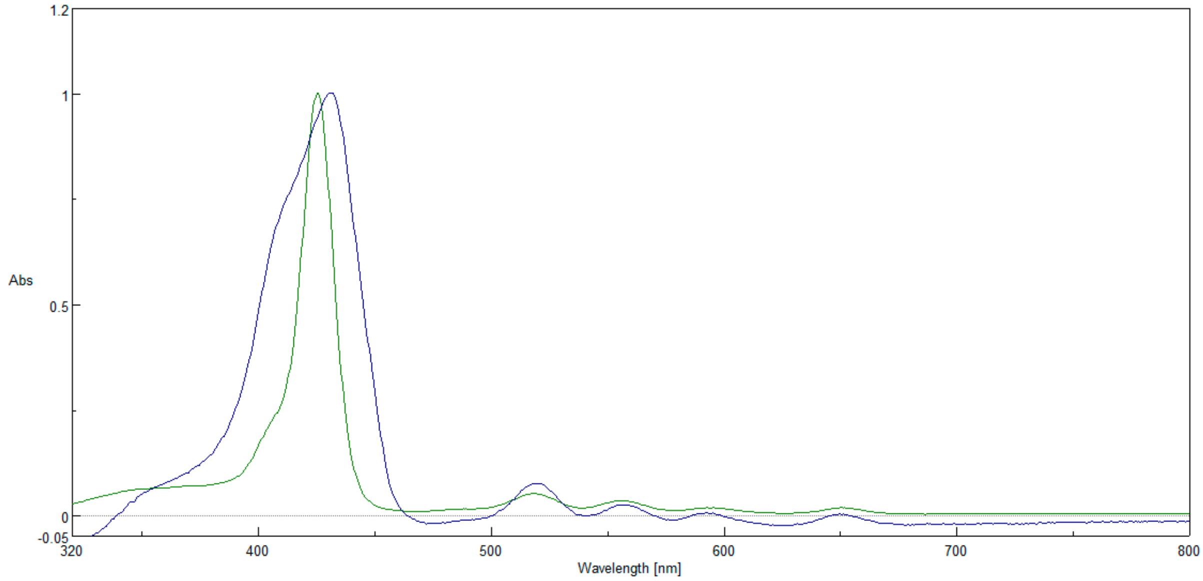

Figure S51. UV-vis spectrum of porphyrin **4** normalized in DMF (green) and water (blue).

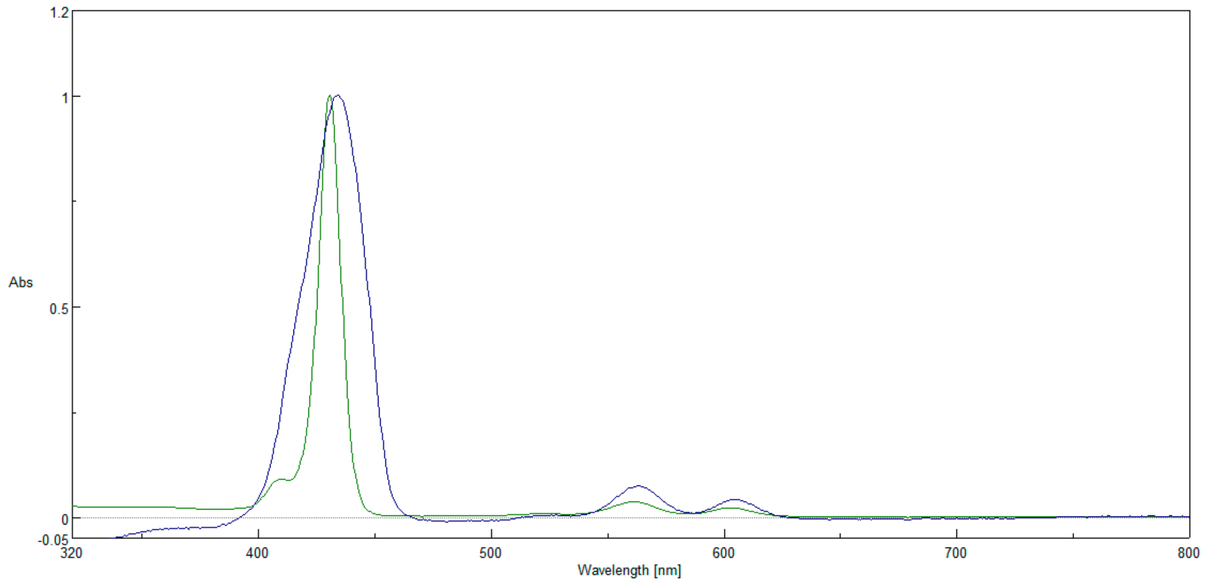

Figure S52. UV-vis spectrum of porphyrin **5** normalized in DMF (green) and water (blue).

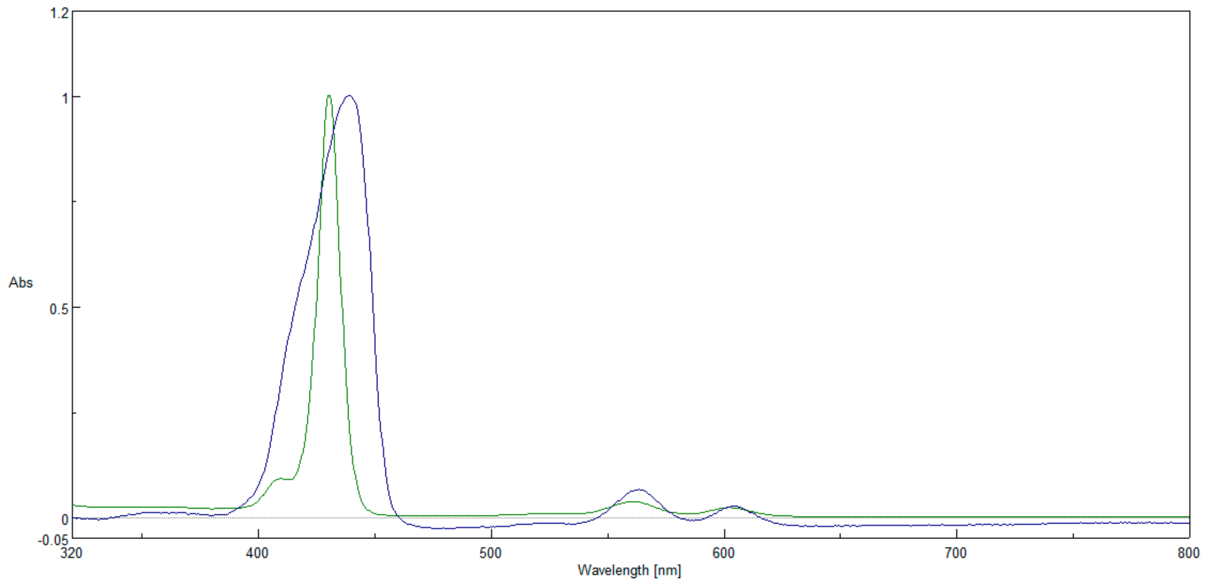

Figure S53. UV-vis spectrum of porphyrin **6** normalized in DMF (green) and water (blue).

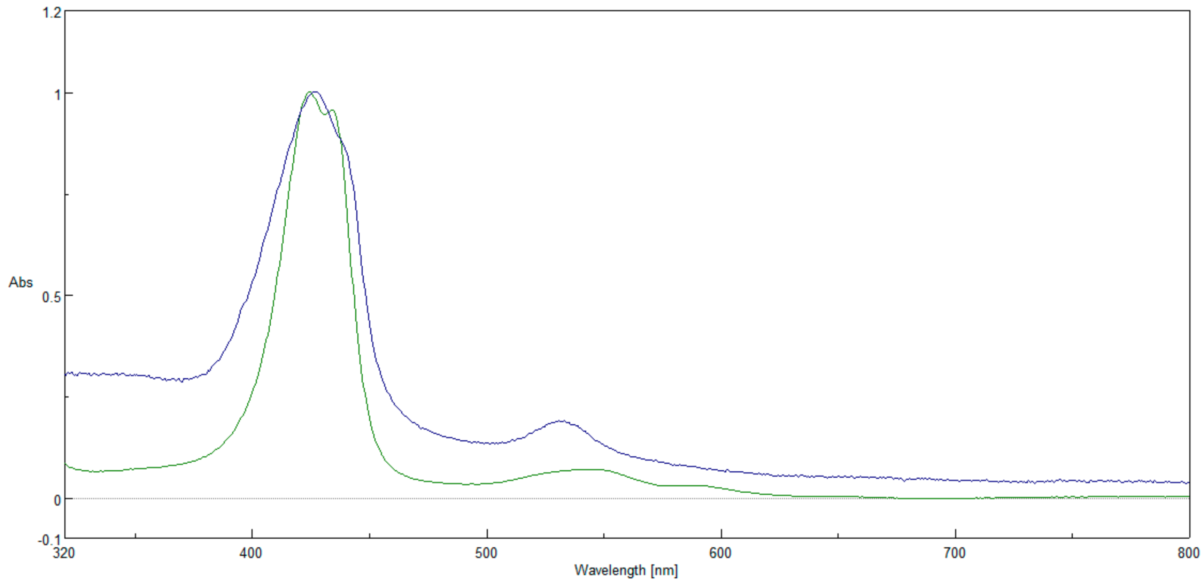

Figure S54. UV-vis spectrum of porphyrin **7** normalized in DMF (green) and water (blue).

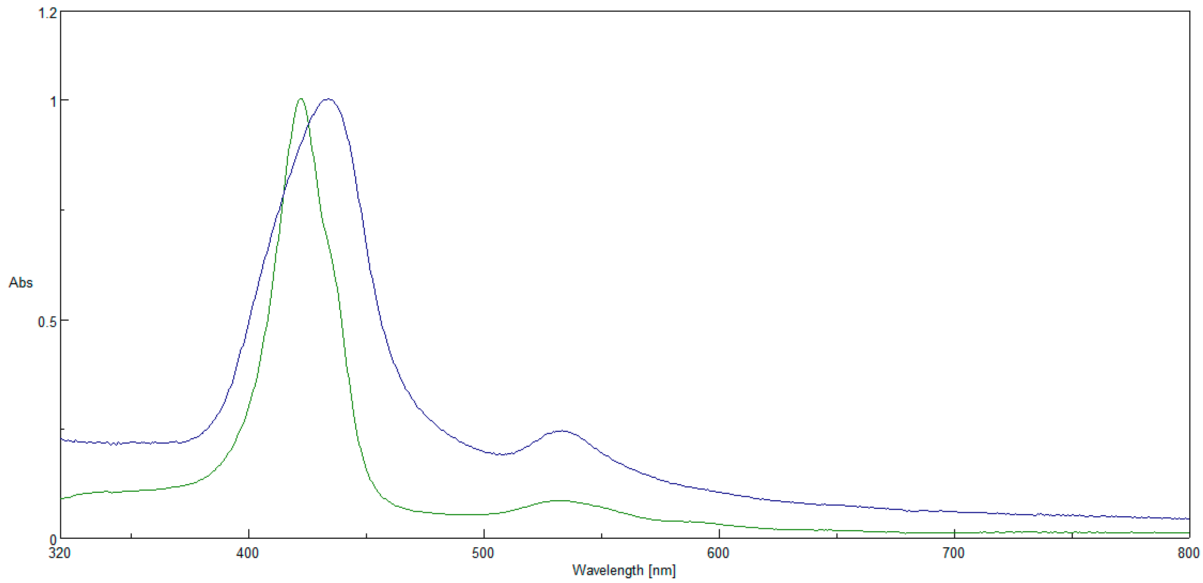

Figure S55. UV-vis spectrum of porphyrin **8** normalized in DMF (green) and water (blue).

4 HPLC data

Chromatograph HPLC: Agilent 1260 Infinity II

Column: Luna 5 µm C18(2) 100 Å 150 x 4.6 mm, Phenomenex

Solvent: Acetonitrile

Column temp.: 35°C

Mobile phase: A – H_2O, B – acetonitrile

Table S1. Gradient 3.

**Time, min**	**A**	**B**
0	50	50
5	40	60
10	30	70
20	0	100
30	50	50

Flow: 1.0 ml/min

Termostat: 20°C

Injection: 10 µl

Analysis time: 30 min

Detector DAD, ELSD

Table S2. Analysis date: 13.12.2021

Symbol	C, mg/ml	λ_{max}, nm	Method		
			t_R, min	%	ELSD, %
3	0.2	424	17.71	96.78	96.52
4	0.2	423	16.95	95.30	95.12
5	0.2	426	14.94	96.72	96.49
6	0.2	425	15.20	96.00	96.00
7	0.2	436	16.70	96.12	96.02
8	0.2	436	16.02	96.10	96.05

6 Computational data

The first twenty vertical excitations of **3** at the PCM/TD-DFT/PBE1PBE/6-31+G(d,p)// PBE1PBE/6-31G(d,p) of theory.

Excited state 1 (E=2.0952 eV, oscillator strength f= 0.106) the transitions: 369 -> 370   0.85 (73%,) 368 -> 371   0.49 (24%)

Excited state 2 (E=2.2234 eV, oscillator strength f=0.157) the transitions: 369 -> 371   0.85 (73%), 368 -> 370 -0.50 (25%)

Excited state 3 (E=2.8783 eV, oscillator strength f=1.023) the transitions: 368 -> 371   0.65 (43%), 367 -> 371   0.46 (21%), 369 -> 370 -0.38 (15%), 364 -> 370 -0.38 (15%), 368 -> 370 -0.20 (4%)

Excited state 4 (E=2.9093 eV, oscillator strength f=1.061) the transitions: 368 -> 370   0.61 (37%), 367 -> 370 -0.51 (26%), 364 -> 371   0.40 (16%), 369 -> 371   0.38 (14%), 368 -> 371   0.21 (4%)

Excited state 5 (E=2.9474 eV, oscillator strength f=0.000) the transitions: 365 -> 370   0.69 (47%), 366 -> 371   0.58 (34%), 366 -> 370 -0.39 (15%), 365 -> 371 -0.15 (2%)

Excited state 6 (E=2.9528 eV, oscillator strength f=0.000) the transitions: 366 -> 370   0.78 (61%), 366 -> 371   0.41 (17%), 365 -> 371   0.41 (17%), 365 -> 370   0.19 (3%)

Excited state 7 (E=2.9591 eV, oscillator strength f=0.191) the transitions: 367 -> 370   0.84 (70%), 368 -> 370   0.42 (18%), 369 -> 371   0.23 (5%), 364 -> 371   0.16 (3%), 367 -> 371   0.15 (2%)

Excited state 8 (E=3.0029 eV, oscillator strength f=0.000) the transitions: 365 -> 370   0.69 (48%), 366 -> 371 -0.68 (46%), 366 -> 370   0.22 (5%)

Excited state 9 (E=3.0099 eV, oscillator strength f=0.160) the transitions: 367 -> 371   0.87 (75%), 368 -> 371 -0.30 (9%), 364 -> 370   0.25 (6%), 369 -> 370   0.23 (5%), 367 -> 370 -0.15 (2%)

Excited state 10 (E=3.0301 eV, oscillator strength f=0.002) the transitions: 365 -> 371   0.89 (80%) 366 -> 370 -0.42 (17%)

Excited state 11 (E=3.2612 eV, oscillator strength f=0.405) the transitions: 364 -> 370   0.84 (70%), 368 -> 371   0.37 (14%), 362 -> 370 -0.24 (6%), 358 -> 370 -0.18 (3%), 369 -> 370 -0.15 (2%)

Excited state 12 (E=3.3446 eV, oscillator strength f=0.554) the transitions: 364 -> 371   0.88 (78%), 368 -> 370 -0.36 (13%), 369 -> 371 -0.21 (4%)

Excited state 13 (E=3.4633 eV, oscillator strength f=0.000) the transitions: 363 -> 370   0.90 (81%), 360 -> 370 -0.37 (14%)

Excited state 14 (E=3.6407 eV, oscillator strength f=0.413) the transitions: 362 -> 370 0.84 (71%), 359 -> 370 0.32 (10%), 364 -> 370 0.23 (5%), 358 -> 370 0.21 (4%), 362 -> 371 -0.17 (3%), 368 -> 371 0.15 (2%)

Excited state 15 (E=3.6864 eV, oscillator strength f=0.000) the transitions: 363 -> 371 0.97 (94%), 360 -> 371 -0.18 (3%)

Excited state 16 (E=3.7131 eV, oscillator strength f=0.039) the transitions: 362 -> 371 0.94 (88%), 358 -> 371 0.18 (3%), 359 -> 371 0.17 (3%)

Excited state 17 (E=3.8032 eV, oscillator strength f=0.001) the transitions: 361 -> 370 0.61 (37%), 361 -> 371 -0.57 (32%), 360 -> 370 0.37 (14%), 369 -> 372 0.27 (7%), 363 -> 370 0.25 (6%)

Excited state 18 (E=3.8148 eV, oscillator strength f=0.000) the transitions: 369 -> 372 0.89 (80%), 361 -> 370 -0.33 (11%), 360 -> 371 0.24 (6%)

Excited state 19 (E=3.8292 eV, oscillator strength f=0.001) the transitions: 360 -> 370 0.63 (40%), 360 -> 371 0.54 (29%), 361 -> 370 -0.44 (19%), 369 -> 372 -0.25 (6%), 363 -> 370 0.20 (4%)

Excited state 20 (E=3.8515 eV, oscillator strength f=0.023) the transitions: 359 -> 370 0.74 (55%), 359 -> 371 0.41 (17%), 362 -> 370 -0.40 (16%), 358 -> 370 0.31 (10%)

The first twenty vertical excitations of **4** at the PCM/TD-DFT/PBE1PBE/6-31+G(d,p)// PBE1PBE/6-31G(d,p) of theory.

Excited state 1 (E=2.0787 eV, oscillator strength f=0.150) the transitions: 369 -> 370 0,87 (76%), 368 -> 371 -0,47 (22%)

Excited state 2 (E=2.1992 eV, oscillator strength f=0.227) the transitions: 369 -> 371 0,88 (77%), 368 -> 370 0,47 (22%)

Excited state 3 (E=2.8914 eV, oscillator strength f=1.177) the transitions: 368 -> 371 0,70 (48%), 366 -> 371 0,43 (19%), 369 -> 370 0,37 (14%), 364 -> 370 0,35 (12%), 368 -> 370 -0,21 (4%)

Excited state 4 (E=2.9281 eV, oscillator strength f=1.273) the transitions: 368 -> 370 0,69 (48%), 366 -> 370 -0,40 (16%), 369 -> 371 -0,38 (15%), 364 -> 371 -0,38 (14%), 368 -> 371 0,22 (5%)

Excited state 5 (E=2.9719 eV, oscillator strength f=0.001) the transitions: 367 -> 371 0,65 (42%), 365 -> 370 -0,65 (42%), 367 -> 370 -0,36 (13%)

Excited state 6 (E=2.9784 eV, oscillator strength f=0.000) the transitions: 367 -> 370 0,82 (67%), 367 -> 371 0,39 (15%), 365 -> 371 -0,39 (15%)

Excited state 7 (E=2.9787 eV, oscillator strength f=0.094) the transitions: 367 -> 370 0,82 (67%), 367 -> 371 0,39 (15%), 365 -> 371 -0,39 (15%)

Excited state 8 (E=3.0203 eV, oscillator strength f=0.000) the transitions: 365 -> 370 0,74 (55%), 367 -> 371 0,63 (40%), 367 -> 370 -0,20 (4%)

Excited state 9 (E=3.0344 eV, oscillator strength f=0.141) the transitions: 366 -> 371 0,88 (78%), 368 -> 371 -0,30 (9%), 364 -> 370 -0,23 (5%), 369 -> 370 -0,22 (5%)

Excited state 10 (E=3.0537 eV, oscillator strength f=0.000) the transitions: 365 -> 371 0,91 (83%), 367 -> 370 0,39 (15%)

Excited state 11 (E=3.3119 eV, oscillator strength f=0.353) the transitions: 364 -> 370 0,86 (73%), 368 -> 371 -0,34 (11%), 362 -> 370 -0,25 (6%), 358 -> 370 0,17 (3%)

Excited state 12 (E=3.3924 eV, oscillator strength f=0.473) the transitions: 364 -> 371 0,91 (82%), 368 -> 370 0,33 (11%), 369 -> 371 -0,17 (3%)

Excited state 13 (E=3.5034 eV, oscillator strength f=0.000) the transitions: 363 -> 370 0,88 (78%), 360 -> 370 0,39 (16%), 369 -> 372 -0,15 (2%)

Excited state 14 (E=3.6611 eV, oscillator strength f=0.371) the transitions: 362 -> 370 0,85 (72%), 359 -> 370 0,33 (11%), 364 -> 370 0,24 (6%), 362 -> 371 -0,18 (3%), 358 -> 370 -0,16 (3%)

Excited state 15 (E=3.7156 eV, oscillator strength f=0.000) the transitions: 363 -> 371 0,97 (93%),360 -> 371 0,19 (4%)

Excited state 16 (E=3.7378 eV, oscillator strength f=0.032) the transitions: 362 -> 371 0,94 (89%),359 -> 371 0,18 (3%), 358 -> 371 -0,15 (2%)

Excited state 17 (E=3.7601 eV, oscillator strength f=0.000) the transitions: 369 -> 372 0,97 (94%)

Excited state 18 (E=3.8339 eV, oscillator strength f=0.000) the transitions: 361 -> 370 0,65 (43%), 361 -> 371 -0,57 (33%), 360 -> 370 -0,40 (16%), 363 -> 370 0,25 (6%)

Excited state 19 (E=3.8635 eV, oscillator strength f=0.000) the transitions: 360 -> 370 0,61 (37%), 360 -> 371 0,57 (33%), 361 -> 370 0,48 (23%), 363 -> 370 -0,21 (4%)

Excited state 20 (E=3.8904 eV, oscillator strength f=0.032) the transitions: 359 -> 370 0,79 (62%), 362 -> 370 -0,39 (15%), 359 -> 371 0,38 (14%), 358 -> 370 -0,24 (6%)

The first twenty vertical excitations of **5** at the PCM/TD-DFT/PBE1PBE/6-31+G(d,p)// PBE1PBE/6-31G(d,p) of theory.

Excited state 1 (E=2.2021 eV, oscillator strength f=0.139) the transitions: 383 -> 384 0.84 (71%), 382 -> 385 -0.49 (24%), 383 -> 385 -0.18 (3%)

Excited state 2 (E=2.2021 eV, oscillator strength f=0.139) the transitions: 383 -> 385 0,84 (71%), 382 -> 384 0,49 (24%), 383 -> 384 0,18 (3%)

Excited state 3 (E=2.9538 eV, oscillator strength f=1.429) the transitions: 382 -> 385 0,62 (38%), 382 -> 384 -0,51 (26%), 383 -> 384 0,37 (14%), 383 -> 385 0,29 (8%), 378 -> 384 0,26 (7%), 381 -> 385 0,22 (5%)

Excited state 4 (E=2.9538 eV, oscillator strength f=1.429) the transitions: 382 -> 384 0,62 (38%), 382 -> 385 0,51 (26%), 383 -> 385 -0,37 (14%), 383 -> 384 0,29 (8%), 378 -> 385 -0,26 (7%), 381 -> 384 -0,22 (5%)

Excited state 5 (E=3.1049 eV, oscillator strength f=0.001) the transitions: 379 -> 384 0,67 (46%), 380 -> 385 0,67 (46%), 379 -> 385 -0,19 (4%), 380 -> 384 0,19 (4%)

Excited state 6 (E=3.1168 eV, oscillator strength f=0.000) the transitions: 380 -> 384 0,53 (28%), 379 -> 385 0,53 (28%), 380 -> 385 0,46 (21%), 379 -> 384 -0,46 (21%)

Excited state 7 (E=3.1289 eV, oscillator strength f=0.031) the transitions: 381 -> 384 0,95 (91%), 381 -> 385 -0,18 (3%), 382 -> 384 0,15 (2%)

Excited state 8 (E=3.1289 eV, oscillator strength f=0.031) the transitions: 381 -> 385 0,95 (91%), 381 -> 384 0,18 (3%), 382 -> 385 -0,15 (2%)

Excited state 9 (E=3.1538 eV, oscillator strength f=0.000) the transitions: 380 -> 385 0,53 (28%), 379 -> 384 -0,53 (28%), 380 -> 384 -0,46 (21%), 379 -> 385 -0,46 (21%)

Excited state 10 (E=3.1792 eV, oscillator strength f=0.001) the transitions: 379 -> 385 0,68 (46%), 380 -> 384 -0,68 (46%), 379 -> 384 0,19 (4%), 380 -> 385 0,19 (4%)

Excited state 11 (E=3.4021 eV, oscillator strength f=0.086) the transitions: 378 -> 384 0,81 (65%), 376 -> 384 -0,52 (27%), 382 -> 385 -0,18 (3%)

Excited state 12 (E=3.4021 eV, oscillator strength f=0.086) the transitions: 378 -> 385 0,81 (65%), 376 -> 385 -0,52 (27%), 382 -> 384 0,18 (3%)

Excited state 13 (E=3.6027 eV, oscillator strength f=0.268) the transitions: 376 -> 384 0,82 (68%), 378 -> 384 0,46 (21%), 382 -> 385 -0,19 (4%)

Excited state 14 (E=3.6027 eV, oscillator strength f=0.268) the transitions: 376 -> 385 0,82 (68%), 378 -> 385 0,46 (21%), 382 -> 384 0,19 (4%)

Excited state 15 (E=3.7907 eV, oscillator strength f=0.000) the transitions: 374 -> 384 0,48 (23%), 375 -> 385 0,48 (23%), 372 -> 384 0,37 (14%), 373 -> 385 0,37 (14%), 373 -> 384 0,30 (9%), 372 -> 385 -0,30 (9%), 375 -> 384 0,18 (3%), 374 -> 385 -0,18 (3%)

Excited state 16 (E=3.7975 eV, oscillator strength f=0.000) the transitions: 383 -> 386 0,83 (70%), 375 -> 385 -0,24 (6%), 374 -> 384 0,24 (6%), 372 -> 385 0,21 (5%), 373 -> 384 0,21 (5%)

E xcited state 17 (E=3.8278 eV, oscillator strength f=0.047) the transitions: 377 -> 384 0,89 (79%), 370 -> 384 -0,35 (12%), 377 -> 385 -0,19 (4%)

Excited state 18 (E=3.8279 eV, oscillator strength f=0.047) the transitions: 377 -> 385 0,89 (79%), 370 -> 385 -0,35 (12%), 377 -> 384 0,19 (4%)

Excited state 19 (E=3.8391 eV, oscillator strength f=0.000) the transitions: 383 -> 386 0,52 (27%), 372 -> 385 -0,33 (11%), 373 -> 384 -0,33 (11%), 375 -> 385 0,32 (10%), 374 -> 384 -0,32 (10%), 375 -> 384 -0,29 (9%), 374 -> 385 -0,29 (9%), 373 -> 385 0,25 (6%), 372 -> 384 -0,25 (6%)

Excited state 20 (E=3.9223 eV, oscillator strength f=0.001) the transitions: 375 -> 384 0,57 (32%), 374 -> 385 0,57 (32%), 372 -> 384 -0,29 (8%), 373 -> 385 0,29 (8%), 374 -> 384 -0,28 (8%), 375 -> 385 0,28 (8%)

Atomic coordinates of global minimum of compound 3

6	-3.471933000	-0.029052000	0.026489000
6	-0.001281000	3.473278000	0.013832000
6	3.471933000	0.029052000	0.026489000
6	0.001281000	-3.473278000	0.013832000
7	1.434225000	1.447246000	-0.003665000
7	-1.501973000	1.490147000	0.037710000
7	-1.434225000	-1.447246000	-0.003665000
7	1.501973000	-1.490147000	0.037710000
6	-1.240094000	-2.799699000	0.088491000
6	-2.787915000	-1.264751000	0.096559000
6	-2.852840000	1.232528000	-0.041750000
6	-1.257411000	2.843073000	-0.049741000

6	1.257411000	-2.843073000	-0.049741000
6	2.852840000	-1.232528000	-0.041750000
6	2.787915000	1.264751000	0.096559000
6	1.240094000	2.799699000	0.088491000
6	-2.507003000	-3.493043000	0.289049000
6	-3.469459000	-2.538547000	0.294949000
6	-3.497539000	2.501579000	-0.210586000
6	-2.533355000	3.474955000	-0.215920000
6	2.507003000	3.493043000	0.289049000
6	3.469459000	2.538547000	0.294949000
6	2.533355000	-3.474955000	-0.215920000
6	3.497539000	-2.501579000	-0.210586000
1	-2.629265000	-4.557044000	0.426167000
1	-4.531545000	-2.669913000	0.437332000
1	-4.561681000	2.637433000	-0.324679000
1	-2.679196000	4.537138000	-0.334702000
1	2.629265000	4.557044000	0.426167000
1	4.531545000	2.669913000	0.437332000
1	2.679196000	-4.537138000	-0.334702000
1	4.561681000	-2.637433000	-0.324679000
6	-4.968063000	-0.045804000	0.018699000
6	-5.677312000	-0.651703000	-1.019679000
6	-5.693341000	0.555221000	1.068400000
1	-5.141176000	-1.112558000	-1.842571000
1	-5.149431000	1.016785000	1.883084000
6	-7.077258000	-0.661446000	-1.023498000
6	-7.084724000	0.548452000	1.077728000
1	-7.600741000	-1.131839000	-1.846744000
6	-7.793471000	-0.068596000	0.014706000
6	0.001281000	4.969393000	-0.001635000
6	-0.598445000	5.706794000	1.020582000
6	0.615320000	5.666028000	-1.063260000
1	-1.067628000	5.193708000	1.853528000
1	1.073421000	5.099623000	-1.864250000

6	-0.587792000	7.106709000	0.998076000
6	0.628936000	7.056716000	-1.098613000
1	-1.052658000	7.652264000	1.810026000
6	0.018718000	7.794303000	-0.051293000
6	4.968063000	0.045804000	0.018699000
6	5.693341000	-0.555221000	1.068400000
6	5.677312000	0.651703000	-1.019679000
1	5.149431000	-1.016785000	1.883084000
1	5.141176000	1.112558000	-1.842571000
6	7.084724000	-0.548452000	1.077728000
6	7.077258000	0.661446000	-1.023498000
1	7.600741000	1.131839000	-1.846744000
6	7.793471000	0.068596000	0.014706000
6	-0.001281000	-4.969393000	-0.001635000
6	-0.615320000	-5.666028000	-1.063260000
6	0.598445000	-5.706794000	1.020582000
1	-1.073421000	-5.099623000	-1.864250000
1	1.067628000	-5.193708000	1.853528000
6	-0.628936000	-7.056716000	-1.098613000
6	0.587792000	-7.106709000	0.998076000
1	1.052658000	-7.652264000	1.810026000
6	-0.018718000	-7.794303000	-0.051293000
8	0.082696000	9.149606000	-0.169732000
6	-0.517962000	9.937808000	0.855589000
1	-1.594028000	9.743675000	0.931876000
1	-0.047747000	9.755497000	1.828735000
1	-0.357637000	10.976466000	0.565677000
8	-9.151636000	-0.025264000	0.107584000
6	-9.911095000	-0.635201000	-0.933801000
1	-9.717262000	-0.160038000	-1.902309000
1	-9.699471000	-1.708057000	-1.008588000
1	-10.957377000	-0.491027000	-0.663483000
8	-0.082696000	-9.149606000	-0.169732000
6	0.517962000	-9.937808000	0.855589000

1	1.594028000	-9.743675000	0.931876000
1	0.047747000	-9.755497000	1.828735000
1	0.357637000	-10.976466000	0.565677000
8	9.151636000	0.025264000	0.107584000
6	9.911095000	0.635201000	-0.933801000
1	9.717262000	0.160038000	-1.902309000
1	9.699471000	1.708057000	-1.008588000
1	10.957377000	0.491027000	-0.663483000
8	1.192954000	7.805202000	-2.091501000
6	1.832233000	7.127729000	-3.170572000
1	2.655339000	6.500850000	-2.805034000
1	1.120238000	6.487535000	-3.706584000
1	1.541353000	8.838866000	-4.456535000
6	2.368311000	8.205839000	-4.100769000
1	3.075650000	8.851238000	-3.558275000
8	3.004369000	7.543536000	-5.178904000
6	3.553912000	8.438338000	-6.132570000
1	4.307489000	9.093193000	-5.669141000
1	2.772097000	9.080993000	-6.565181000
6	4.198841000	7.600538000	-7.226577000
1	4.979453000	6.956587000	-6.793109000
1	3.444503000	6.945621000	-7.689207000
8	4.748322000	8.494797000	-8.178916000
6	5.386558000	7.832032000	-9.257737000
1	6.211254000	7.197770000	-8.897791000
1	4.677963000	7.184271000	-9.796500000
1	6.634216000	9.548922000	-9.656488000
6	5.927843000	8.899279000	-10.197324000
1	5.102073000	9.533826000	-10.556389000
8	6.566363000	8.236966000	-11.273649000
6	7.111229000	9.139815000	-12.220404000
1	6.333330000	9.775282000	-12.669017000
1	7.579180000	8.543465000	-13.006892000
1	7.871652000	9.792853000	-11.767051000

8	7.859610000	-1.097871000	2.058570000
6	7.211381000	-1.737445000	3.155189000
1	6.569829000	-1.030668000	3.696425000
1	6.591012000	-2.573392000	2.808109000
1	8.959704000	-2.951951000	3.523493000
6	8.313644000	-2.248925000	4.070804000
1	8.940584000	-1.409532000	4.407687000
8	7.679616000	-2.884463000	5.166167000
6	8.598738000	-3.409912000	6.110240000
1	9.236606000	-2.613812000	6.523443000
1	9.256993000	-4.158584000	5.643712000
6	7.789355000	-4.055287000	7.225085000
1	7.130224000	-3.306068000	7.690028000
1	7.150889000	-4.850776000	6.810949000
8	8.707541000	-4.580024000	8.168500000
6	8.072278000	-5.213377000	9.266597000
1	7.422616000	-4.507389000	9.806531000
1	7.444905000	-6.052340000	8.928158000
1	9.791025000	-4.885994000	10.532332000
6	9.162779000	-5.725849000	10.195543000
1	9.813689000	-6.430444000	9.653873000
8	8.527559000	-6.358460000	11.291521000
6	9.453460000	-6.876513000	12.231047000
1	10.110221000	-7.633811000	11.777853000
1	8.876807000	-7.341709000	13.033690000
1	10.084515000	-6.083086000	12.658174000
8	-7.859610000	1.097871000	2.058570000
6	-7.211381000	1.737445000	3.155189000
1	-6.569829000	1.030668000	3.696425000
1	-6.591012000	2.573392000	2.808109000
1	-8.959704000	2.951951000	3.523493000
6	-8.313644000	2.248925000	4.070804000
1	-8.940584000	1.409532000	4.407687000
8	-7.679616000	2.884463000	5.166167000

6	-8.598738000	3.409912000	6.110240000
1	-9.236606000	2.613812000	6.523443000
1	-9.256993000	4.158584000	5.643712000
6	-7.789355000	4.055287000	7.225085000
1	-7.130224000	3.306068000	7.690028000
1	-7.150889000	4.850776000	6.810949000
8	-8.707541000	4.580024000	8.168500000
6	-8.072278000	5.213377000	9.266597000
1	-7.422616000	4.507389000	9.806531000
1	-7.444905000	6.052340000	8.928158000
1	-9.791025000	4.885994000	10.532332000
6	-9.162779000	5.725849000	10.195543000
1	-9.813689000	6.430444000	9.653873000
8	-8.527559000	6.358460000	11.291521000
6	-9.453460000	6.876513000	12.231047000
1	-10.110221000	7.633811000	11.777853000
1	-8.876807000	7.341709000	13.033690000
1	-10.084515000	6.083086000	12.658174000
8	-1.192954000	-7.805202000	-2.091501000
6	-1.832233000	-7.127729000	-3.170572000
1	-2.655339000	-6.500850000	-2.805034000
1	-1.120238000	-6.487535000	-3.706584000
1	-1.541353000	-8.838866000	-4.456535000
6	-2.368311000	-8.205839000	-4.100769000
1	-3.075650000	-8.851238000	-3.558275000
8	-3.004369000	-7.543536000	-5.178904000
6	-3.553912000	-8.438338000	-6.132570000
1	-4.307489000	-9.093193000	-5.669141000
1	-2.772097000	-9.080993000	-6.565181000
6	-4.198841000	-7.600538000	-7.226577000
1	-4.979453000	-6.956587000	-6.793109000
1	-3.444503000	-6.945621000	-7.689207000
8	-4.748322000	-8.494797000	-8.178916000
6	-5.386558000	-7.832032000	-9.257737000

1	-6.211254000	-7.197770000	-8.897791000
1	-4.677963000	-7.184271000	-9.796500000
1	-6.634216000	-9.548922000	-9.656488000
6	-5.927843000	-8.899279000	-10.197324000
1	-5.102073000	-9.533826000	-10.556389000
8	-6.566363000	-8.236966000	-11.273649000
6	-7.111229000	-9.139815000	-12.220404000
1	-6.333330000	-9.775282000	-12.669017000
1	-7.579180000	-8.543465000	-13.006892000
1	-7.871652000	-9.792853000	-11.767051000
1	-0.783021000	0.778860000	0.102662000
1	0.783021000	-0.778860000	0.102662000

Atomic coordinates of global minimum of compound 4

6	1.398225000	3.160136000	-0.095781000
6	-3.170594000	1.374192000	-0.080152000
6	-1.398225000	-3.160136000	-0.095781000
6	3.170594000	-1.374192000	-0.080152000
7	-1.884652000	-0.736715000	-0.070236000
7	-0.767087000	1.962532000	-0.099022000
7	1.884652000	0.736715000	-0.070236000
7	0.767087000	-1.962532000	-0.099022000
6	3.041628000	0.025528000	-0.183967000
6	2.253033000	2.043169000	-0.191302000
6	0.000942000	3.092057000	0.000008000
6	-2.096831000	2.272080000	0.007605000
6	2.096831000	-2.272080000	0.007605000
6	-0.000942000	-3.092057000	0.000008000
6	-2.253033000	-2.043169000	-0.191302000
6	-3.041628000	-0.025528000	-0.183967000
6	4.173077000	0.903037000	-0.431248000

6	3.680297000	2.163801000	-0.436040000
6	-0.899160000	4.181504000	0.212543000
6	-2.172758000	3.683664000	0.217376000
6	-4.173077000	-0.903037000	-0.431248000
6	-3.680297000	-2.163801000	-0.436040000
6	2.172758000	-3.683664000	0.217376000
6	0.899160000	-4.181504000	0.212543000
1	5.192136000	0.585979000	-0.600425000
1	4.215778000	3.086317000	-0.609523000
1	-0.594839000	5.207289000	0.356899000
1	-3.090671000	4.232575000	0.365938000
1	-5.192136000	-0.585979000	-0.600425000
1	-4.215778000	-3.086317000	-0.609523000
1	3.090671000	-4.232575000	0.365938000
1	0.594839000	-5.207289000	0.356899000
6	2.004262000	4.516235000	-0.087096000
6	2.940885000	4.860412000	0.903725000
6	1.664995000	5.465969000	-1.046807000
1	3.196477000	4.123813000	1.656306000
1	0.953394000	5.214183000	-1.826714000
6	3.525453000	6.118522000	0.931227000
6	2.244406000	6.736239000	-1.029025000
1	1.970010000	7.454841000	-1.792622000
6	3.170594000	7.074621000	-0.050026000
6	-4.536457000	1.957506000	-0.057264000
6	-4.923309000	2.895736000	-1.030630000
6	-5.454775000	1.592342000	0.923273000
1	-4.213086000	3.169779000	-1.801970000
1	-5.169809000	0.876620000	1.687688000
6	-6.193176000	3.455668000	-1.021672000
6	-6.735685000	2.147088000	0.942615000

1	-7.429416000	1.851626000	1.721197000
6	-7.116625000	3.074874000	-0.019244000
6	-2.004262000	-4.516235000	-0.087096000
6	-1.664995000	-5.465969000	-1.046807000
6	-2.940885000	-4.860412000	0.903725000
1	-0.953394000	-5.214183000	-1.826714000
1	-3.196477000	-4.123813000	1.656306000
6	-2.244406000	-6.736239000	-1.029025000
6	-3.525453000	-6.118522000	0.931227000
1	-1.970010000	-7.454841000	-1.792622000
6	-3.170594000	-7.074621000	-0.050026000
6	4.536457000	-1.957506000	-0.057264000
6	5.454775000	-1.592342000	0.923273000
6	4.923309000	-2.895736000	-1.030630000
1	5.169809000	-0.876620000	1.687688000
1	4.213086000	-3.169779000	-1.801970000
6	6.735685000	-2.147088000	0.942615000
6	6.193176000	-3.455668000	-1.021672000
1	7.429416000	-1.851626000	1.721197000
6	7.116625000	-3.074874000	-0.019244000
8	6.648855000	-4.360006000	-1.920009000
6	5.750551000	-4.789296000	-2.925761000
1	4.861768000	-5.265398000	-2.493967000
1	5.437191000	-3.957296000	-3.567926000
1	6.295255000	-5.519522000	-3.524551000
8	-6.648855000	4.360006000	-1.920009000
6	-5.750551000	4.789296000	-2.925761000
1	-4.861768000	5.265398000	-2.493967000
1	-5.437191000	3.957296000	-3.567926000
1	-6.295255000	5.519522000	-3.524551000
8	4.431925000	6.533021000	1.847067000

6	4.836890000	5.600144000	2.831265000
1	3.994718000	5.286810000	3.460030000
1	5.297707000	4.713609000	2.378831000
1	5.574055000	6.114148000	3.448413000
8	-4.431925000	-6.533021000	1.847067000
6	-4.836890000	-5.600144000	2.831265000
1	-3.994718000	-5.286810000	3.460030000
1	-5.297707000	-4.713609000	2.378831000
1	-5.574055000	-6.114148000	3.448413000
8	8.330532000	-3.669810000	-0.089905000
6	9.294940000	-3.321955000	0.885177000
1	8.941300000	-3.570381000	1.894582000
1	9.519319000	-2.247676000	0.850926000
6	10.539140000	-4.121155000	0.561749000
1	10.886071000	-3.875561000	-0.454016000
1	10.307210000	-5.197338000	0.584664000
8	11.503394000	-3.784649000	1.527276000
6	12.722381000	-4.462113000	1.334853000
1	13.157460000	-4.225402000	0.351281000
1	12.582604000	-5.553342000	1.383846000
6	13.665789000	-4.017256000	2.432048000
1	13.230137000	-4.254898000	3.415426000
1	13.802331000	-2.925294000	2.384158000
8	14.884752000	-4.691821000	2.238704000
6	15.848348000	-4.357049000	3.208212000
1	15.497155000	-4.609414000	4.221266000
1	16.069846000	-3.278163000	3.192518000
6	17.104548000	-5.141072000	2.892888000
1	17.453763000	-4.888961000	1.878321000
1	16.880833000	-6.220266000	2.905821000
8	18.066928000	-4.807594000	3.861456000

6	19.282817000	-5.485933000	3.656581000
1	19.726222000	-5.239528000	2.680055000
1	19.153328000	-6.577428000	3.709432000
1	19.970597000	-5.173434000	4.445564000
8	3.788182000	8.274419000	0.057062000
6	3.464149000	9.272450000	-0.892056000
1	3.714129000	8.944126000	-1.909588000
1	2.393599000	9.514196000	-0.858806000
1	4.036494000	10.810959000	0.499205000
6	4.282258000	10.492233000	-0.525735000
1	5.354442000	10.242589000	-0.549941000
8	3.968355000	11.492489000	-1.461774000
6	4.667511000	12.691680000	-1.227053000
1	5.756124000	12.533579000	-1.278736000
1	4.436489000	13.097329000	-0.229640000
6	4.242987000	13.680430000	-2.291894000
1	4.474109000	13.274594000	-3.289485000
1	3.153790000	13.836445000	-2.240686000
8	4.940777000	14.878529000	-2.055348000
6	4.624319000	15.883323000	-2.988465000
1	4.869030000	15.564787000	-4.014107000
1	3.550027000	16.125305000	-2.963092000
1	6.507765000	16.867458000	-2.652094000
6	5.433145000	17.111330000	-2.627939000
1	5.189711000	17.426473000	-1.600174000
8	5.116624000	18.116043000	-3.558430000
6	5.819854000	19.309509000	-3.310241000
1	5.585351000	19.720365000	-2.316695000
1	5.518843000	20.032823000	-4.071426000
1	6.908371000	19.160389000	-3.372059000
8	-8.330532000	3.669810000	-0.089905000

6	-9.294940000	3.321955000	0.885177000
1	-9.519319000	2.247676000	0.850926000
1	-8.941300000	3.570381000	1.894582000
1	-10.307210000	5.197338000	0.584664000
6	-10.539140000	4.121155000	0.561749000
1	-10.886071000	3.875561000	-0.454016000
8	-11.503394000	3.784649000	1.527276000
6	-12.722381000	4.462113000	1.334853000
1	-13.157460000	4.225402000	0.351281000
1	-12.582604000	5.553342000	1.383846000
6	-13.665789000	4.017256000	2.432048000
1	-13.802331000	2.925294000	2.384158000
1	-13.230137000	4.254898000	3.415426000
8	-14.884752000	4.691821000	2.238704000
6	-15.848348000	4.357049000	3.208212000
1	-16.069846000	3.278163000	3.192518000
1	-15.497155000	4.609414000	4.221266000
1	-17.453763000	4.888961000	1.878321000
6	-17.104548000	5.141072000	2.892888000
1	-16.880833000	6.220266000	2.905821000
8	-18.066928000	4.807594000	3.861456000
6	-19.282817000	5.485933000	3.656581000
1	-19.153328000	6.577428000	3.709432000
1	-19.970597000	5.173434000	4.445564000
1	-19.726222000	5.239528000	2.680055000
8	-3.788182000	-8.274419000	0.057062000
6	-3.464149000	-9.272450000	-0.892056000
1	-3.714129000	-8.944126000	-1.909588000
1	-2.393599000	-9.514196000	-0.858806000
1	-4.036494000	-10.810959000	0.499205000
6	-4.282258000	-10.492233000	-0.525735000

1	-5.354442000	-10.242589000	-0.549941000
8	-3.968355000	-11.492489000	-1.461774000
6	-4.667511000	-12.691680000	-1.227053000
1	-5.756124000	-12.533579000	-1.278736000
1	-4.436489000	-13.097329000	-0.229640000
6	-4.242987000	-13.680430000	-2.291894000
1	-4.474109000	-13.274594000	-3.289485000
1	-3.153790000	-13.836445000	-2.240686000
8	-4.940777000	-14.878529000	-2.055348000
6	-4.624319000	-15.883323000	-2.988465000
1	-4.869030000	-15.564787000	-4.014107000
1	-3.550027000	-16.125305000	-2.963092000
1	-6.507765000	-16.867458000	-2.652094000
6	-5.433145000	-17.111330000	-2.627939000
1	-5.189711000	-17.426473000	-1.600174000
8	-5.116624000	-18.116043000	-3.558430000
6	-5.819854000	-19.309509000	-3.310241000
1	-5.585351000	-19.720365000	-2.316695000
1	-5.518843000	-20.032823000	-4.071426000
1	-6.908371000	-19.160389000	-3.372059000
1	0.400033000	-1.022341000	-0.188600000
1	-0.400033000	1.022341000	-0.188600000

Atomic coordinates of global minimum of compound 5

6	0.000000000	3.449714000	-0.014590000
6	3.449709000	0.000000000	0.014629000
6	0.000000000	-3.449714000	-0.014590000
6	-3.449709000	0.000000000	0.014629000
7	1.444217000	-1.443983000	-0.043427000
7	1.443980000	1.444219000	0.043467000

7	-1.444217000	1.443983000	-0.043427000
7	-1.443980000	-1.444219000	0.043467000
6	-2.795944000	1.243665000	0.054133000
6	-1.243165000	2.796629000	0.038849000
6	1.243664000	2.795947000	-0.054092000
6	2.796625000	1.243165000	-0.038809000
6	-2.796625000	-1.243165000	-0.038809000
6	-1.243664000	-2.795947000	-0.054092000
6	1.243165000	-2.796629000	0.038849000
6	2.795944000	-1.243665000	0.054133000
6	-3.466134000	2.507372000	0.227334000
6	-2.505103000	3.468626000	0.216857000
6	2.507371000	3.466135000	-0.227294000
6	3.468624000	2.505103000	-0.216820000
6	3.466134000	-2.507372000	0.227334000
6	2.505103000	-3.468626000	0.216857000
6	-3.468624000	-2.505103000	-0.216820000
6	-2.507371000	-3.466135000	-0.227294000
1	-4.530329000	2.637758000	0.360926000
1	-2.633053000	4.534195000	0.340540000
1	2.637759000	4.530330000	-0.360887000
1	4.534193000	2.633051000	-0.340506000
1	4.530329000	-2.637758000	0.360926000
1	2.633053000	-4.534195000	0.340540000
1	-4.534193000	-2.633051000	-0.340506000
1	-2.637759000	-4.530330000	-0.360887000
6	-0.000597000	4.935664000	-0.026223000
6	-0.638076000	5.649395000	-1.036975000
6	0.651977000	5.649530000	0.994151000
1	-1.139998000	5.115446000	-1.837718000
1	1.139805000	5.094560000	1.787080000

6	-0.628892000	7.045680000	-1.041396000
6	0.669983000	7.036593000	0.999351000
1	-1.126444000	7.576166000	-1.844858000
6	0.018564000	7.751372000	-0.033507000
6	4.935660000	0.000597000	0.026263000
6	5.649391000	0.638055000	1.037027000
6	5.649524000	-0.651957000	-0.994126000
1	5.115443000	1.139962000	1.837780000
1	5.094554000	-1.139769000	-1.787063000
6	7.045677000	0.628870000	1.041447000
6	7.036587000	-0.669963000	-0.999326000
1	7.576164000	1.126406000	1.844919000
6	7.751367000	-0.018566000	0.033544000
6	0.000597000	-4.935664000	-0.026223000
6	-0.651977000	-5.649530000	0.994151000
6	0.638076000	-5.649395000	-1.036975000
1	-1.139805000	-5.094560000	1.787080000
1	1.139998000	-5.115446000	-1.837718000
6	-0.669983000	-7.036593000	0.999351000
6	0.628892000	-7.045680000	-1.041396000
1	1.126444000	-7.576166000	-1.844858000
6	-0.018564000	-7.751372000	-0.033507000
6	-4.935660000	-0.000597000	0.026263000
6	-5.649524000	0.651957000	-0.994126000
6	-5.649391000	-0.638055000	1.037027000
1	-5.094554000	1.139769000	-1.787063000
1	-5.115443000	-1.139962000	1.837780000
6	-7.036587000	0.669963000	-0.999326000
6	-7.045677000	-0.628870000	1.041447000
1	-7.576164000	-1.126406000	1.844919000
6	-7.751367000	0.018566000	0.033544000

30	0.000000000	0.000000000	0.000036000
8	9.099450000	-0.088379000	-0.055625000
6	9.854345000	0.553463000	0.954939000
1	9.655983000	1.631709000	0.981320000
1	9.649274000	0.125023000	1.943408000
1	10.901288000	0.386389000	0.700955000
8	0.088379000	9.099455000	0.055661000
6	-0.553483000	9.854353000	-0.954888000
1	-0.125062000	9.649284000	-1.943366000
1	-1.631729000	9.655993000	-0.981248000
1	-0.386402000	10.901296000	-0.700906000
8	-9.099450000	0.088379000	-0.055625000
6	-9.854345000	-0.553463000	0.954939000
1	-9.655983000	-1.631709000	0.981320000
1	-9.649274000	-0.125023000	1.943408000
1	-10.901288000	-0.386389000	0.700955000
8	-0.088379000	-9.099455000	0.055661000
6	0.553483000	-9.854353000	-0.954888000
1	0.125062000	-9.649284000	-1.943366000
1	1.631729000	-9.655993000	-0.981248000
1	0.386402000	-10.901296000	-0.700906000
8	7.798057000	-1.273796000	-1.944954000
6	7.131601000	-1.979583000	-2.973533000
1	6.496374000	-2.773295000	-2.557888000
1	6.499300000	-1.307064000	-3.568428000
1	8.847522000	-1.786216000	-4.258693000
6	8.208454000	-2.583835000	-3.848972000
1	8.847995000	-3.248957000	-3.248058000
8	7.553299000	-3.290849000	-4.871959000
6	8.449643000	-3.900232000	-5.770241000
1	9.110362000	-4.611950000	-5.250963000

1	9.088508000	-3.151088000	-6.263581000
6	7.623699000	-4.631373000	-6.807084000
1	6.987310000	-5.382265000	-6.312666000
1	6.958985000	-3.919488000	-7.321576000
8	8.517677000	-5.236176000	-7.708985000
6	7.858541000	-5.955149000	-8.723448000
1	7.235821000	-6.759165000	-8.300253000
1	7.198208000	-5.298170000	-9.311371000
1	9.582308000	-7.204787000	-9.033771000
6	8.919536000	-6.551212000	-9.624090000
1	9.542087000	-5.745918000	-10.046893000
8	8.260972000	-7.271664000	-10.635410000
6	9.163477000	-7.868628000	-11.535204000
1	9.786800000	-7.117865000	-12.043855000
1	8.575409000	-8.404819000	-12.283445000
1	9.830326000	-8.582436000	-11.028535000
8	-1.273835000	-7.798066000	1.944966000
6	-1.979644000	-7.131615000	2.973533000
1	-1.307139000	-6.499315000	3.568444000
1	-2.773349000	-6.496388000	2.557874000
1	-3.249023000	-8.848010000	3.248028000
6	-2.583911000	-8.208473000	3.848956000
1	-1.786298000	-8.847543000	4.258686000
8	-3.290942000	-7.553324000	4.871935000
6	-3.900343000	-8.449673000	5.770199000
1	-3.151210000	-9.088548000	6.263543000
1	-4.612057000	-9.110382000	5.250903000
6	-4.631494000	-7.623736000	6.807041000
1	-3.919612000	-6.959034000	7.321552000
1	-5.382373000	-6.987336000	6.312619000
8	-5.236318000	-8.517721000	7.708921000

6	-5.955299000	-7.858590000	8.723384000
1	-5.298320000	-7.198273000	9.311325000
1	-6.759301000	-7.235856000	8.300185000
1	-5.746106000	-9.542159000	10.046807000
6	-6.551387000	-8.919591000	9.624002000
1	-7.204961000	-9.582348000	9.033664000
8	-7.271845000	-8.261034000	10.635321000
6	-7.868831000	-9.163545000	11.535095000
1	-8.582640000	-9.830377000	11.028407000
1	-8.405025000	-8.575481000	12.283337000
1	-7.118082000	-9.786883000	12.043747000
8	1.273835000	7.798066000	1.944966000
6	1.979644000	7.131615000	2.973533000
1	1.307139000	6.499315000	3.568444000
1	2.773349000	6.496388000	2.557874000
1	3.249023000	8.848010000	3.248028000
6	2.583911000	8.208473000	3.848956000
1	1.786298000	8.847543000	4.258686000
8	3.290942000	7.553324000	4.871935000
6	3.900343000	8.449673000	5.770199000
1	3.151210000	9.088548000	6.263543000
1	4.612057000	9.110382000	5.250903000
6	4.631494000	7.623736000	6.807041000
1	3.919612000	6.959034000	7.321552000
1	5.382373000	6.987336000	6.312619000
8	5.236318000	8.517721000	7.708921000
6	5.955299000	7.858590000	8.723384000
1	5.298320000	7.198273000	9.311325000
1	6.759301000	7.235856000	8.300185000
1	5.746106000	9.542159000	10.046807000
6	6.551387000	8.919591000	9.624002000

1	7.204961000	9.582348000	9.033664000
8	7.271845000	8.261034000	10.635321000
6	7.868831000	9.163545000	11.535095000
1	8.582640000	9.830377000	11.028407000
1	8.405025000	8.575481000	12.283337000
1	7.118082000	9.786883000	12.043747000
8	-7.798057000	1.273796000	-1.944954000
6	-7.131601000	1.979583000	-2.973533000
1	-6.496374000	2.773295000	-2.557888000
1	-6.499300000	1.307064000	-3.568428000
1	-8.847522000	1.786216000	-4.258693000
6	-8.208454000	2.583835000	-3.848972000
1	-8.847995000	3.248957000	-3.248058000
8	-7.553299000	3.290849000	-4.871959000
6	-8.449643000	3.900232000	-5.770241000
1	-9.110362000	4.611950000	-5.250963000
1	-9.088508000	3.151088000	-6.263581000
6	-7.623699000	4.631373000	-6.807084000
1	-6.987310000	5.382265000	-6.312666000
1	-6.958985000	3.919488000	-7.321576000
8	-8.517677000	5.236176000	-7.708985000
6	-7.858541000	5.955149000	-8.723448000
1	-7.235821000	6.759165000	-8.300253000
1	-7.198208000	5.298170000	-9.311371000
1	-9.582308000	7.204787000	-9.033771000
6	-8.919536000	6.551212000	-9.624090000
1	-9.542087000	5.745918000	-10.046893000
8	-8.260972000	7.271664000	-10.635410000
6	-9.163477000	7.868628000	-11.535204000
1	-9.786800000	7.117865000	-12.043855000
1	-8.575409000	8.404819000	-12.283445000

1	-9.830326000	8.582436000	-11.028535000
